# Marine Isonitriles and Their Related Compounds

**DOI:** 10.3390/md14010016

**Published:** 2016-01-14

**Authors:** Jens Emsermann, Ulrich Kauhl, Till Opatz

**Affiliations:** Institute of Organic Chemistry, Johannes Gutenberg-University Mainz, Duesbergweg 10-14, 55128 Mainz, Germany; emsermann@uni-mainz.de (J.E.); kauhl@uni-mainz.de (U.K.)

**Keywords:** isonitriles, marine natural products, terpenes, isothiocyanates, formamides, antibiotics, carbonimidic dichlorides, malaria

## Abstract

Marine isonitriles represent the largest group of natural products carrying the remarkable isocyanide moiety. Together with marine isothiocyanates and formamides, which originate from the same biosynthetic pathways, they offer diverse biological activities and in spite of their exotic nature they may constitute potential lead structures for pharmaceutical development. Among other biological activities, several marine isonitriles show antimalarial, antitubercular, antifouling and antiplasmodial effects. In contrast to terrestrial isonitriles, which are mostly derived from α-amino acids, the vast majority of marine representatives are of terpenoid origin. An overview of all known marine isonitriles and their congeners will be given and their biological and chemical aspects will be discussed.

## 1. Introduction

The isonitrile group is a unique functionality as it is isoeletronic to carbon monoxide and shows a high affinity towards transition metals which might cause toxic effects *in vivo*. Moreover, the isonitrile carbon has an uncommon valency state and the exergonic transformation of isonitriles to amides or related structures in countless named or unnamed two- or multi-component reactions such as Passerini- or Ugi-reactions is paramount. Most isonitriles employed in chemical reactions are man-made and are frequently derived from the corresponding amines. On the other hand, the ocurrence of the isonitrile function in natural products is still regarded as a curiosity by many due to its potential reactivity with diverse nucleophiles and electrophiles.

**Figure 1:**
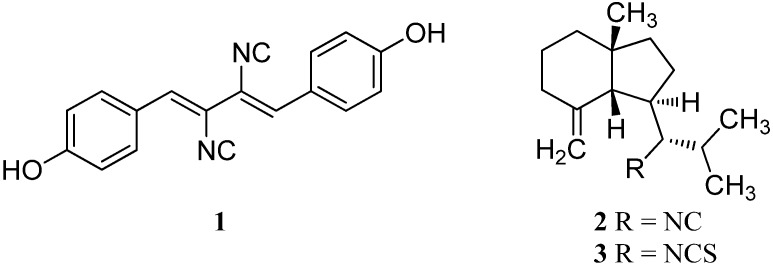
Xanthocillin (**1**), axisonitrile-1 (**2**) and axisothiocyanate (**3**).

Until 1973, the terrestrial isonitrile xanthocillin (**1**) was the only known natural isonitrile which was isolated by Rothe in 1950 from *Penicillium notatum* (Figure 1) [1]. Then, Cafieri *et al.* isolated the first marine isonitrile, axisonitrile-1 (**2**), together with axisothiocyanate-1 (**3**) from the marine sponge *Axinella cannabina* collected in the Bay of Taranto (Italy) [2]. In contrast to the terrestrial isonitriles, which are mostly derived from amino acids, the majority of marine isonitriles have a terpenoid origin. Not much later, other research groups reported the isolation of further marine isonitriles and related compounds and new representatives are constantly being identified [3,4]. To date, about 200 isonitrile natural products are known and 63% of them originate from marine sources.

Early on, Fattorusso *et al.* and Burreson *et al.* observed that the isonitriles occurred usually with the corresponding formamide and isothiocyanate compounds [5,6]. Additionally, structurally closely related cyanates, thiocyanates and carbonimidic dichlorides have been reported, albeit in smaller numbers. The marine isonitriles can be divided into four groups, the sesquiterpenoids (Section 2.1), the diterpenoids (Section 2.2), carbonimidic dichlorides (Section 2.3) and miscellaneous structures (Section 2.4) which can not be classified into the previous groups. The sesquiterpenoids only bear a single nitrogenous functional group and some of them are additionally oxygenated. However, the diterpenoids can possess a higher degree of functionalization. The most complex compounds are the kalihinols which are functionalized with a tetrahydrofuran- or a tetrahydropyran-ring along with hydroxy, chloro or olefinic moieties. Marine isonitriles have been isolated from sponges and from nudibranchs but the latter are probably mostly of dietary origin.

The first review about isonitrile natural products was published by Edenborough and Herbert in 1988 [7] while the first review on marine isonitriles and related compounds by Chang and Scheuer appeared five years later [8]. Chang also published a review on marine and terrestrial isonitriles along with their congeners in 2000 [9]. In the same year Garson *et al.* published a review about the structures, biosynthesis and ecology of marine isonitriles [10]. Reviews about the biosynthesis of marine isonitriles were published by Garson in 1989 [11], by Chang and Scheuer in 1990 [12], and by Garson in 1993 [13]. An update of Garson’s 1988 review appeared in 2004 [14]. A review about isolation, biological activity and chemical synthesis of isocyanoterpenes was published in 2015 by Schnermann and Shenvi [15].

Since the number of known marine-derived isonitrile natural products has increased significantly since 2000, we intend to give the reader a complete overview of all members of this compound class known to date. In addition, aspects about biosynthesis and bioactivity will be discussed.

## 2. Marine Isonitriles and Related Compounds

Most marine isonitriles and their formamide and isothiocyanate congeners are either sesquiterpenoids (Section 2.1) or diterpenoids (Section 2.2). The sesquiterpenoids have only one nitrogenous functional group whereas the diterpenes bear up to three functional groups. Carbonimidic dichlorides (Section 2.3), which originate from the corresponding isonitriles or isothiocyanates, are also known. Finally, there are some miscellaneous structures (Section 2.4) which can not be classified in the previous sections.

### 2.1. Sesquiterpenoids

**Figure 2:**
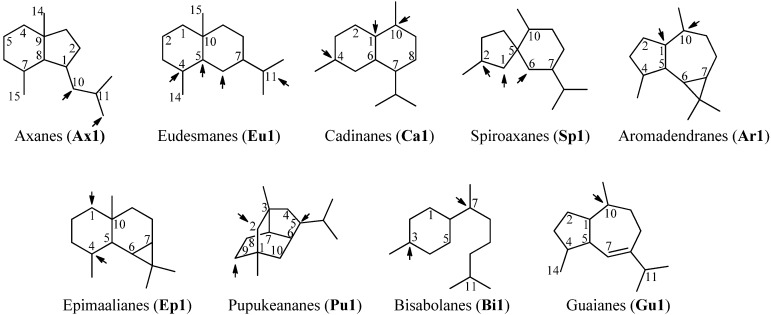
Skeletal-types of sesquiterpenoids (arrows indicate potential attachment points of the nitrogenous function).

Sesquiterpenoids constitute the largest subgroup of the marine isonitriles and their related compounds (–NCS, –NHCHO, –SCN, –NCO, –N=CCl_2_) which in combination could be termed “isonitriloids” due to their close biogenetic relationship with the flagship isonitriles. Most of the isonitriles of this class have the molecular formula C_15_H_25_NC but they can be subdivided into nine groups based on the type of their molecular skeleton (Figure 2). To date, over 190 sesquiterpenes bearing one of the above-mentioned nitrogenous functions are known, among them are nine axanes (**Ax2**–**Ax10**), 32 eudesmanes (**Eu2**–**Eu33**), 33 cadinanes/amorphanes (**Ca2**–**Ca34**), 19 spiroaxanes (**Sp2**–**Sp20**), 14 aromadendranes (**Ar2**–**Ar16**), eight epimaalianes (**Ep2**–**Ep9**), 14 pupukeananes (**Pu2**–**Pu15**), 35 bisabolanes (**Bi2**–**Bi36**), nine guaianes (**Gu2**–**Gu10**) and 17 further sesquiterpenoids (**Fu1**–**Fu17**).

#### 2.1.1. Axanes

**Figure 3:**
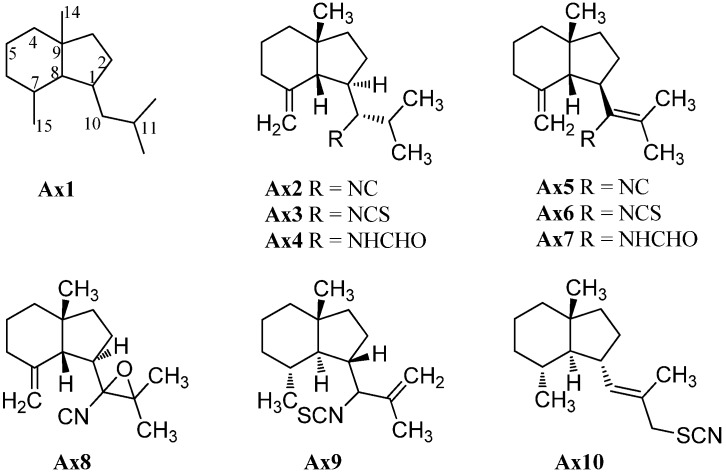
Axanes.

As already mentioned in the introduction, the first marine isonitrile, axisonitrile-1 (**Ax2**), was isolated by Cafieri *et al.* from the marine sponge *Axinella cannabina*, collected in the Bay of Taranto (Italy), along with the corresponding axisothiocyanate-1 (**Ax3**), in 1973 (Figure 3) [2]. The name is based on the genus of the marine sponge, *Axinella cannabina*, and the functional group “isonitrile” or “isothiocyanate”, respectively. The corresponding formamido compound axamide-1 (**Ax4**) was isolated by the same group one year later [5]. Further examination of the extracts of *Axinella cannabina* led to some minor sesquiterpenoids, which could be identified as axisonitrile-4 (**Ax5**), axisothiocyanate-4 (**Ax6**) and axamide-4 (**Ax7**) [16]. The absolute configuration of axisonitrile-1 (**Ax2**), axisonitrile-4 (**Ax5**) and their related compounds was determined by X-ray crystallography and by CD-spectroscopy [17] but the configuration at C-10 was initially incorrectly assigned and had to be revised later [18].

Cavernoisonitrile (**Ax8**), (−)-cavernothiocyanate (**Ax10**) and 10-isothiocyanato-11-axene (**Ax9**) were isolated from the marine sponge *Acanthella cavernosa* and the nudibranch *Phyllidia ocellata*, collected at the Hachijo-jima Island (Japan) by Fusetani *et al.* [19,20].

#### 2.1.2. Eudesmanes

**Figure 4:**
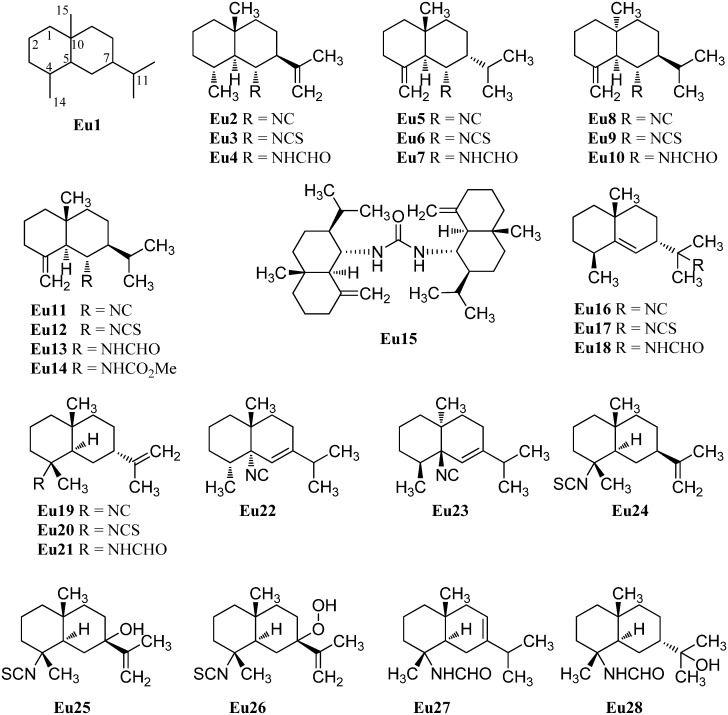
Eudesmanes.

The first eudesmane-type isonitrile, acanthellin-1 (**Eu2**), was isolated from the sponge *Acanthella acuta* by Minale *et al.*, collected in the Bay of Naples, Italy, in 1974 (Figure 4) [3]. The corresponding isothiocyanate **Eu3** and formamide **Eu4** were found together with acanthellin-1 (**Eu2**) ten years later by Ciminiello *et al.* in a sample of *Axinella cannabina* [21]. In the same sample, these authors also detected the isonitrile **Eu5**, the isothiocyanate **Eu6** and the formamide **Eu7** [21]. The same group also reported the isolation of the isonitrile **Eu8**, isothiocyanate **Eu9** and the formamide **Eu10** from the sponge *Axinella cannabina* [22]. Furthermore, the isonitrile **Eu8** and the isothiocyanate **Eu9** were isolated from *Acanthella acuta* [22].

In 1993, Burgoyne *et al.* reported the isolation of acanthene B (**Eu12**) from an *Acanthella* sponge and acanthene C (**Eu13**) from the nudibranch *Cadlina luteomarginata* but they were unable to isolate the parent isonitrile **Eu11** [23]. However, this compound, halichonadin C (**Eu11**), was obtained from the sponge *Halichondria* sp. from Unten Port, Okinawa, Japan [24]. In this sample, Ishiyama *et al.* also found halichonadin B (**Eu14**) with a carbamate group and halichonadin A (**Eu15**), a dimer with two eudesmane skeletons bridged by a urea linkage [24].

The previous compounds all carry their nitrogenous function at C-6 but compounds with the function at C-4 and C-11 are also known. 11-Isocyano-7β-*H*-eudesm-5-ene (**Eu16**), the corresponding 11-isothiocyano-7β-*H*-eudesm-5-ene (**Eu17**), as well as 11-formamido-7β-*H*-eudesm-5-ene (**Eu18**) were isolated by Ciminiello *et al.* in 1987 from the sponge *Axinella cannabina* [25]. The compounds were also found in the Caribbean sponge *Axinyssa ambrosia* [26]. Moreover the isonitrile **Eu16** was obtained from the nudibranch *Phyllidiella pustulosa* [27] and together with the isothiocyanato compound **Eu17** from the sponge *Acanthella* sp. and the nudibranch *Cadlina luteomarginata* [23]. The isothiocyanato compound **Eu17** is also one of several sesquiterpenoids isolated from the sponge *Acanthella pulcherrima* [28]. 11-Isothiocyano-7β-*H*-eudesm-5-ene (**Eu17**) did not show cytotoxity against cultured KB-3 cells (IC_50_ > 20 µg/mL) but moderate antimalarial activity against *Plasmodium falciparum* was observed (chloroquine-sensitive strain D6: IC_50_ = 2240 ng/mL, chloroquine-resistant strain W2: IC_50_ = 610 ng/mL) [29].

4-Isocyanatoeudesm-11-ene (**Eu19**) and 4-formamidoeudesm-11-ene (**Eu21**) have been isolated from the Caribbean sponge *Axinyssa ambrosia* [26]. The appropriate 4-isothiocyanatoeudesm-11-ene (**Eu20**) was found in the sponge *Acanthella klethra* [30] and later also in an unidentified sponge of the genus *Acanthella*, the nudibranch Ca*dlina luteomarginata* [23], the sponge *Acanthella cavernosa* [31,32] and the sponge *Axinyssa Isabela* [33]. Similar to isothiocyanate **Eu17**, 4-isothiocyanatoeudesm-11-ene (**Eu20**) displayed a moderate antimalarial activity against *Plasmodium falciparum* (chloroquine-sensitive strain D6: IC_50_ = 4000 ng/mL, chloroquine-resistant strain W2: IC_50_ = 550 ng/mL) and no cytotoxic activity against KB-3 cells (IC_50_ > 20 µg/mL) [29].

Stylostelline (**Eu22**) ([α]_D_ = +23° (CHCl_3_, c = 2)), isolated from the *Stylotella* sp. and collected in the Southeast of New-Caledonia [34], is one of only two marine isonitriloids with the nitrogenous function attached to C-5. The other known isonitrile is the corresponding enantiomer *ent*-stylotelline (**Eu23**) ([α]_D_ = −47° (CHCl_3_, c = 1.7)) which was isolated from the nudibranch *Phyllidiella pustulosa* [35].

The isothiocyanate **Eu24** is a C-7 epimer of compound **Eu20** and was also isolated from the marine sponge *Acanthella klethra* [30]. The inversion of configuration at C-7 leads to a drastic reduction of the antimalarial activity against *Plasmodium falciparum* (chloroquine-sensitive strain D6: IC_50_ > 10 µg/mL, chloroquine-resistant strain W2: IC_50_ > 10 µg/mL) [29].

With only few exceptions, the nitrogenous function is the only heteroatom moiety in these secondary metabolites. However, Zubia *et al.* investigated the metabolites of the sponge of the genus *Axynissa*, collected in the Gulf of California (Mexico). In these samples, the hydroxylated axinisothiocyanate M (**Eu25**) and the hydroperoxide axinisothiocyanate N (**Eu26**) were identified [33].

In 2008, Lan *et al.* investigated extracts of the sponge *Axinyssa* sp., collected from the South Chinese Sea, and isolated the hitherto unknown formamides 4-formamidoeudesm-7-ene (**Eu27**) and the hydroxylated 4-formamidoeudesman-11-ol (**Eu28**) [36]. 4-Formamidoeudesm-7-ene (**Eu27**) exhibited significant cytotoxic activity against the human cancer cell lines CNE-2 (IC_50_ = 13.8 µg/mL), HeLa (IC_50_ = 7.5 µg/mL) and LO2 (IC_50_ = 38.0 µg/mL) [36].

**Figure 5:**
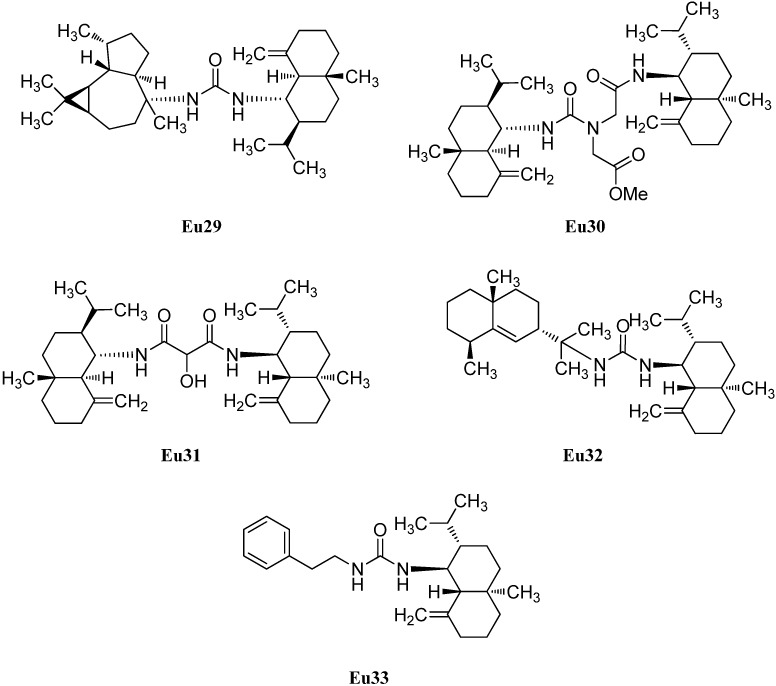
Dimeric sesquiterpenoids with eudesmane skeleton.

In addition to halichonadin A (**Eu15**), there are some other dimeric sesquiterpenoids with the eudesmane skeleton (Figure 5). Halichonadin E (**Eu29**) is an unsymmetrical dimer with one eudesmane and one aromadendrane (see Section 2.1.5) unit linked by a bridging urea moiety and was isolated from the *Halichondria* sp. by Kozawa *et al.* [37]. The same group isolated and identified the halichonadins G (**Eu30**), H (**Eu31**), I (**Eu32**) and J (**Eu33**) [38]. The halichonadins G–I are all dimeric sesquiterpenoids with two eudesmane skeletons with an 2-[1-(2-amino-2-oxoethyl)ureido]acetate linkage (**Eu30**), a 2-hydroxymalonamide linkage (**Eu31**) and an urea linkage (**Eu32**). Halichonadin J (**Eu33**) is a sesquiterpenoid with an eudesmane skeleton connected to 2-phenethylamine through a urea bridge.

#### 2.1.3. Cadinanes

**Figure 6:**
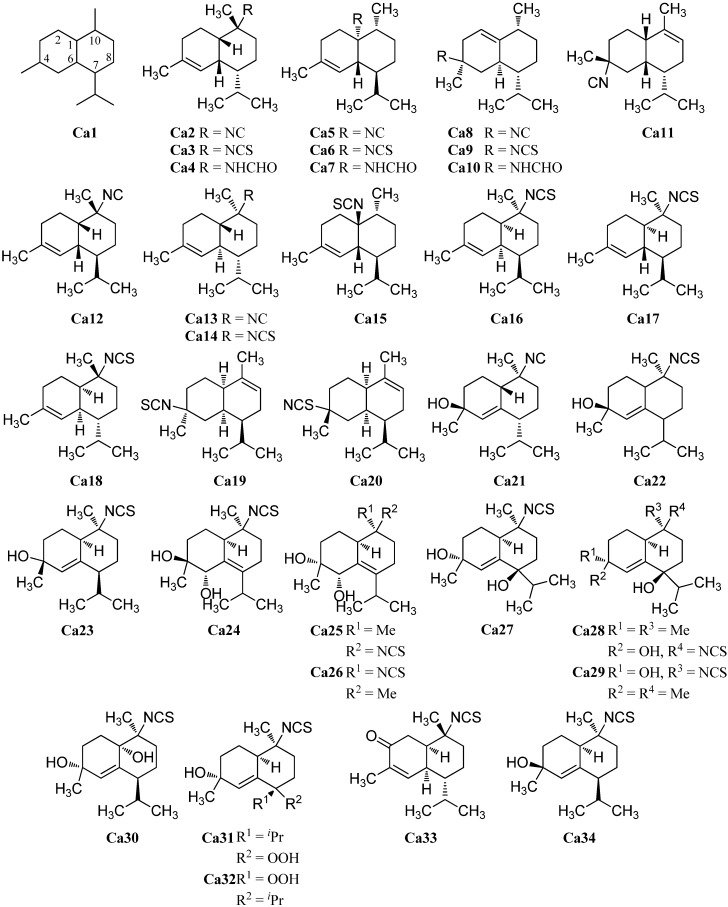
Cadinanes.

The first cadinane- or amorphane-type isonitrile **Ca2** was isolated along with the corresponding 10-isothiocyanato-4-amorphene (**Ca3**) and 10-formamido-4-amorphene (**Ca4**) from the sponge *Halichondria* sp. by Burreson *et al.* in 1975 (Figure 6). [6,39,40].

10-Isothiocyanato-4-amorphene (**Ca3**) displayed a weak antiplasmodial activity against *Plasmodium falciparum* (strains K1 and NF54, IC_50_ = 5.7 µM) [41]. Furthermore, 10-isocyano-4-amorphene (**Ca2**, IC_50_ = 7.2 µg/mL) and 10-isothiocyanato-4-amorphene (**Ca3**, IC_50_ = 0.70 µg/mL) showed potent antifouling activity against larvae of the acorn barnacle *Balanus amphitrite* [42]. Later, the isonitrile **Ca2** was isolated from a sponge of the genus *Axinyssa* [43] and along with the isothiocyanate **Ca3** from nudibranchs of the genus *Phyllidiidae* [42].

Further investigation by Ciminiello *et al.* of extracts from the sponge *Axinella cannabina* led to the identification of minor metabolites isonitrile **Ca5**, isothiocyanate **Ca6** and the formamide **Ca7** [44]. The isothiocyanate compound halipanicine (**Ca9**) was found by Nakamura *et al.* in the sponge *Halichondria panacea*, collected from Okinawan Island, Japan [45]. The corresponding isonitrile **Ca8** and formamide **Ca10** were absent in this sponge and were isolated later from the sponge *Axinyssa aplysinoides* from Palau [46].

There are numerous cadinanes for which the complete isonitriloid triples of isonitrile, isothiocyanate, and formamide have not yet been found but where only one of the isonitriloid species is known. The isonitrile 4α-isocyano-9-amorphene (**Ca11**) was isolated by Fusetani *et al.* from specimens of the nudibranch *Phyllidia pustulosa*, which were collected off Hachijo-jima Island, Japan [47]. The same team isolated the isonitrile 10α-isocyano-4-amorphene (**Ca12**) from the sponge *Acanthella* cf. *cavernosa* and the nudibranch *Phyllidia ocellata* [19]. 10-Isocyano-4-cadinene (**Ca13**) was isolated by Okino *et al.* from the nudibranch *Phyllidia pustulosa* from Kamikoshiki-jima Island, Japan, in 1996 [42]. This isonitrile also displayed potent antifouling activity against larvae of the acorn barnacle *Balanus amphitrite* (IC_50_ = 0.14 µg/mL) [42]. Four years later, the corresponding 10-isothiocyanato-4-cadinene (**Ca14**) was isolated from the tropical marine sponge *Acanthella cavernosa* [31]. Its absolute configuration was elucidated by enantioselective synthesis of both optical antipodes [48]. The isothiocyanate **Ca15**, a C-1 epimer of isothiocyanate **Ca6**, was isolated from a specimen of the sponge *Acanthella pulcherrima*, collected off Weed Reef, Darwin, Australia [28]. The isothiocyanate (1*R*,6*S*,7*S*,10*S*)-10-isothiocyanato-4-amorphene (**Ca16**) ([α]_D_ = +100° (CCl_4_, c = 5.5)), the enantiomer of **Ca3** ([α]_D_ = –63° (CCl_4_, c = 7.4)), was isolated from the sponges *Axinella fenestratus*, *Topsentia* sp. and *Acanthella cavernosa* [49]. Mitome *et al.* reported the isolation of (1R*,6R*,7S*,10S*)-10-isothiocyanatocadin-4-ene (**Ca17**) from the Okinawan marine sponge *Stylissa* sp. [50]. Axinisothiocyanate K (**Ca18**) was isolated by Zubia *et al.* from a sponge of the genus *Axinyssa*, collected in the Gulf of California in 2008 [51]. (1R*,4S*,6R*,7S*)-4-Isothiocyanato-9-amorphene (**Ca19**) was obtained from the Fijian sponge *Axinyssa fenestratus* and the Thailand sponges *Topsentia* sp. and *Acanthella cavernosa* [49].

He *et al.* isolated in 1989 the unusual and first marine thiocyanate **Ca20** [52]. The relative stereochemistry of (1S*,4S*,6S*,7R*)-4-thiocyanato-9-cadinene (**Ca20**) was determined by X-ray analysis [52]. The biogenetic relation of the thiocyanates to the classical isonitriloids remains unclear.

As for the eudesmanes (see Section 2.1.2), several hydroxylated and hydroperoxide-funtionalized cadinane sesquiterpenoids are known. The first isolated hydroxylated cadinane-type sesquiterpenoid was (1S*,4S*,7R*,10S*)-10-isocyano-5-cadinen-4-ol (**Ca21**), isolated from the nudibranch *Phyllidia pustulosa* [53] while the closely related 10-isothiocyanatoamorph-5-en-4-ol (**Ca22**) was obtained from the sponges *Axinella fenestratus*, *Topsentia* sp. and *Acanthella cavernosa* [49]. (1S*,4S*,7R*,10S*)-10-Isocyano-5-cadinen-4-ol (**Ca21**) showed antifouling activity against larvae of the barnacle *Balanus amphitrite* (EC_50_ = 0.17 µg/mL) [53].

In 2008, Zubia *et al.* investigated specimens of the marine sponge *Axinyssa* sp. and were able to isolate as many as twelve new cadinane-type sesquiterpenoids, the axinisothiocyanates A–L [51]. For the non-oxygenated axinisothiocyanate K (**Ca18**), *vide supra*. Axinisothiocyanate J (**Ca23**) carries a hydroxy group at C-4. In contrast, axinisothiocyanates A (**Ca24**), B (**Ca25**) and C (**Ca26**) have two hydroxy groups at C-4 and C-5 and differ in their relative configuration. The same applies to axinisothiocyanate D (**Ca27)**, axinisothiocyanate E (**Ca28**) and axinisothiocyanate F (**Ca29**) with the hydroxy groups at C-4 and C-7.

Another dihydroxylated isonitriloid, this time oxygenated at C-4 and at C-1, is axinisothiocyanate G (**Ca30**). Furthermore, two isothiocyanates with one hydroxy group at C-4 and one hydroperoxy group at C-1, axinisothiocyanates H (**Ca31**) and I (**Ca32**) were isolated from the same source. The last member of the series, axinisothiocyanate L (**Ca33**), is characterized by a keto group at C-3.

Axiplyn C (**Ca34**) was isolated from the sponge *Axinyssa aplysinoides*, collected at Misali Island, Tanzania [54].

#### 2.1.4. Spiroaxane

**Figure 7:**
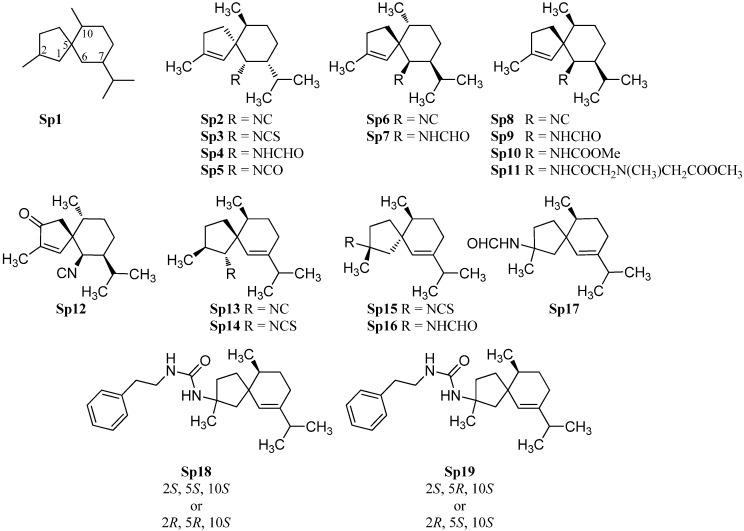
Spiroaxanes.

Di Blasio *et al.* isolated in 1976 the first spiroaxane-type isonitrile (+)-axisonitrile-3 (**Sp2**) ([α]_D_ = +68.4° (CHCl_3_, c = 1) along with (+)-axisothiocyanate-3 (**Sp3**) ([α]_D_ = +165.2° (CHCl_3_, c = 1)) and (−)-axamide-3 (**Sp4**) ([α]_D_ = −6.86° (CHCl_3_, c = 1)) from the marine sponge *Axinella cannabina* (Figure 7) [55]. Their relative configuration was determined by X-ray crystallography while their absolute stereochemistry was determined by total synthesis of the enantiomeric (−)-axisontrile-3 (**Sp6**), the absolute configuration of which proved to be (5*S*,6*R*,7*S*,10*R*) [56]. The three compounds were also isolated from other sponges like *Acanthella acuta* [57], *Acanthella cavernosa*[19] and the nudibranchs *Phyllidia ocellata* [19] and *Phyllidia pustulosa* [42], (+)-axisonitrile-3 (**Sp2**) exhibited strong antimalarial activity against *Plasmodium falciparum* (chloroquine-sensitive strain D6: IC_50_ = 142 ng/mL, chloroquine-resistant strain W2: IC_50_ = 16.5 ng/mL) without cytotoxic activity against KB-3 cells (IC_50_ > 20 µg/mL). The corresponding isothiocyanate **Sp3** is 500-fold less active than the isonitrile **Sp2** (strain D6: IC_50_ = 12,340 ng/mL, strain W2: IC_50_ = 3,110 ng/mL) [29].

In extracts of the sponge *Acanthella cavernosa* was also found one of the few known marine isocyanates, axisocyanate-3 (**Sp5**) [58]. The enantiomeric (−)-axisonitrile-3 (**Sp6**) ($\alpha_{D}^{26}$ = −79° (CHCl_3_, c = 1.93)) and (+)-axamide (**Sp7**) ($\alpha_{D}^{26}$ = +17.5° (CHCl_3_, c = 3.05)) were obtained from the sponge *Halichondria* sp., collected off Phi Phi Island in the Andaman Sea, Southern Thailand [59].

10-*epi*-Axisonitrile-3 (**Sp8**), a C-10 epimer of axisonitrile-3 (**Sp6**), has been isolated from the nudibranch *Phyllidia pustulosa* [42]. Both isonitriles exhibited potent antifouling activity against larvae of the acorn barnacle *Balanus amphitrite* (**Sp6**: IC_50_ = 3.2 µg/mL, **Sp8**: IC_50_ = 10 µg/mL) [42]. Together with the known isonitrile **Sp8**, exiguamide (**Sp9**), exicarbamate (**Sp10**) and exigurin (**Sp11**) were isolated from the sponge *Geodia exigua* [60]. 3-Oxoaxisonitrile-3 (**Sp12**) was obtained from an unidentified Chinese marine sponge of the genus *Acanthella* [61].

In contrast to the above-mentioned spiroaxanes **Sp2**–**Sp12**, the isonitrile **Sp13** and the isothiocyanate **Sp14** both carry the nitrogenous functional group at C-1. Both spiroaxanes were isolated from the marine sponge *Acanthella acuta* [62].

(2*R*,5*R*,10*S*)-2-Isothiocyanato-6-axene (**Sp15**) and (2*R*,5*R*,10*S*)-2-formamido-6-axene (**Sp16**) with the functional group at C-2 were isolated by He *et al.* from the Palauan sponge *Trachyopsis aplysinoides* [52]. However, they identified the relative configuration of the both compounds as (2*R*,5*R*,10*R*) with a stereochemistry at C-10 unusual for natural products. Wegerski *et al.* revised the configuration from (2*R*,5*R*,10*R*)- to (2*R*,5*R*,10*S*)-2-isothiocyanato-6-axene (**Sp15**) and (2*R*,5*R*,10*S*)-2-formamido-6-axene (**Sp16**) [63]. During their investigations, the group also found the isothiocyanate **Sp17** with (2*S*,5*R*,10*S*)- or (2*R*,5*S*,10*S*)-configuration [63]. Furthermore they found in analogy to halichonadin J (**Eu30**), *vide supra* the compounds *N*-phenethyl-2-formamido-6-axene (**Sp18**) with either (2*S*,5*S*,10*S*) or (2*R*,5*R*,10*S*) configuration and *N*-phenethyl-2-formamido-6-axene (**Sp19**) with either (2*S*,5*R*,10*S*) or (2*R*,5*S*,10*S*) configuration [63].

#### 2.1.5. Aromadendranes

**Figure 8:**
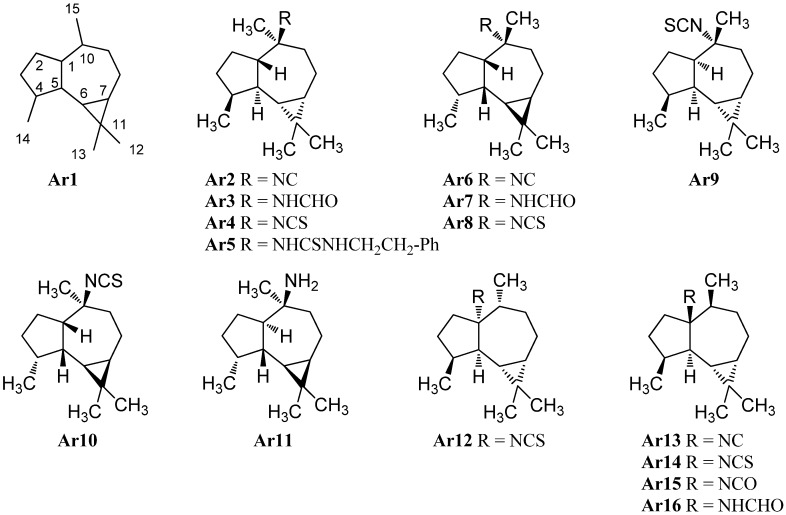
Aromadendranes.

Another type of sesquiterpene isonitriles is based on the aromadendrane-skeleton (**Ar1**) (Figure 8). The first example of this class was isolated directly in the early days of the discovery of marine isonitriles shortly after axisonitrile-1 (**Ax2**). It was found in the Mediterranean sponge *Axinella cannabina* collected in the bay of Taranto, Italy by Fattorusso *et al.* in 1974 and was named axisonitrile-2 (**Ar2**) [4,5]. Its relative stereochemistry was elucidated by Ciminiello twelve years later [25]. Together with axisonitrile-2 (**Ar2**), Fattorusso *et al.* were also able to isolate the corresponding formamide axamide-2 (**Ar3**) and the isothiocyanate congener axisothiocyanate-2 (**Ar4**) from the same source[5]. Axisonitrile-2 (**Ar2**) was later also found by Hirota in the sponge *Acanthella cavernosa* [47]. In 1985 Tada reported the isolation of the isothiocyanate epipolasin B (**Ar4**) ([α]_D_ = + 91.2 (CHCl_3_, c = 1.0)) and its β-phenylethylamine adduct epipolasinthiourea-B (**Ar5**) in extracts of *Epipolasis kushimotoensis* [64]. Epipolasin B has the same 2D-structure as axisothiocyanate-2 (**Ar4**), however, based on differences in the optical rotation compared to Fattorusso’s compound **Ar4** ([α]_D_ = + 12.8 (CHCl_3_, c = 1.5)), Tada proposed that both substances differ in stereochemistry. Chemical synthesis of both isothiocyanate enantiomers by da Silva makes a contamination of Fattorusso’s compound likely which may have caused a wrong optical rotation and suggests both isolated substances to be in fact identical [65]. Epipolasinthiourea-B (**Ar5**) showed moderate cytotoxic activities against the murine L1210 leukemia cell line (ED_50_ = 3.7 µg/mL) [64].

Examination of *Acanthella cannabina* collected off the coast of Taranto near Porto Cesareo (Italy) by Ciminiello *et al.* in 1987 resulted in the isolation of 10α-isocyanoalloaromadendrane (**Ar6**), 10α-formamidoalloaromadendrane (**Ar7**) and 10α-isothiocyanatoalloaromadendrane (**Ar8**), an isonitriloid triad showing a C1-epimeric alloaromadendrane-skeleton compared to **Ar2**, **Ar3** and **Ar4** [25].

An isothiocyanate with an opposite stereochemistry with the exception of C-10, (1*R*,4*S*,5*S*,6*R*,7*S*,10*R*)-(+)-isothiocyanatoalloaromadendrane (**Ar9**), was found in 1996 by Hirota *et al.* in an *Acanthella cavernosa* from Hachijo-jima Island (Japan) [20]. The absolute configuration was elucidated by comparison with the corresponding alcohols synthesized from (−)-alloaromadendrene. The compound was isolated again in 2010 by Lyakhova *et al.* from the Vietnamese nudibranch *Phylidiella pustulosa* [27]. The enantiomer **Ar10** of this compound was prepared synthetically in 1994 by da Silva *et al.* and allowed the determination of the absolute configuration [65]. **Ar9** and axisothiocyanate-2 (**Ar4**) were found to display potent antifouling activity against cyprid larvae of the acorn barnacle *Balanus amphitrite* [20].

Investigation of a *Halichondria* sp. collected near Unten Port (Okinawa, Japan) by Ishiyama *et al.* in 2008 led to the isolation of halochonadin F (**Ar11**), an amine epimeric at C-1 to da Silva’s compound . It was found to exhibit antimicrobial activity against *Micrococcus luteus* (MIC = 4 µg/mL), *Trichophyton mentagrophytes* (MIC 8 µg/mL) and *Cryptococcus neoformans* (MIC 16 µg/mL) [66].

In 1988, Breakman reported the isolation of an isonitrile, an isothiocyanate, and an isocyanate from the sponge *Acanthella acuta* (Banyuls, France) bearing the nitrogen functionality at C-1 instead of C-10 of the aromadendrane skeleton. Due to a misassigned reference substance, a wrong relative configuration was initially published [9,57], which was corrected two years later [67]. The first two compounds were found in parallel in an *Acanthella acuta* sponge collected in the Bay of Naples (Italy) by Mayol who elucidated the correct relative stereochemistry of **Ar13** and **Ar14** [62].

The formamide **Ar16** was discovered in 2007 by Zhang *et al.* in the Spanish dancer nudibranch *Hexabrandies sanguinens* collected in the South China Sea [68]. The isolation of the corresponding isocyanate **Ar15** was reported in 2007 by Jumaryatno from an Australian specimen of *Acanthella cavernosa* (Coral gardens, Gneerings reef, Mooloolaba) [32]. In 1992, He and Faulkner were able to identify the C1/C10-epimer **Ar12** of isothiocyanate **Ar14** in a sponge *Axinyssa aplysinoides* collected at Ant Atoll (Pohnpei/Micronesia) [69].

#### 2.1.6. Epimaalianes

**Figure 9:**
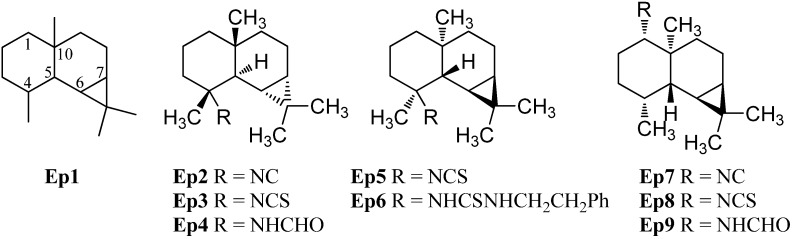
Epimaalianes.

The epimaaliane sesquiterpenoids have a similar skeleton to the eudesmanes with a methyl group in C-4 and C-10 position but they possess an additional bond between C-6 and C-11, creating a cyclopropane ring (**Ep1**, Figure 9).

The first isolated epimaaliane-type sesquiterpenoids were the unnamed isonitrile **Ep2** and (−)-epipolasin A (**Ep3**) ([α]_D_ = −12° (CHCl_3_, c = 1.1)) isolated from the nudibranch *Cadlina luteomarginata* by Thompson *et al.* in 1982 [70]. Their relative stereochemistry was determined by X-ray crystallography of the formamide **Ep4** which was obtained by hydrolysis of the isonitrile **Ep2** [70]. Later, (−)-epipolasin A (**Ep3**) was isolated from the sponges *Acanthella pulcherrima* [28] and *Axinyssa* sp. nov. [71] and along with the isonitrile **Ep2** and the formamide **Ep4** from a Northeastern Pacific *Acanthella* sp. [23]. The isonitrile **Ep2** and the formamide **Ep4** were also isolated from skin extracts of the nudibranch *Cadlina luteomarginata* while the corresponding (−)-epipolasin A was not found in this organism [23]. (+)-Epipolasin A (**Ep5**) ([α]_D_ = +7.6° (CHCl_3_, c = 1)) was found in the sponge *Epipolasis kushimotoensis* by Tada and Yasuda [64]. The absolute stereochemistry was deduced by correlation of ECD-spectra with those of the naturally occurring maaliol. The related isonitrile and formamide were not found while the thiourea compound **Ep6** was isolated from the same producer [64]. (+)-Epipolasin A (**Ep5**) shows activity against *Plasmodium falciparum* strains D6 (IC_50_ = 5600 ng/mL) and W2 (IC_50_ = 5550 ng/mL) whithout exhibiting cytotoxic activity against KB cells (IC_50_ > 20 µg/mL) [71].

The isonitrile **Ep7**, the isothiocyanate **Ep8** and the formamide **Ep9** with the functional group attached to C-1 instead of C-4 were found during further investigations of extracts of the sponge *Axinella cannabina* by Ciminiello *et al.* [72].

#### 2.1.7. Pupukeananes

**Figure 10:**
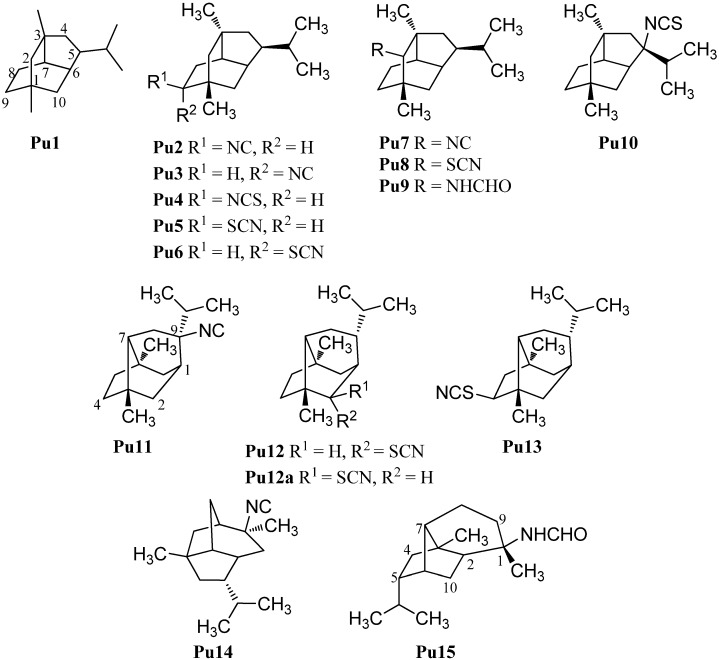
Pupukeananes.

A further group of sesquiterpenoids are the pupukeananes which bear a tricyclo[4.3.1.0^3,7^]decane skeleton (**Pu1**, Figure 10). The first isolated pupukeanane was 9-isocyanopupukeanane (**Pu2**) which was obtained from the nudibranch *Phyllidia varicosa* and an unidentified sponge of the genus *Hymeniacidon* by Burreson *et al.* in 1975 [73]. Later, *Hymeniacidon* sp. was reclassified as a *Ciocalypta* sp. [74]. The corresponding C-9 epimer 9-*epi*-isocyanopupukeanane (**Pu3**) was isolated from the nudibranch *Phyllidia bourguini* along with 9-isocyanopupukeanane (**Pu2**) [75]. Later, the corresponding 9-isothiocyanatopupukeanane (**Pu4**) was found in the sponge *Axinyssa* sp. nov., collected at the Great Barrier Reef, Australia [71]. 9-Isocyanopupukeanane (**Pu2**) displayed a weak antimalarial activity against *Plasmodium falciparum* (strain D6: IC_50_ = 2520 ng/mL, strain W2 IC_50_ = 1610) and showed no activity against KB cells (IC_50_ > 20 µg/mL). In contrast to isonitrile **Sp2** and isothiocyanatate **Sp3**, the antimalarial activity of the corresponding isothiocyanate **Pu4** was similar to isonitrile **Pu2** (strain D6: IC_50_ = 3290 ng/mL, strain W2: IC_50_ = 890 ng/mL) [71].

Remarkably and in sharp contrast to the other sesquiterpenoid subgroups, numerous thiocyanates with a pupukeanane skeleton are known. 9-Thiocyanatopupukeanane (**Pu5**) and its C-9 epimer 9-*epi*-thiocyanatopupukeanane (**Pu6**) were isolated from the nudibranch *Phyllidia varicosa* and the sponge *Axinyssa aculeata*, collected from the coral reefs of Pramuka Island, Indonesia [76]. Both compounds **Pu5** (IC_50_ = 4.6 µg/mL) and **Pu6** (IC_50_ = 2.3 µg/mL) showed potent antifouling activity against larvae of the acorn barnacle *Balanus amphitrite* [42].

In addition to the C-9 functionalized pupukeananes, some C-2 and C-5 functionalized representatives of this class ar known as well. 2-Isocyanopupukeanane (**Pu7**) was obtained by Hagadone *et al.* from the nudibranch *Phyllidia varicosa* together with isonitrile **Pu2** [77]. He *et al.* isolated the corresponding 2-thiocyanatopupukeanane (**Pu8**) from the sponge *Axinyssa aplysinoides* [69]. Recently the corresponding formamide **Pu9** was isolated from *Phyllidia coelestis* Bergh, collected near the Koh-Ha Islets, Thailand [78].

Furthermore, one pupukeanane with an isothiocyanate group at C-5 (**Pu10**) was obtained by Marcus *et al.* from a sponge of the genus *Axinyssa* [43].

In addition, there are some rearranged pupukeananes like 9-isocyanoneopupukeanane (**Pu11**), which was obtained from the sponge *Ciocalypta* sp. collected in O’ahu, Hawaii [79]. 2-Thiocyanatoneopupukeanane (**Pu12**) was first isolated at 1991 by Pham *et al.* from an unidentified Phonpei sponge but they determined the configuration of the functional group at C-2 incorrectly (**Pu12a**) [80]. One year later He *et al.* also isolated the thiocyanato compound **Pu12** from the sponge *Axinyssa aplysinoides* and revised the configuration at C-2 on the basis of NMR experiments [69]. 2-Thiocyanatoneopupukeanane (**Pu12**) showed a similar antimalarial activity against *Plasmodium falciparum* (strain D6: IC_50_ = 4700 ng/mL, strain W2: IC_50_ = 890 ng/mL) as 9-isocyanopupukeanane (**Pu2**) and 9-isothiocyanatopupukeanane (**Pu4**) [71]. 4-Thiocyanatoneopupukeanane (**Pu13**) was also isolated by Pham *et al.* from the sponge *Phycopsis terpnis* [80].

Apart from the pupukeananes (**Pu2**–**Pu10**) and the neopupukeananes (**Pu11**–**Pu13**), two other rearranged pupukeananes are known: 2-Isocyanoallopupukeanane (**Pu14**) was obtained from the nudibranch *Phyllidia pustulosa* by Fusetani *et al.* [47] and Jaisamut *et al.* isolated 1-formamido-10(1→ 2)-abeopupukeanane (**Pu15**) from the nudibranch *Phyllidia coelestis* Bergh [78].

#### 2.1.8. Bisabolanes

**Figure 11:**
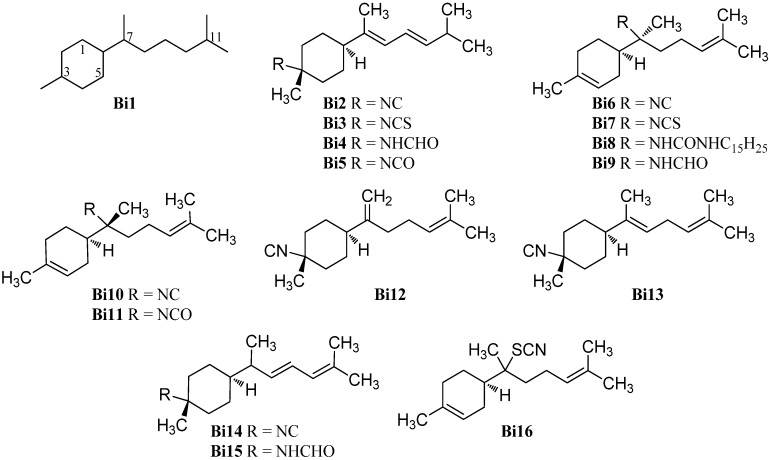
Bisabolanes.

Bisabolane-type compounds have a 1-methyl-4-(6-methyl-2-heptanyl)cyclohexane skeleton (**Bi1**) and the functional group is either located at C-3 or at C-7 (Figure 11). The first isolated bisabolane compounds were 3-isothiocyanatotheonellin (**Bi3**) and 3-formamidotheonellin (**Bi4**) from the Okinawan sponge *Theonella* cf. *swinhoei* reported by Nakamura *et al.* in 1984 [81]. The analog 3-isocyanotheonellin (**Bi2**) was isolated two years later by Gulavita *et al.* from the nudibranch *Phyllidia* sp. [74]. 3-Isocyanotheonellin (**Bi2**) showed potent antifouling activity against larvae of the acorn barnacle *Balanus amphitrite* (IC_50_ = 0.13 µg/mL) [42]. In 2012 Wright *et al.* reported the isolation and characterization of 3-isocyanatotheonellin (**Bi5**) together with the known isonitrile **Bi2**, isothiocyanate **Bi3** and 7-isothiocyanato-7,8-dihydro-α-bisabolene (**Bi7**) from the sponge *Raphoxya* sp., collected near Blue Hole, Guam [82].

The latter compound was also found in the sponge *Halichondria* sp. together with the urea derivate *N*,*N*′-bis((6*R*,7*S*)-7,8-dihydro-α-bisabolen-7-yl)urea (**Bi8**) in 1986 [83]. The related 7-isocyano-7,8-dihydro-α-bisabolene (**Bi6**) was isolated from the tropical marine sponge *Acanthella cavernosa* by Jumaryatno *et al.* in 2007 [32] and 7-formamido-7,8-dihydro-α-bisabolene (**Bi9**) was isolated from the Hainan marine sponge *Axinyssa* sp. by Li *et al.* in 2008 [84].

In 1986, Gulavita *et al.* also reported the isolation of 7-isocyano-7,8-dihydro-α-bisabolene (**Bi10**), the C-7 epimer of the isonitrile **Bi6**, and 7-isocyanato-7,8-dihydro-α-bisabolene (**Bi11**) from the sponge *Ciocalypta* sp. [74].

The isonitrile **Bi12** with an *exo*-methylene group at C-7 was obtained from the nudibranch *Phyllidiella pustulosa* from the South China Sea [35]. (*E*)-4-Isocyanobisabolane-7,10-diene (**Bi13**) with two non-conjugated double bonds was isolated from an Okinawan sponge of the genus *Axinyssa* [85].

Kassuehlke *et al.* reported the isolation of 3-isocyanobisabolane-8,10-diene (**Bi14**) from the nudibranch *Phyllidia pustulosa* and the appropriate 3-formamidobisabolene-8,10-diene (**Bi15**) from the Paulauan sponge *Halichondria* cf. *lendenfeldi*. However, the full characterization of the formamide **Bi15** was not possible due to decomposition [86].

The thiocyanate axinythiocyanate A (**Bi16**) was obtained from the sponge *Axinyssa isabela*, collected in the Gulf of California, Mexico [33].

**Figure 12:**
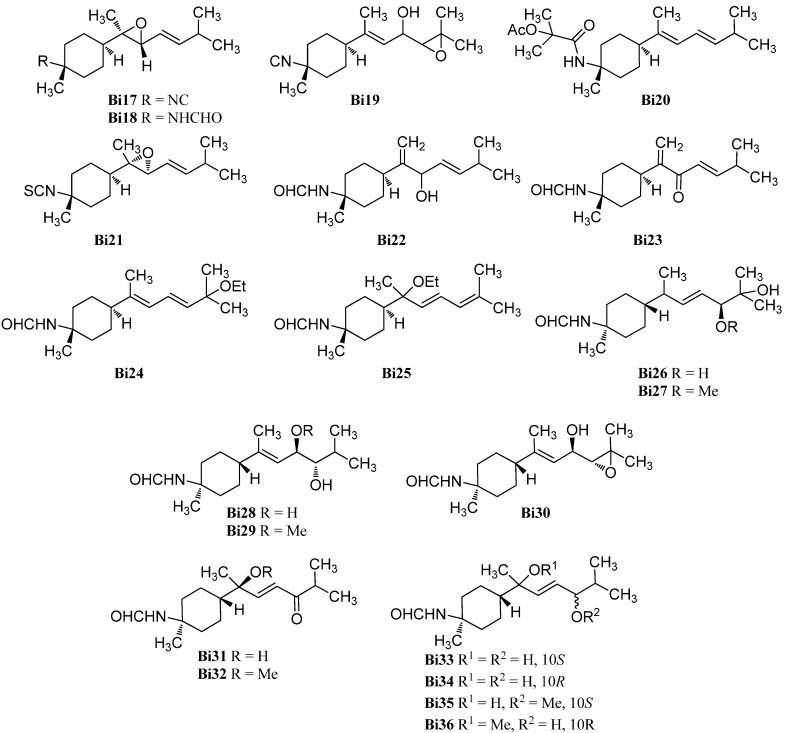
Oxygenated bisabolanes.

In the past 15 years, a considerable number of oxygenated bisabolanes were isolated and characterize, the majority of which being oxygenated formamides (Figure 12).

Sun *et al.* reported the isolation of 3-isocyano-7,8-epoxy-α-bisabolane (**Bi17**) and the corresponding 3-formamido-7,8-epoxy-α-bisabolane (**Bi18**) from the Hainan sponge *Axinyssa* sp. [87]. Recently, axinysaline A (**Bi20**) and axinysaline B (**Bi19**) were found in an unidentified Formosan sponge of the genus *Axinyssa* by Liu *et al.* [88].

Previously, the only oxygenated isothiocyanato compound was 7α,8α-epoxy theonellin isothiocyanate (**Bi21**) which was isolated from *Phycopsis* sp. collected from the Mandapam coast in the Gulf of Mannar, Tamilnadu, India [89].

The first reported oxygenated bisabolanes were 3-formamidobisabolane-14(7),9-dien-8-ol (**Bi22**), 3-formamidobisabolane-14(7),9-dien-8-one (**Bi23**) and 3-formamido-8-methoxybisabolan-9-en-10-ol (**Bi36**). They were all isolated from a Micronesian sponge of the genus *Axinyssa* along with the known 3-formamidotheonellin (**Bi3**) [90].

Two uncommon bisabolanes with an ethoxy group at C-7 or C-11 respectively, 11-ethoxy-3-formamidotheonellin (**Bi24**) and 7-ethoxy-3-formamidobisabolane-8,10-diene (**Bi25**), were isolated from a Hainan sponge *Axinyssa* aff. *variabilis* by Mao *et al.* [91].

In 2014, Cheng *et al.* reported the isolation of ten oxygenated formamidobisabolanes (**Bi26**–**Bi35**) from a Chinese sponge (*Axinyssa* sp.) [92]. Axinyssine C (**Bi26)** has two hydroxy groups at C-10 and C-11 and a double bond in 8-position. Axinyssine D (**Bi27**) has the same structure as **Bi26** but bears a methoxy group at C-10. Axinyssines E and F (**Bi28** and **Bi29**) have the same functional groups as **Bi26/Bi27** but the double bond is located at C-7, one hydroxy group is found at C-10 and the other hydroxy/methoxy group is attached to C-9. However, axinyssine G (**Bi30**) has one hydroxy group at C-9 and an epoxy group at C-10. NOE experiments revealed a 7*E* geometry and the coupling constant *J*_H-9/H-10_ = 8.0 Hz suggests a *threo-*orientation of the two stereocenters in the side chain. The absolute configuration of C-9 was determined by ECD spectra of the Rh_2_(OCOCF_3_)_4_ complex. Axinyssine H (**Bi31**) has a hydroxy group at C-7 and a keto group at C-10. Axinyssine I (**Bi32**) is the methoxylated analogue of Axinyssine H (**Bi31**). The compounds **Bi33**–**Bi36** are the reduced compounds of axinyssine H (**Bi31**) and axinyssine I (**Bi32)**. Axinyssine J (**Bi33**) has two hydroxy groups and the hydroxy-group-bearing C-10 has *S*-configuration. In contrast, axinyssine K (**Bi34**) has *R*-configuration at C-10. Axinyssine L (**Bi35**) carries a methoxy group at C-7 and a hydroxy group at the *S*-configured 10-position. The corresponding 3-formamido-8-methoxybisabolan-9-en-10-ol (**Bi36)** was isolated earlier [90].

#### 2.1.9. Guaianes

**Figure 13:**
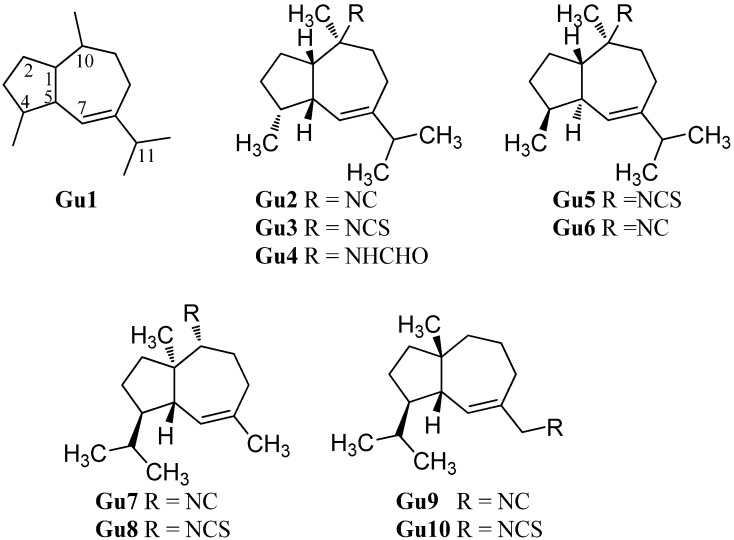
Guaianes.

Guaiane-type sesquiterpenoids have a similar skeleton to the aromadendranes but are devoid of the cyclopropane ring (**Gu1**, Figure 13). Tada *et al.* reported the first isolation of guaiane isonitriloids in 1988. The isonitrile **Gu2**, the isothiocyanate **Gu3** and the formamide **Gu4** were obtained from an unidentified sponge [93]. However, the authors speculated that the formamide **Gu4** was not produced by the sponge but rather was an isolation artifact originating from hydrolysis of **Gu2** during column chromatography [93]. The isothiocyanate **Gu3** was also isolated from the tropical marine sponge *Acanthella cavernosa* wich was followed by a more detailed characterization but the orientation of the isothiocyanato group could not be elucidated [32]. Tada *et al.* deduced the relative configuration of the other congeners based on X-ray crystallography of the isonitrile **Gu2** [93].

(1S*,4S*,5R*,10S*)-10-Isothiocyanatoguaia-6-ene (**Gu5**) was isolated from the sponge *Trachyopsis aplysinoides* collected in Palau [52]. The corresponding isonitrile **Gu6** was isolated in 2004 from the nudibranch *Pthyllidiella pustulosa* from the South Chinese Sea [35].

In 2008, Li *et al.* reported the isolation of 4,5-*epi*-10-isothiocyanatoisodauc-6-ene (**Gu8**) from the Hainan marine sponge *Axinyssa* sp. [84]. In contrast to the other guaianes (**Gu2**–**Gu6**), one of its methyl groups is not located at C-10 but at C-1 and the isopropyl group and the methyl group at C-8 and C-4 are interchanged. The corresponding isonitrile (**Gu7**) was obtained recently by Wright *et al.* from the nudibranch *Phyllidia ocellata* [94].

Another unusual isonitrile/isothiocyanate pair, **Gu9/Gu10**, was isolated in 1987 by Mayol *et al.* from the sponge *Acanthella acuta* [62]. Similar to the isonitrile **Gu7** and the isothiocyanate **Gu8**, the methyl group is located at C-1 and the isopropyl group and the methyl group are interchanged but the nitrogenous functional group is attached to the exocyclic allylic carbon.

#### 2.1.10. Other Sesquiterpenoids

**Figure 14:**
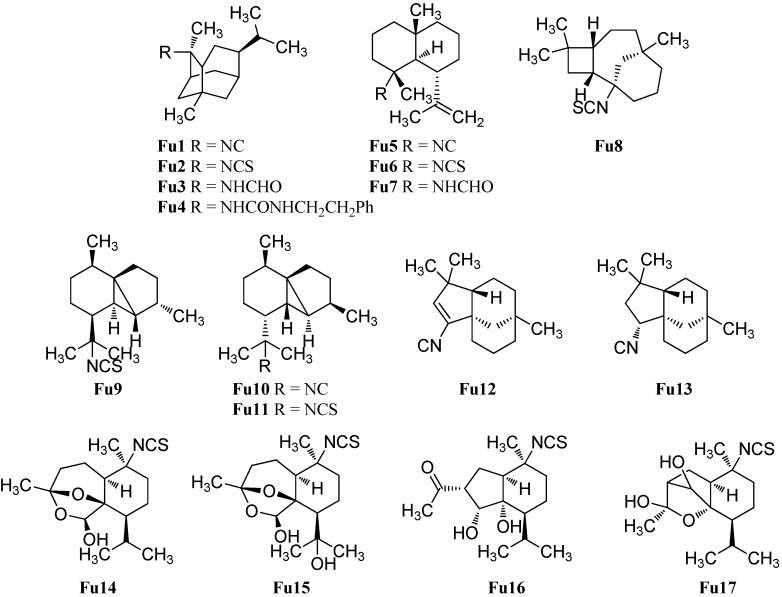
Further sesquiterpenoids.

Apart from the above-mentioned subclasses, there is a small number of marine isonitriloids with uncommon sesquiterpene-based skeletons (Figure 14). He *et al.* reported in 1989 the isolation of 2-isothiocyanatotrachyopsane (**Fu2**) with a novel “trachyopsane” skeleton, which is related to the pupukeanane skeleton, from the sponge *Trachyopsis aplysinoides* [52]. The corresponding 2-isocyanotrachyopsane (**Fu1**) was obtained seven years later by Okino *et al.* from four nudibranchs of the *Phyllidiidae* family [42]. 2-Isocyanotrachyopsane (**Fu1**, IC_50_ = 0.33 µg/mL) exhibited potent antifouling activity against larvae of the acorn barnacle *Balanus amphitrite* [42]. 2-(Formylamino)trachyopsane (**Fu3**) and *N*-phenethyl-*N*′-2-trachyopsane (**Fu4**) were isolated from the Palauan sponge *Axinyssa aplysinoides* [95].

The gorgonane skeleton is very similar to the eudesmane skeleton but the isopropyl group is located at C-6 instead of at C-7. Kassuehlke *et al.* reported the isolation of 4α*-*isocyanogorgon-11-ene (**Fu5**) and 4α*-*formamidogorgon-11-ene (**Fu7**) from the Philippine nudibranch *Phyllidia varicosa* along with 4α-isothiocyanatogorgon-11-ene (**Fu6**) found in the nudibranch *Phyllidia pustulosa* [86].

Recently, White *et al.* isolated (−)-(1*S*,2*R*,5*R*,8*R*)-1-isothiocyanato*epi*caryolane (**Fu8**), the first compound with a caryolane skeleton containing a nitrogenous functional group, from the nudibranch *Phyllidia ocellata* [94].

Cubebane sesquiterpenoids possess a very similar skeleton to the cadinanes but they have an additional bond between C-1 and C-5. The first cubebane type sesquiterpenoid, (1S*,2R*,5S*,6S*,7R*,8S*)-13-isothiocyanatocubebane (**Fu9**), was isolated by He *et al.* from the sponge *Axinyssa aplysinoides* [69]. The relative configuration was defined by NOEDS experiments. The C-2 epimer (1S*,2S*,5S*,6S*,7R*,8S*)-13-isothiocyanatocubebane (**Fu11**) was isolated later by Mitome *et al.* from the Okinawan sponge *Stylissa* sp. [50]. The corresponding (1S*,2S*,5S*,6S*,7R*,8S*)-13-isocyanocubebane (**Fu10**) was obtained in 2015 by White *et al.* from the nudibranch *Phyllidia ocellata* [94].

The same group also isolated (−)-(1*S*,5*S*,8*R*)-2-isocyanoclovene (**Fu12**) and the saturated (−)-(1*S*,2*R*,5*S*,8*R*)-2-isocyanoclovane (**Fu13**) from the nudibranch *Phyllidia ocellata* [94]. The (1*S*,5*S*,8*R)*-configuration of the isonitrile **Fu12** was determined by X-ray crystallography of the corresponding formamide, which could be obtained by treatment of isonitrile **Fu12** with glacial acetic acid. The new stereocenter in the isonitrile **Fu13** was identified by NOESY experiments. The authors propose that the cycloclovane skeleton is formed from the epicaryolane skeleton by an 1,2-alkyl shift [94].

In 2008 Sorek *et al.* reported the isolation of five new marine isothiocyanates (Axiplyns A–E) from the sponge *Axinyssa aplysinoides* collected at Misali Island, Tanzania [54]. Axiplyn A (**Fu14**) and B (**Fu15**) contain a unprecedented ring system, 6,8-dioxabicyclo[3.2.1]octane. However, Axiplyn D (**Fu16**) has a bridged hydroindane skeleton and Axiplyn E (**Fu17**) has a 2-oxabicyclo[2.2.1]heptane core. Axiplyn C (**Ca34**) is a cadinane type sesquiterpenoid and was already described in Section 2.1.3. Axiplyns A (**Fu16**, LD_50_ = 1.6 µg/mL), B (**Fu17**, LD_50_ = 1.5 µg/mL) and C (**Ca33**, LD_50_ = 1.8 µg/mL) displayed toxic activities to brine shrimp larvae (*Artemia salina*) [54].

### 2.2. Diterpenoids

Beside the cyclic sesquiterpenoid compounds already presented in this review, a large fraction of marine isonitriles and isothiocyanates were found to have diterpenoid backbones. They can be categorized into three classes: Acyclic diterpenes, kalihinanes, and amphilectanes.

Until today, six acylic compounds, 69 kalihinanes and 50 amphilectanes have been isolated.

#### 2.2.1. Acyclic

**Figure 15:**
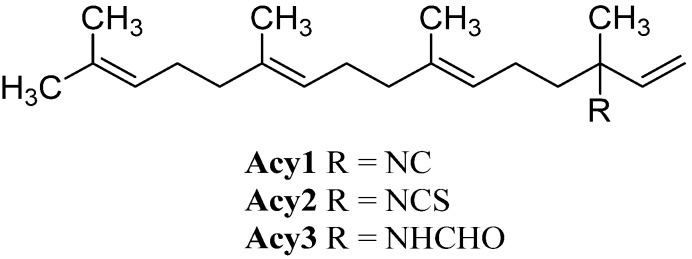
Acyclic diterpenoids.

The smallest class, the acyclic diterpenoids, is represented by a isonitriloid triad consisting of isocyanate **Acy1**, isothiocyanate **Acy2** and formamide **Acy3**, which were the only representatives found until 2006 (Figure 15). The triad was found in 1974 by Burreson *et al.* along with cyclic sesquiterpenes in an unidentified *Halichondria* species [39,40].

**Figure 16:**
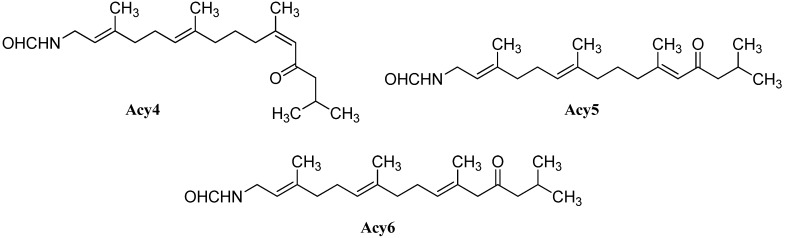
Malonganenones.

Further open-chain terpenoid formamides were isolated from sea fans. Malonganenone C (**Acy4**), a diterpenoid formamide showing moderate cytotoxic activity against esophageal cancer cell lines, was found in extracts of the gorgonian *Leptogorgia gilchristi* collected near Ponto Malongane, Mozambique by Keyzers in 2006 (Figure 16) [96]. It was also found in another sea fan, *Euplexaura nuttingi* (Uvinage, Pemba Island, Tanzania) in 2007 by Sorek *et al.* who were able to isolate Malonganenone C along with its Δ^11,12^-(*E*) double bond isomer malonganenone H (**Acy5**) [97]. Examination of *Euplexaura robusta* collected near Weizhou Island (Guangxi Province, China) in 2012 by Zhang *et al.* revealed the Δ^10,11^-double bond isomer Malonganenone K (**Acy6**) [98]. Despite the occurrence of these three new members this class of acyclic compounds still appears as an exception among the other diterpenoid isonitriles which are usually cyclic.

#### 2.2.2. Kalihinanes

Common to all kalihinanes is the decalin ring system which, apart from some exceptions (cavernenes) which will be discussed later on, either bears a tetrahydofuran, a tetrahydropyran or a dihydropyran moiety linked to C-7. All three rings can bear a large variety of functional groups including isocyano-, isothiocyanato- and hydroxy groups or chlorine. The kalihinanes can be further classified into kalihinols, kalihinenes, kalihipyranes and cavernenes. Most kalihinanes were found in *A. cavernosa* whereas also a few examples exist that were isolated from the sponge *Phakellia pulcherrima.*

##### Kalihinols

**Figure 17:**
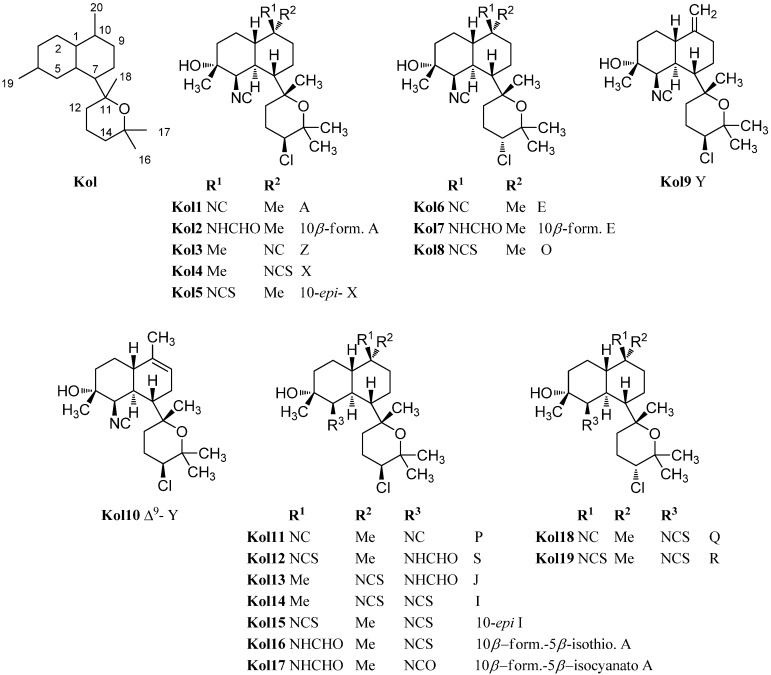
Tetrahydropyran kalihinols.

Characteristic for the kalihinols is the tertiary alcohol function at C-4 of a *trans*-decalin system being part of a bifloran skeleton (Figure 17). They can be further subdevided into two groups according to their substitution with a tetrahydropyran or a tetrahydrofuran moiety at C-7. The first eleven kalihinols were found by the Scheuer group of Hawaii in 1987 in two sponges of *Acanthella* sp. (later indentified as *A. cavernosa*) [99] and were published in a series of communications [100,101,102]. The kalihinols A–H were isolated from an *Acanthella* sp. collected at Guam, whereas examination of a Fijian *Acanthella* species revealed kalihinols X (**Kol4**), Y (**Kol9**) and Z (**Kol3**). Kalihinols A (**Kol1**), E (**Kol6**), X (**Kol4**), Y (**Kol9**) and Z (**Kol3**) are all linked at C-7 to a C-14 chlorinated tetrahydropyran moiety and bear an isonitrile function at C-5. Kalihinol E (**Kol6**) represents the C-14-epimer of kalihinol A (**Kol1**) just having another orientation of the chlorine substituent. The formamide derivatives of A and E, 10β-formamido kalihinol A (**Kol2**) and 10β-formamido kalihinol E (**Kol7**) were isolated in 1996 by Hirota *et al.* from *A. cavernosa* while the isothiocyanato derivative, found in the same producer by Xu *et al.* in 2004, was named kalihinol O (**Kol8**) [20]. Kalihinol Z (**Kol3**) on the other hand represents the C-10-epimer of kalihinol A (**Kol1**), whereas kalihinol X (**Kol4**) is the 10-isothiocyanato derivative of kalihinol Z (**Kol3**). Further investigation of *A. cavernosa* by Sun *et al.* in 2009 revealed a kalihinol with the opposite stereochemistry at C-10, 10-*epi*-kalihinol X (**Kol5**) [103]. Unlike the other tetrahydropyran kalihinols, kalihinol Y (**Kol9**) bears an exocyclic methylene group at C-10. In 1998, Wolf was able to isolate along with kalihinol Y its double bond isomer Δ^9^-kalihinol Y (**Kol10**) from the sponge *Phakellia pulcherrima*, which was the first time that kalihinanes were found in sponge other than *A. cavernosa* [104]. Independently from Wolf, Miyaoka also discovered the same kalihinol in *A. cavernosa* in the same year [105]. In antihelmintic screenings, kalihinol Y (**Kol9**) displayed extremely high activity against the rodent-pathogenic roundworm *Nippostongylus brasiliensis*. The kalihinols A (**Kol2**), X (**Kol4**) and Z (**Kol3**) also showed high activity [106].

The first time that kalihinols with other functional groups than the isonitrile group at C-5 were found in 1991 when Alvi was able to isolate kalihinols I (**Kol14**) and J (**Kol13**) from *A. cavernosa*, which are the 5-formamide and the 5-isothiocyanato congeners of kalihinol X (**Kol4**) respectively [49]. 10-*epi*-Kalihinol I (**Kol15**) was isolated by Miyaoka, while Hirota *et al.* were able to identify the C-5 isocyanato- and isothiocyanato-derivatives 10β-formamido-5β-isothiocyanato-kalihinol A (**Kol16**) and 10β-formamido-5β-isocyanato-kalihinol A (**Kol17**) [20,105]. The isocyanato derivative of kalihinol A (**Kol1**) was found in 2012 by Xu *et al.* and was named kalihinol P (**Kol11**) as well as kalihinol S (**Kol12**), representing the C-5 formamide derivative of 10-*epi*-kalihinol X (**Kol5**) [107]. Further investigations of the same sample revealed kalihinols Q (**Kol18**) and R (**Kol19**), which are the C-14 epimers of **Kol11** and **Kol15** respectively [107].

**Figure 18:**
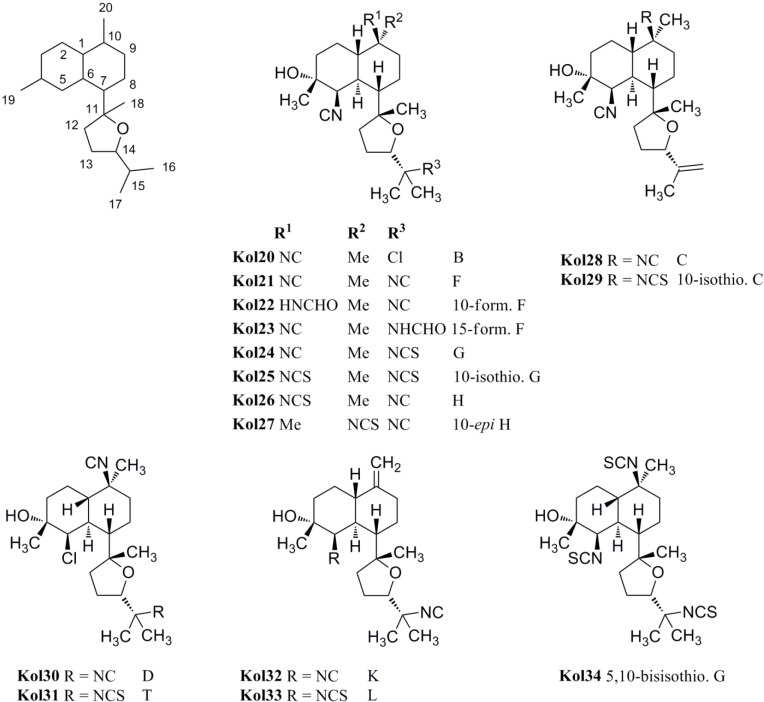
Tetrahydrofuran-substituted kalihinols.

The first representatives of the tetrahydrofuran subclass of kalihinols were kalihinols B (**Kol20**), C (**Kol28**), D (**Kol30**), F (**Kol21**), G (**Kol24**) and H (**Kol26**) discovered by the Scheuer group in the 1980s (Figure 18) [101,102]. The stereochemical and functional variations occur at C-5 and C-10 as in the tetrahydropyran-type kalihinols. Additionally, the tetrahydrofuran moiety can be functionalized at C-15 with isonitrile, isothiocyanate, formamide or chlorine. Examples bearing an isopropenyl side chain are also known.

Kalihinol F (**Kol21**), a representative of the tetrahydrofuran-type kalihinols, is substituted with three isonitrile groups at C-5, C-10 and C-15. It was shown to be a topoisomerase I inhibitor in *Asterina pectinifera*, inhibiting the chromosome separation in fertilized starfish eggs [108] and to have antimicrobial activity against *Bacillus subtilis*, *Staphylococcus aureus* and *Candida albicans* [101]. Kalihinol B (**Kol20**) differs from Kalihinol F (**Kol21**) just in the functional group at C-15, which is a chlorine instead of an isocyano group. In 2004, Bugni was able to isolate the formamide derivatives 10-formamido kalihinol F (**Kol22**) and 15-formamido kalihinol F (**Kol23**) from two Philippine specimens of *A. cavernosa* [109]. The C-15 isothiocyanate derivative of kalihinols B and F was named kalihinol G (**Kol24**), while kalihinol H (**Kol26**) represents the C-10-isothiocyanato derivative of kalihinol F [102].

The first member of the class bearing an isopropenyl side chain was kalihinol C (**Kol28**), which is functionalized at C-5 and C-10 with two isonitrile groups. Kalihinol D (**Kol30**) represents the 5-Cl-derivative of kalihinol F (**Kol21**)[102]. Its C-15-isothiocyanato derivative, kalihinol T (**Kol31**), was isolated by Xu in 2012 from *A. cavernosa* [107].

In 1998, the Miyaoka group was able to isolate the tris-isothiocyanato compound 5,10-bisisothiocyanatokalihinol G (**Kol34**) [105]. Further tetrahydrofuran-type kalihinols were presented in the same year by Wolf *et al.* who identified 10-isothiocyanato kalihinol G (**Kol25**), 10-*epi*-kalihinol H (**Kol27**), and 10-isothiocyanato kalihinol C (**Kol29**) from a sample of *Phakellia pulcherrima* [104]. From the same sample, the structures of two compounds with a 10-*exo*-methylene group were elucidated, kalihinol K (**Kol32**) bearing an isonitrile substituent at C-5 and its isothiocyanato derivative kalihinol L (**Kol33**), representing the tetrahydrofuran analogues of kalihinol Y (**Kol9**) [104].

**Figure 19:**
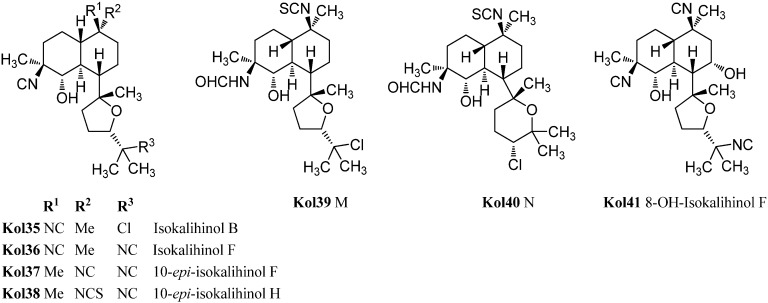
Isokalihinols.

Already in 1988, Omar *et al.* were able to isolate a variant of the kalihinols with swapped positions of the hydroxy and isonitrile groups at C-4 and C-5 (Figure 19) [106]. The stereochemistry of both groups is opposite compared to the normal kalihinols, presumably due to a different opening of the intermediate epoxide in the biosynthetic pathway. The first example of this so-called “isokalihinols” was isokalihinol F (**Kol36**) which, except for C-4 and C-5, showed the same configuration and functionalization as kalihinol F (**Kol21**). 8-OH-Isokalihinol F (**Kol41**), a rather unusual isokalihinol bearing an additional hydroxy group at C-8, was isolated by Clark *et al.* in 2000 from *A. cavernosa* collected at Heron Island (Great Barrier Reef, Australia) [31].

Three further isokalihinols with an NC-function at C-4 are known to date: Isokalihinol B (**Kol35**), being related to kalihinol B (**Kol20**), was found in 1990 by Fusetani in a marine sponge collected in Kuchihoerabu Island of the Satsunan Archipelago (Japan) which was identified as *Acanthella* *klethra* [110]. Rodriguez and Crews revised this assignment afterwards based on voucher samples to be actually *A. cavernosa* [99]. Isokalihinol B (**Kol35**) and kalihinene (**Ken1**) demonstrated besides antifungal activities against *Mortierella romannicus* and *Penicillium chrysogenum* also cytotoxic potency against P388 murine leukemia cells (IC_50_ = 0.8 µg/mL and 1.2 µg/mL), respectively [110].

Many kalihinanes displayed antifouling potency. Kalihinols **Kol1** (EC_50_ = 0.087 µg/mL), **Kol2** (EC_50_ < 0.5 µg/mL), **Kol6** (EC_50_ < 0.5 µg/mL), **Kol7** (EC_50_ = 0.5 µg/mL), **Kol16** (EC_50_ = 0.05 µg/mL) and **Kol17** (EC_50_ = 0.05 µg/mL) and kalihinenes **Ken2** (EC_50_ = 0.095 µg/mL), **Ken14** (EC_50_ = 0.49 µg/mL), **Ken15** (EC_50_ = 0.45 µg/mL) and **Ken16** (EC_50_ = 1.1 µg/mL) were shown to inhibit the attachment and metamorphosis of cyprid larvae of the acorn barnacle *Balanus amphitrite* [20,111].

The other two known isokalihinols are 10-*epi*-isokalihinol F (**Kol37**) and the C-4,5-regioisomer of 10-*epi*-kalihinol H (**Kol27**), 10-*epi*-isokalihinol H (**Kol38**), which were isolated by Faulkner in 1994 from *A. cavernosa* collected in the Seychelles [112].

In 2012, Xu *et al.* reported two novel C-4 formamido representatives of isokalihinols in a South China Sea specimen of *A. cavernosa*, kalihinol M (**Kol39**) bearing an isothiocyanato group at C-10 and a chlorine atom at C-15 and kalihinol N (**Kol40**), the 4-formamido-iso-derivative of kalihinol O (**Kol8)**, representing the first example of isokalihinols of the tetrahydropyran type [107].

##### Kalihinenes

**Figure 20:**
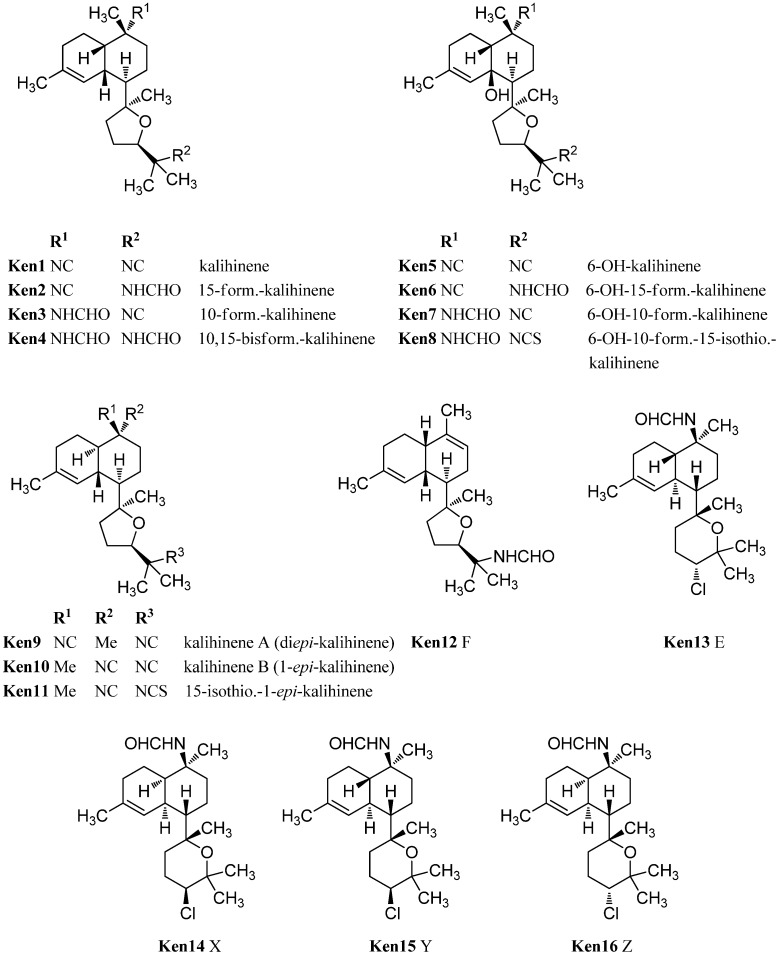
Kalihinenes.

Common to all kalihinenes is the trisubstituted Δ^4^-double bond and a heterocyclic C_8_-substituent at C-7 equal to the kalihinols. However, unlike the kalihinols, which to date exclusively featured *trans*-decalin skeletons, the kalihinenes also exist with *cis*-decalin backbones (Figure 20).

The prototype of this class after which all other members are named, kalihinene (**Ken1**), was found in 1990 by Fusetani in a misassigned *Acanthella klethra* sample (actually *cavernosa*) [110]. Examination of a Fijian sample of *A. cavernosa* by Rodriguez *et al.* resulted in the isolation and identification of 15-formamido-kalihinene (**Ken2**), 10-formamido kalihinene (**Ken3**) and 10,15-bisformamido kalihinene (**Ken4**) [99]. The team additionally proved the persistence of these compounds in sponges kept in an aquaculture for seven months. Together with the three *cis*-formamido kalihinenes, Rodriguez *et al.* were also able to identify four members with a novel 6-hydroxy kalihinene framework. The first representative, 6-hydroxy-kalihinene (**Ken5**), exhibits the same *cis*-decalin backbone and functional groups as kalihinene but is hydroxylated at C-6. The other isolated examples are the formamido- and formamido-isothiocyanato derivatives 6-hydroxy-15-formamido kalihinene (**Ken6**), 6-hydroxy-10-formamidokalihinene (**Ken7**) and 6-hydroxy-10-formamido-15-isothiocyanato kalihinene (**Ken8**) [99].

Another variation of the *cis*-decalin tetrahydrofuran-substituted kalihinenes is kalihinene F (**Ken12**), which was isolated in 2012 by Xu *et al.* and bears a trisubstituted Δ^9^-double bond similar to Δ^9^ kalihinol Y (**Kol10**) [113].

Three kalihinenes with a *trans-*decalin framework were reported in 1994 by Faulkner *et al.* [112]. Besides kalihinene A (also named di*epi*-kalihinene) (**Ken9**) which is, apart from the double bond, identical to kalihinol F (**Kol21**), the group could also isolate kalihinene B (or 1-*epi*-kalihinene) (**Ken10**) and 15-isothiocyanato-1-*epi*-kalihinene (**Ken11)**. In contrast to the twelve isolated members of the tetrahydrofuran-substituted kalihinene series, only four tetrahydropyran derivatives are known to date. The first three examples were the kalihinenes X (**Ken14**), Y (**Ken15**), and Z (**Ken16**) isolated in 1995 by Okino *et al.* from *A. cavernosa* [111]. All of them bear a formamide function at C-10 and a chlorine at C-14. Differences only consist in the stereochemistry at C-1 and C-14. In 2012, the Xu group added the missing fourth diastereomer kalihinene E (**Ken13**) also found in the same producer [113]. Its absolute configuration was elucidated by X-ray crystallography.

Kalihinene (**Ken1**), kalihinol A (**Kol1**), and kalihinol E (**Kol6**) were also found by Manzo *et al.* in 2004 in *Phylidiella pustulosa*, a nudibranch from the South China Sea [35]. The dietary origin of these diterpenoids is strongly supported by several investigations.

**Figure 21:**
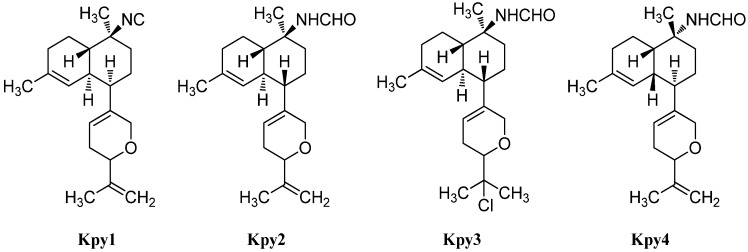
Kalihipyrans.

Besides the tetrahydropyran and tetrahydrofuran series of kalihinenes, a third group of kalihinenes with a different pendant C_8_-unit exists. The first member of this class, kalihipyran (**Kpy1**), was discovered by Faulkner *et al.* in 1994 in *A. cavernosa* (Figure 21) [112]. It shows a kalihinene type *trans*-decalin system with an isonitrile function at C-10 and is connected at C-7 to a dihydropyran with isopropenyl side chain. Its C-10 formamide derivative kalihipyran A (**Kpy2**) was isolated in 2012 by Xu *et al.* along with the *cis*-decalin derivative kalihipyran C (**Kpy4**) [113]. Kalihipyran B (**Kpy3**) isolated by Fusetani, again features a *trans-*decalin backbone, but contains a chlorine in the side chain [114]. The configuration at C-14 remains unknown.

##### Cavernenes and Other Intermediates

**Figure 22:**
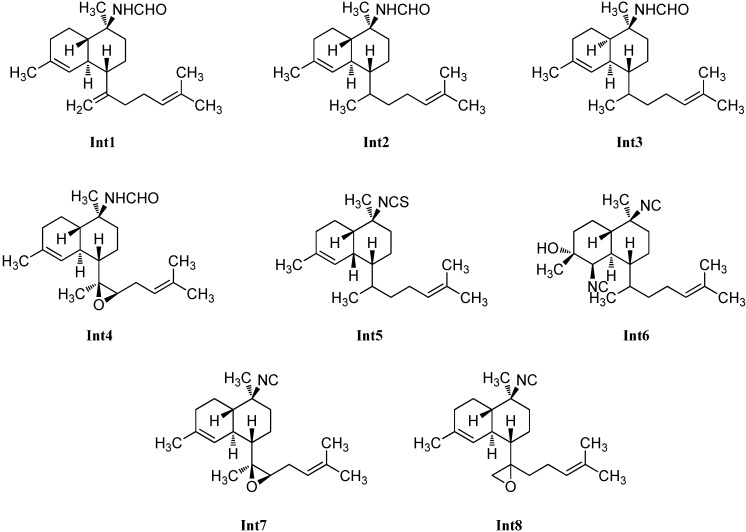
Intermediates.

Over the years, a couple of potential intermediates in the biosynthetic pathway of kalihinanes have been identified (Figure 22). In 1996, the first example of a new class of kalihinane diterpenes with an open-chained substituent at C-7 and a kalihinene *cis*-decalin framework was discovered in *Cymbastella hooperi* by König *et al.* who determined the relative configuration to correspond to that of (1S*,6R*,7R*,10S*,11R*)-10-isothiocyanatobiflora-1,14-diene (**Int5**) ([α]_D_ = +45° (CHCl_3_, c = 0.26)) [115].

Already in 1992, a kalihinene derivative with an open isoprenoid side chain was isolated by Sharma *et al.* from a sample of *Adocia* sp. showing the same 2D structure but a different optical rotation ([α]_D_ = +97° (CHCl_3_, c = 0.085)) compared to König’s compound [116]. Its relative configuration was not elucidated.

Examination of an *A. cavernosa* sponge, collected at Heron Island (Great Barrier Reef, Australia) by Clark *et al.* in 2000 resulted in the isolation of two oxirane-derivatives, 11,12-epoxy-10-isocyano-4,14-bifloradiene (**Int7**) possessing a trisubstituted epoxide in the side chain and a *trans*-decalin framework, and 11,18-epoxy-10-isocyano-4,14-bifloradiene (**Int8**) featuring a terminal epoxide group. The authors suggest the latter one to be a precursor to the kalihipyran ring system [31].

From the same sponge, four further members of this class of intermediates were reported by Xu *et al.* in 2012, which all bear a formamide group at C-10 and differ just in the nature of the side chain linked to C-7 and the *cis*- or *trans*-configuration of the decalin moiety. Cavernene A (**Int1)**, a *trans*-decalin, is connected to an isoprenoid unit. In contrast to cavernene A (**Int1**), cavernene B (**Int2**) shows a monoolefinic isoprenoid side chain, while cavernene C (**Int3**) is identical to cavernene B (**Int2**) apart from the *cis*-decalin moiety. Cavernene D (**Int4**) possesses a trisubstituted epoxide in the side chain and the same *trans*-decalin unit as cavernenes A and B and represents the C-10-formamido derivative of **Int7** [113].

The missing link in the kalihinol synthesis was discovered by Wolf *et al.* in 1998 in a sample of *Phylidiella pulcherrima* and was named pulcherrimol (**Int6**) [104]. In contrast to the other intermediates, it shows the decalin framework of kalihinol A, which makes its role as an intermediate in the biosynthesis of kalihinols plausible.

For the proposed role of the intermediates in the kalihinane synthesis see the Chapter 3 on biosynthesis.

#### 2.2.3. Amphilectanes

**Figure 23:**
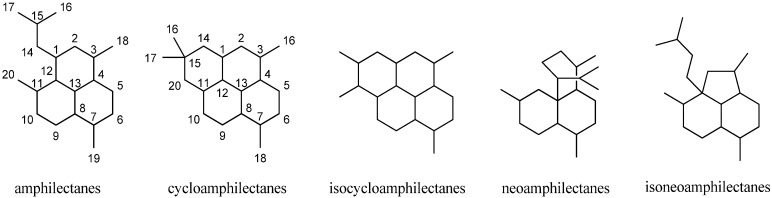
Amphilectane frameworks.

Besides the diterpenoid class of highly substituted decalins, the kalihinanes, which are found almost exclusively in *A. cavernosa* and apart from the small group of acyclic diterpenes, a third class of naturally occurring diterpenoid isonitriles with tricyclic or tetracyclic structures is known. These so-called amphilectanes are predominantly found in *Amphimedon* sp., *Hymenacidon amphilecta* and *Halichondria* sponges and can furthermore be subdivided based on their carbon frameworks into amphilectanes, cycloamphilectanes, isocycloamphilectanes, neoamphilectanes and isoneoamphilectanes (Figure 23). Recently the assignment of the species *Hymeniacidon amphilecta* has been revised to *Pseudoaxinella amphilecta* and will be referred to in the latter way hereinafter [117].

##### Amphilectenes

**Figure 24:**
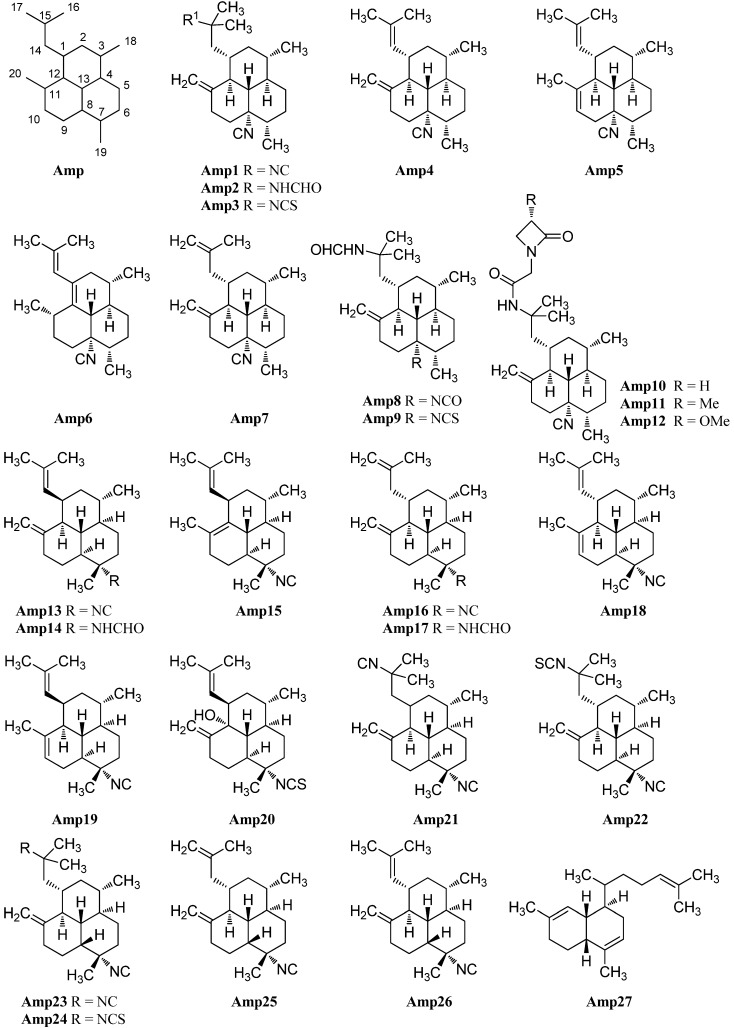
Amphilectenes.

Examination of the Caribbean sponge *Pseudoaxinella amphilecta* by Wratten *et al.* in 1978 revealed a diterpenoid diisocyanide with a novel tricyclic carbon skeleton, 8,15-diisocyano-11(20)-amphilectene/((−)-DINCA) (**Amp1**) which became the lead substance of the new class of amphilectenes (Figure 24). It was shown later by Ciavatta *et al.* to reduce the proliferation of T and B lymphocytes [118]. In the same source, the goup around Wratten was also able to isolate its 15-formamide derivative **Amp2** [119].

The corresponding 15-thiocyanato derivative **Amp3** was isolated in 2005 from the unidentified Caribbean sponge *Cribochalina* sp. by Ciavatta *et al.*, who also were able to assign the absolute configuration of several amphilectenes based on an investigation of **Amp1** by a modification of Mosher’s method in 2005, revising a misassignment of the absolute configuration published in 1999 by the same group [118,120].

Further investigations of a Palauan sponge *Halichondria* sp. by Molinski *et al.* in 1987 resulted in the isolation of 8-isocyano-10,14-amphilectadiene (**Amp5**) bearing an isobutenyl side chain [121]. In 2004, Mitome *et al.* were able to find two double bond isomers (1S*,3S*,4R*,7S*,8S*,12S*,13S*)-8-isocyanato-amphilecta-11(20),14-diene (**Amp4**) and (3S*,4R*,7S*,8S*,11S*,13S*)-8-isocyanoamphilecta-1(12),14-diene (**Amp6**) in an unidentified Okinawan sponge *Stylissa* sp. taken from the coral reef of Iriomote Island (Japan) [50].

Another double bond isomer was identified in 2009 by Wattanapiromsakul *et al.* who were able to isolate 8-isocyanoamphilecta-11(20),15-diene (**Amp7**) from *Ciocalapata* sp. (family *Halichondriidae*), collected in Koh-Tao in the Surat-Thani province of Thailand [122].

Examination of the sponge *Stylissa* cf. massa, collected also in Koh-Tao (Thailand) by Chanthathamrongsiri in 2012 revealed two C-5 derivatives of **Amp2**, 8-isocyanato-15-formamidoamphilect-11(20)-ene (**Amp8**) and 8-isothiocyanato-15-formamidoamphilect-11(20)-ene (**Amp9**) [123].

The isolation of three unprecedented β-lactam amphilectenes was reported in 2010 and 2015 by Avilés *et al.* from a Puerto Rican *Hymeniacidon* sp. (Demospongiae) collected in Mona Island [124,125]. The absolute configuration of monamphilectine A (**Amp10**), B (**Amp11**) and C (**Amp12**) was assigned by semisynthesis starting from 8,15-diisocyano-11(20)-amphilectene/((−)-DINCA) (**Amp1**). Monamphilectines B and C bear a methyl and a methoxy group in α-position of the β-lactam and represent the first α-substituted β-lactams of marine origin.

Monoamphilectine A (**Amp10**) and **Amp1** exhibited activity against *Mycobacterium tuberculosis* H_37_Rv with MIC values of 15.3 µg/mL and 3.2 µg/mL, respectively [124].

7-Isocyano-11(20),14-*epi*amphilectadien (**Amp13**) or (1R*,3S*,4R*,7S*,8S*,12S*,13S*)-7-isocyanoamphilecta-11(20),14-diene, isolated in 1980 by Kazlauskas from an *Adocia* species, was the first member of a series of amphilectanes bearing a 7-isonitrile substituent instead of a substituent at C-8 [126]. Its corresponding formamide (1R*,3S*,4R*,7S*,8S*,12S*,13S*)-7-formamidoamphilecta-11(20),14-diene (**Amp14**) was found in 2009 by Wright *et al.* in the tropical marine sponge *Cymbastela hooperi* (Kelso Reef, Queensland, Australia) [127]. Already in 1996, an examination of the same sponge by König *et al.* had revealed among others the presence of five new 7-substituted amphilectenes, the Δ^11,12^-double bond isomer of **Amp13** (1R*,3S*,4R*,7S*,8S*,13R*)-7-isocyanoamphilecta-11,14-diene (**Amp15**), (1S*,3S*,4R*,7S*,8S*,12S*,13S*)-7-isocyano-amphilecta-10,14-diene (**Amp18**), the C-15 isothiocyanato *exo*-methylene derivative (1S*,3S*,4R*,7S*,8S*,12S*,13S*)-7-isocyano-15-isothiocyanatoamphilecta-11(20)-ene (**Amp22**), and (1R*,3S*,4R*,7S*,8S*,12R*,13R*)-12-hydroxy-7-isothiocyanato-amphilecta-11(20),14-diene (**Amp20**), an unprecedented natural product which still represents the only example of an amphilectane containing an oxygen-based functional group [115].

The (1S*,3S*,4R*,7S*,8S*,12S*,13S*)-7-isocyanoamphilecta-11(20),15-ene (**Amp16**) also isolated by König, is probably identical with the so-called 7-isocyano-11(20),15-*epi*amphilectadiene that was described by Kazlauskas *et al.* in 1980 with an undetermined configuration at C-1 [115,126]. From the same sample, Kazlauskas was able to isolate 7,15-diisocyano-11(20)-*epi*amphilectene (**Amp21**) again with an uncertain stereochemistry at C-11.

Further investigation of a sample of *Stylissa* sp. by Mitome *et al.* revealed the C-1-epimer of **Amp18** (1R*,3S*,4R*,7S*,8S*,12S*,13S*)-7-isocyanoamphilecta-10,14-diene (**Amp19**), while examination of *Cymbastela hooperi* by Wright *et al.* in 2009 resulted in the discovery of the formamide-derivative of **Amp16**, (1S*,3S*,4R*,7S*,8S*,12S*,13S*)-7-formamidoamphilecta-11(20),15-diene (**Amp17**) [50,127].

X-ray structural analysis of amphilectene metabolites from an unidentified *Cribochalina* species by Ciavatta *et al.* in 2005 led to the discovery of three members of the amphilectane class with a rather unusual *cis*-ring junction between C-8 and C-13, 7,15-diisocyano-11(20)-amphilectene (**Amp23**), its C15 isothiocyanato derivative 7-isocyano-15-isothiocyanato-11(20)-amphilectene (**Amp24**), as well as the isobutenyl derivative (1*S*,3*S*,4*R*,7*S*,8*R*,12*S*,13*S*)-7-isocyanoamphilecta-11(20),15-diene (**Amp25**) which is the C-8-epimer of **Amp16 [120]**.

In 2011, Lamoral-Theys *et al.* were able to reisolate compounds **Amp1**, **Amp23** and **Amp25** from the Caribbean sponge *Pseudoaxinella flava* from the Grand Bahamas along with a new representative with *cis*-junction **Amp26**. Amphilectenes **Amp25** and **Amp26** showed cytotoxic effects against the apoptosis-sensitive human PC3 prostate cancer cell line whereas **Amp1** and **Amp23** displayed cytostatic effects [128].

A putative precursor **Amp27** of the amphilectene compounds with a biflora-4,9,15-triene skeleton was isolated by Hirota in 1996 [20]. The absolute configuration and a plausible biosynthetic pathway to the amphilectanes were reported in 2005 by Ciavatta [120].

##### Cycloamphilectenes

**Figure 25:**
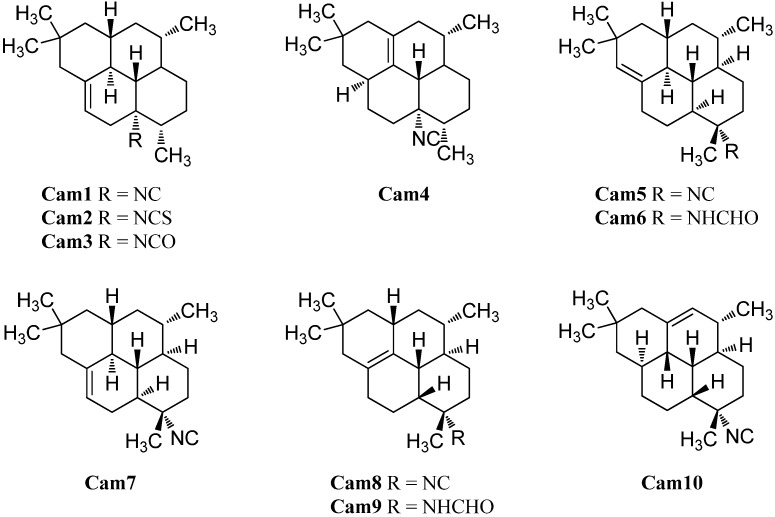
Cycloamphilectenes.

The first representative of the cycloamphilectanes (Figure 25), 8-isocyano-10-cycloamphilectene (**Cam1**) was isolated in 1980 by Kazlauskas *et al.* from an *Adocia* specimen [126]. The group also was able to detect traces of a second cycloamphilectane but due to the limited amount, the structure was initially misassigned. The correct relative configuration was reported in 1987 by Faulkner *et al.* who proved the compound to be (3S*,4R*,7S*,8S*,11S*,13R*)-8-isocyano-1(12)-cycloamphilectene (**Cam4**) after reisolation from a Palauan *Halichondria* specimen [121].

In 2004, investigation of the Okinawan sponge *Stylissa* sp. by Mitome *et al.* resulted in the isolation of the isocyanato and isothiocyanato derivatives of **Cam1**, 8-isocyanatocycloamphilect-10-ene (**Cam3**) and 8-isothiocyanato-amphilect-10-ene (**Cam2**) [50]. Their absolute configuration was determined by anomalous X-ray dispersion of the corresponding semisynthetic 8-*p*-bromobenzamide derivative to be 1*S*,3*S*,4*R*,7*S*,8*S*,12*S*,13*S*. Cycloamphilectanes **Cam** **2** and **Cam3** and amphilectanes **Amp6** and **Amp18** displayed weak cytotoxicity towards HeLa cells showing IC_50_ values of 88.7 µg/mL, 38.3 µg/mL, 11.2 µg/mL, and 20.0 µg/mL, respectively [50].

In 1996, the group around König was able to identify two 8-isocyano cycloamphilectanes in a sample of *C. hooperi*, the (1S*,3S*,4R*,7S*,8S*,12S*,13S*)-7-isocyanocylcoamphilect-10-ene (**Cam7**) and the (1S*,3S*,4R*,7S*,8S*,12S*,13S*)-7-isocyanocycloamphilect-11(20)-ene (**Cam5**) [115]. The formamide derivative of the latter compound, (1S*,3S*,4R*,7S*,8S*,12S*,13S*)-7-formamidocycloamphilect-11(20)-ene (**Cam6**) was reported by Wright *et al.* in 2009 [127]. As for the amphilectenes, some members of the cycloamphilectenes with the uncommon 8,13-*cis*-junction are also known. The first two examples, (1S*,3S*,4R*,7S*,8R*,13R*)-7-isocyano-11-cycloamphilectene (**Cam8**) and (3S*,4R*,7S*,8R*,11S*,12R*,13S*)-7-isocyano-1-cycloamphilectene (**Cam10**) were revealed in 1987 by Molinski *et al.* during their investigations of a Palauan *Halichondria* species [121]. In 2002, Ciasullo *et al.* were able to find the (1S*,3S*,4R*,7S*,8R*,13R*)-*N*-formyl-7-amino-11-cycloamphilectene (**Cam9**) in a sample of *Axinella* sp. from Vanuatu [129].

##### Isocycloamphilectanes

**Figure 26:**
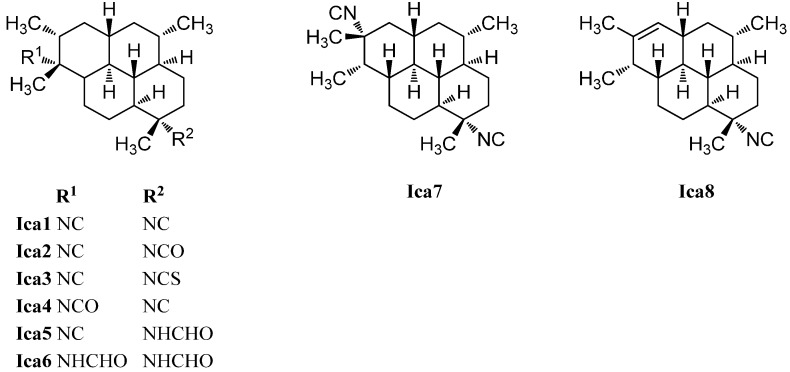
Isocycloamphilectanes.

The first representative of the isocycloamphilectenes (Figure 26), which could be derived from amphilectene or cycloamphilectene precursors by a formal single methyl shift [126], was diisocyanoadociane or (1*S*,3*S*,4*R*,7*S*,8*S*,11*S*,12*S*,13*S*,15*R*,20*R*)-7,20-diisocyanoisocycloamphilectane (**Ica1**), isolated already in 1976 by Baker from a sponge species of the genus *Adocia* (Great Barrier Reef, Townsville, Australia) [130]. Its absolute configuration was determined in 1988 by Fookes via X-ray analysis of its *p*-bromobenzamide derivative and was additionally proven by total synthesis (Corey 1987) [131,132]. The mono- and diformamido derivatives (1*S*,3*S*,4*R*,7*S*,8*S*,11*S*,12*S*,13*S*,15*R*,20*R*)-7-formamido-10-isocyanoisocycloamphilectane (**Ica5**) and (1*S*,3*S*,4*R*,7*S*,8*S*,11*S*,12*S*,13*S*,15*R*,20*R*)-7,20-diformamidoisocycloamphilectane (**Ica6**) were found by Wright in 2009 in a sample of *Cymbastela hooperi* [127]. Examination of the same species thirteen years earlier by König *et al.* resulted in the isolation of the isocyanato compounds (1*S*,3*S*,4*R*,7*S*,8*S*,11*S*,12*S*,13*S*,15*R*,20*R*)-20-isocyano-7-isocyanatoisocycloamphilectane (**Ica2**) and (1*S*,3*S*,4*R*,7*S*,8*S*,11*S*,12*S*,13*S*,15*R*,20*R*)-20-isocyanato-7-isocyanoisocycloamphilectane (**Ica4**) and the isothiocyanato derivative (1*S*,3*S*,4*R*,7*S*,8*S*,11*S*,12*S*,13*S*,15*R*,20*R*)-20-isocyano-7-isothiocyanatoisocycloamphilectane (**Ica3**) [115]. Apart from these, the group was also able to identify the monoolefinic derivative (1S*,3S*,4R*,7S*,8S*,11R*,12R*,13S*,20S*)-7-isocyanoisocycloamphilect-14-ene (**Ica8**).

One example of an isocycloamphilectane with a different substitution pattern at C-15 and C-20 is known, the diisocyano compound 7,15-diisocyanoadociane (**Ica7**), isolated in 1980 by Kazlauskas [126,130].

##### Neoamphilectene

**Figure 27:**
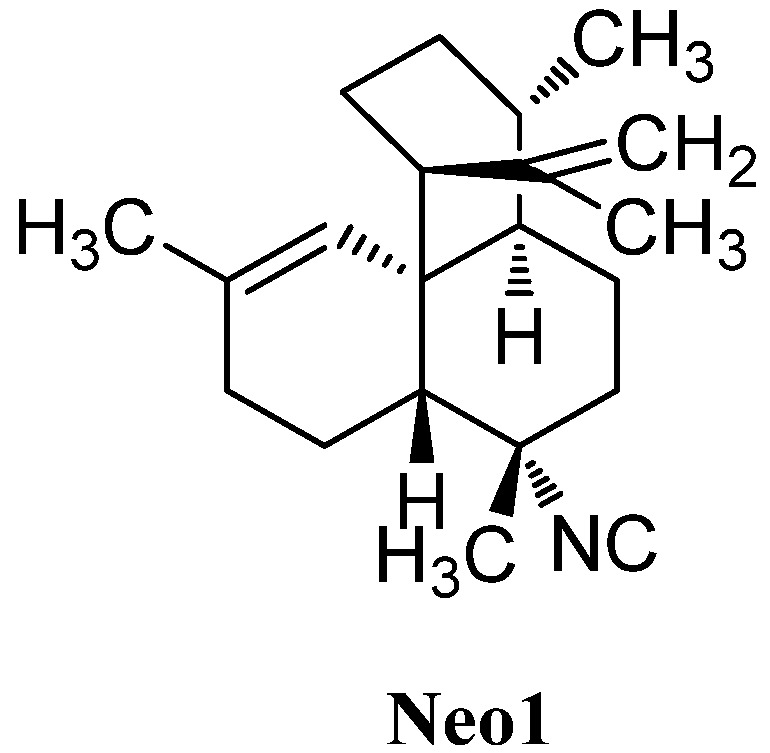
Neopamphilectene.

In 1992, a compound with an unusual amphilectane-framework in which ring C is spirocyclic to the cyclohexene ring was found by Sharma in a sponge of the *Adociae* family, collected off the island of Miyako (Japan, Figure 27). To date 7-isocyanoneoamphilecta-11,15-diene (**Neo1**) remains the only known representative of its class [116].

#### Isoneoamphilectenes

**Figure 28:**
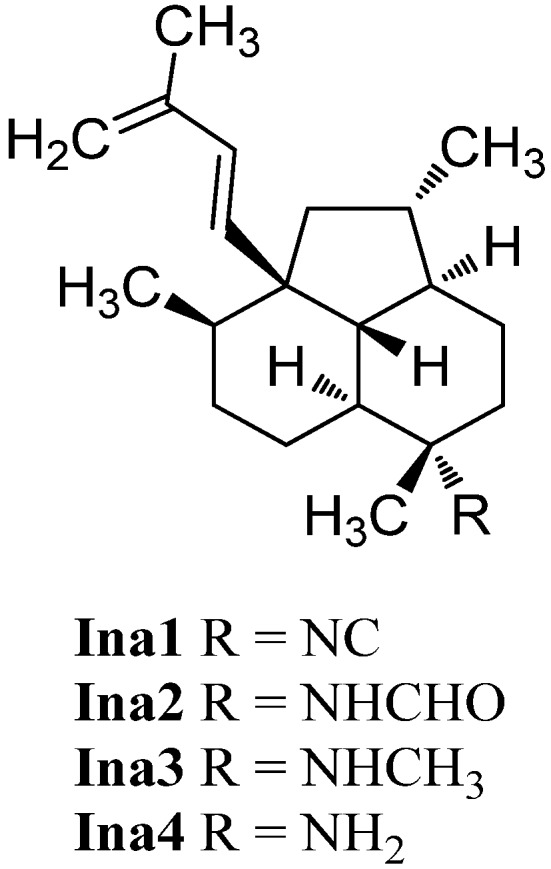
Isoneoamphilectenes.

In 1996, examination of *C. hooperi* by König *et al.* revealed the unique tricyclic diterpene 7-isocyanoneoamphilecta-1(14),15-diene (**Ina1**, Figure 28) [115]. It was not before 2013 that Avilés *et al.* were able to identify two further members of this class, the formamide derivative 7-formamidoisoneoamphilecta-1(14),15-diene (**Ina2**) and the unusual *N*-methyl derivative 7-methylaminoisoneoamphilecta-1(14),15-diene (**Ina3**) in extracts of the Caribbean sponge *Svenzea flava* collected in Great Inagua Island, Bahamas [133]. Avilés *et al.* were able to determine the absolute configuration of these natural products to be (3*S*,4*R*,7*S*,8*S*,11*R*,12*S*,13*R*) by VCD-spectroscopy and DFT-calculations of the semisynthetic NH_2_-derivative **Ina4**.

Isoneoamphilectenes **Ina1**, **2**, **3** and the semisynthetic **Ina4** demonstrated strong growth inhibition potency against *Mycobacterium tuberculosis* H_37_Rv (MIC = 8 µg/mL, 15 µg/mL, 32 µg/mL and 6 µg/mL, respectively) [133].

### 2.3. Carbonimidic Dichlorides

**Figure 29:**
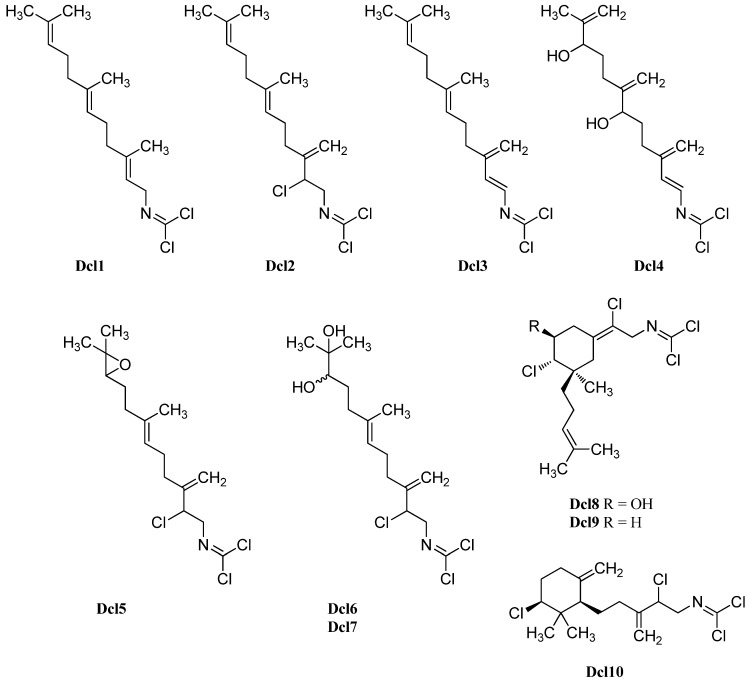
Acyclic and monocyclic carbonimidic dichlorides.

**Figure 30:**
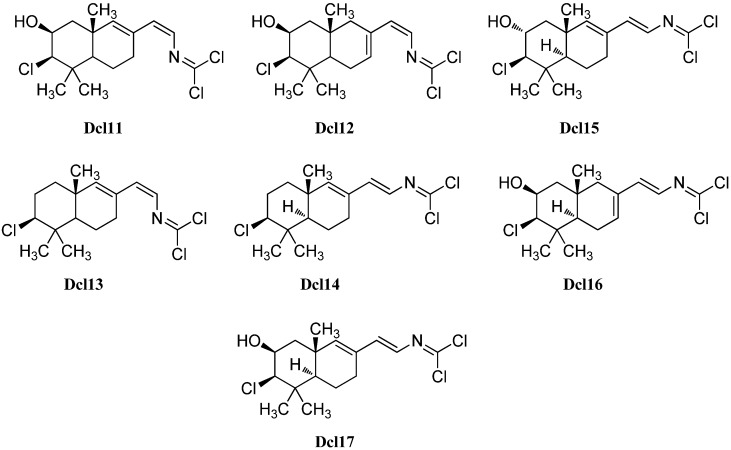
Bicyclic carbonimidic dichlorides.

Carbonimidic dichlorides or dichloroimines represent a rare group of natural products of which to date only 16 representatives are known which are all of marine origin (Figure 29 and Figure 30). They have been isolated from four marine sponges (*Pseudaxinyssa pitys*, *Axinyssa* sp., *Stylotella aurantium* and *Ulosa spongia*) and from the nudibranch *Reticulidia fungia* presumably feeding on these sponges. Carbonimidic dichlorides exhibit a strong IR absorption at ~1650 cm^−1^ and a low intensity ^13^C signal at δ ≈ 127 ppm and can therefore be unequivocally identified.

The first examples of this class of natural products were isolated in 1977 from the Indo-pacific marine sponge *Pseudoaxinyssa pitys* by Wratten, who identified the *trans* linear isoprenoid derivative **Dcl2** containing three chlorines [134]. The substance was isolated again in 1997 by Simpson from *Stylotella aurantium*, a tropical marine sponge collected at Heron Island (Great Barrier Reef, Australia), and was named stylotellane B (**Dcl2**) [135]. The sponge also contained farnesyl isothiocyanate (**Mis1**) and Stylotellane A (**Dcl1**), a similar sesquiterpene without the chlorine substituent at C-2. Both stylotellanes were testet against tumor cell line P-388, however only a weak activity was observed for stylotellane B (**Dcl** **2**).

Investigation of the Australian sponge *Ulosa spongia* collected at Wistari Reef (Great Barrier Reef, Australia) by König *et al.* in 2001 revealed the presence of ulosin A (**Dcl3**) which could be obtained from stylotellane B (**Dcl2**) by formal elimination of HCl and ulosin B (**Dcl4**) bearing three *exo*-methylene groups and two hydroxy groups at C-6 and C-10 [136]. Ulosin A was found independently from König in the same year also by Musman *et al.* in Okinawan *Stylotella aurantium* [137].

Examination of a marine sponge of the genus *Axinyssa* collected off Hachijo-jima Island (Japan) by Hirota *et al.* in 1998 led to the isolation of three new oxygenated sesquiterpenoid carbonimidic dichlorides [53]. Axinyssimide A (**Dcl5**) represents the 10,11-epoxy derivative of stylotellane B while axinyssimides B (**Dcl6**) and C (**Dcl7**) turned out to be the 10,11-dihydroxy derivatives resulting from a ring-opening of the oxirane moiety. Axinyssimides B and C, differing in magnitude and sign of their optical rotation, are diastereomers. All three compounds demonstrated potent antifouling activities against the cypris larvae of the acorn barnacle *Balanus amphitrite* with an IC_50_ value of 1.2 µg/mL for axinyssimide A, 70% inhibition at a concentration of 0.5 µg/mL for axinyssimide B and 90% inhibition at the same concentration for axinyssimide C respectively [53].

Apart from stylotellane B, the investigation of *Pseudoaxynissa pitys* in 1977 by Wratten also revealed a monocyclic sesquiterpene containing the carbonimidic dichloride functionality [134]. This (1R*,5S*,6S*)-6,14-dichloro-5-hydroxy-9,3(14)-(*Z)*-axinyssadien-15yl-carbonimidic dichloride (**Dcl8**) was the first representative of a natural product with an axinyssane framework. In 2004, a second example without hydroxy goup at C-5 (**Dcl9**) was discovered by Brust *et al.* [138].

Another monocyclic carbonimidic dichloride (**Dcl10**) was isolated in 2001 by Musman *et al.* from *Stylotella aurantium* collected from a coral reef of Iriomate Island (Okinawa, Japan) [137]. Both monocyclic carbon skeletons can be derived from Stylotellane B by an enzymatic chlorination including a formal "chloronium ion" initiated cyclization [139].

Further examination of the *Pseudoaxinyssa pitys* saple of Wratten in 1978 also revealed four examples of bicyclic sesquiterpenes with carbonimidic dichloride functionality [140]. Compound **Dcl11**, its Δ^7,8^-double bond isomer **Dcl12** and the non-hydroxylated derivative **Dcl13** all show a *Z*-configurated Δ^14^-double bond in the side chain. The fourth isolated isomer, isoreticulidin B (**Dcl17**) showing a Δ^7,8^-14(*E*)-diene system, was also found in the nudibranch *Reticulidia fungia* collected at Irabu Island (Okinawa) in 1999 by Tanaka *et al.* besides a monocyclic (**Dcl8**) and two bicyclic compounds that were named reticulidins A (**Dcl15**) and B (**Dcl16**) [141]. In 2001, Musman *et al.* were able to determine the absolute configuration of reticulidin A by a modification of Mosher’s method to be 2*R*,3*R*,5*S*,10*S* [137]. The same group also revealed the structure of the non-hydroxylated derivative **Dcl14** of reticulidin A and isoreticulidin B isolated from *Stylotella aurantium*.

**Dcl10** and **Dcl14** exhibited moderate cytotoxicity showing IC_50_ values of 0.1–1 µg/mL against the tumor cell lines P-388, A549, HT29 and MEL28 [137]. Furthermore, reticulidins A (**Dcl15**) and B (**Dcl16**) showed moderate cytotoxic potency against KB cells (IC_50_ 0.41 and 0.42 µg/mL) and L1210 cells (0.59 and 0.11 µg/mL) [141].

### 2.4. Other Marine Isonitriles and Related Compounds (Miscellaneous Structures)

**Figure 31:**
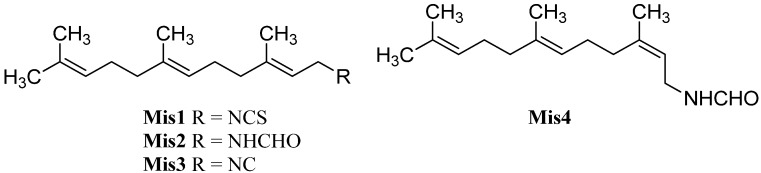
Farnesyls.

Among the isonitriles, isothiocyanates, and formamides of marine origin there are compounds that cannot be categorized in the structural families already presented (Figure 31).

Farnesylisothiocyanate (**Mis1**), an intermediate in the biosynthetic pathway of many sesquiterpene-isothiocyanates, was isolated in 1997 by Simpson from *Stylotella aurantium* [135,140]. Its corresponding formamide **Mis2** and isofarnesyl formamide (**Mis4**) were found in 2008 in *Axinyssa* sp. collected in Sanya, Hainan Province, China by Li [84]. Until now, farnesyl isocyanide (**Mis3**) remains elusive which might be caused by the instability of this compound.

**Figure 32:**
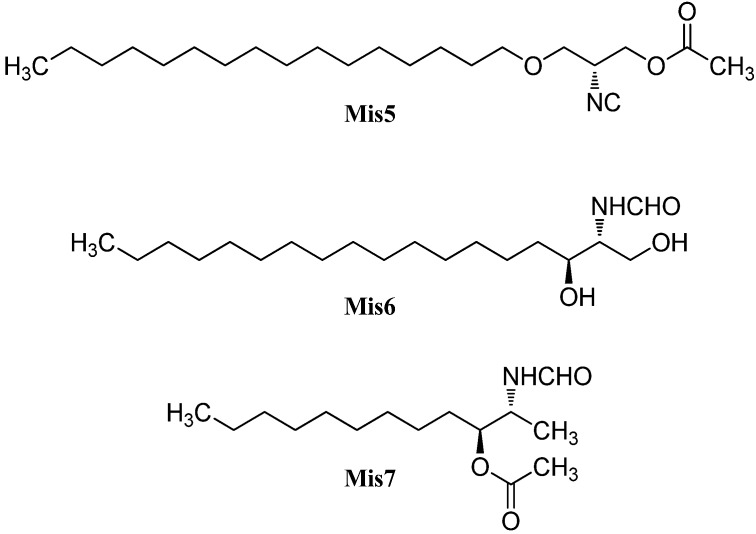
Lipid and amino alcohol derivatives.

Apart from the marine isonitriles and isothiocyanates of terpenoid origin, a few examples with long saturated alkyl chains and structures related to sphingoids and lipids are also known (Figure 32). Investigation of the dorid nudibranch *Actinocyclis papillatus* collected at Wei Zhou Island (South China Sea, China) by Manzo *et al.* in 2011 resulted in the isolation of the unique ether lipid (*R*)-(−)-actisonitrile (**Mis5**) [142]. In 2008, Hung *et al.* reported the isolation of the ceramide (2*R*,3*S*)-2-formamido-1,3-dihydroxy-octadecane (**Mis6**) from the red alga *Gracilaria verrucosa* collected off the coast of Jeju Island, South Korea [143]. A further example, the sphingoid clavaminol L (**Mis7**), was isolated by Aiello *et al.* in 2009 from the Mediterranean ascidian *Clavelina plegraea* found in the Bay of Naples, Italy [144].

**Figure 33:**
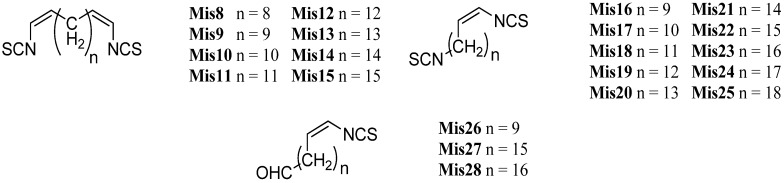
Long-chain isothiocyanates.

In 1987, the investigation of an unidentified Fijian *Pseudoaxinyssa* species by Karuso/Scheuer resulted in the isolation of 21 mono- and diolefinic long-chain isothiocyanates (Figure 33). Eight diolefinic α,ω-bisisothiocyanates (**Mis8**–**15**) were found along with 10 monoolefinic α,ω-bisisothiocyanates (**Mis16**–**25**) and three monoolefinic α-isothiocyanato-ω-formyl derivatives (**Mis26**–**28**) [145].

**Figure 34:**
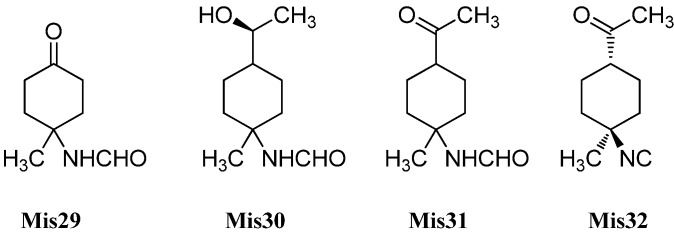
Derivatives with C_7_- and C_9_-carbon skeletons.

Examination of an *Axinyssa* sp. collected in the Andaman Sea (inner coral reef, Thailand) in 2014 by Cheng *et al.* revealed the bisabolene-based formamides axinyssine A (**Mis29**), axinyssine B (**Mis30**) and 1-acetyl-4-formamido-4-methylcyclohexane (**Mis31**, Figure 34) [92]. The corresponding isonitrile of **Mis32** 1-acetyl-4-isocyano-4-methylcyclohexane had already been discovered in the nudibranch *Phyllidia* sp. by Gulavita *et al.* in 1986 [74]. These four compounds could represent catabolic derivatives of the axinyssine sesquiterpenoids presented earlier.

**Figure 35:**
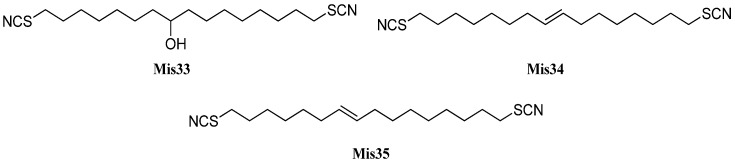
Thiocyanatins.

Despite from the huge variety of marine isothiocyanates just a few examples of structures bearing a thiocyanate functionality are known (Figure 35). Among these is thiocyanatin A (**Mis33**) that was isolated from *Oceanapia* sp. (collected off the northern Rottnest shelf, Australia) in 2001 by Capon *et al.* together with its Δ^8^- and Δ^7^-elimination products thiocyanatins B (**Mis34**) and C (**Mis35**) [146]. Thiocyanatin A was reported to possess *in vitro* nematicidal activities against the barber pole worm *Haemonchus contortus* whereas the non-hydroxylated derivatives thiocyanatins B and C showed no activity at all.

**Figure 36:**
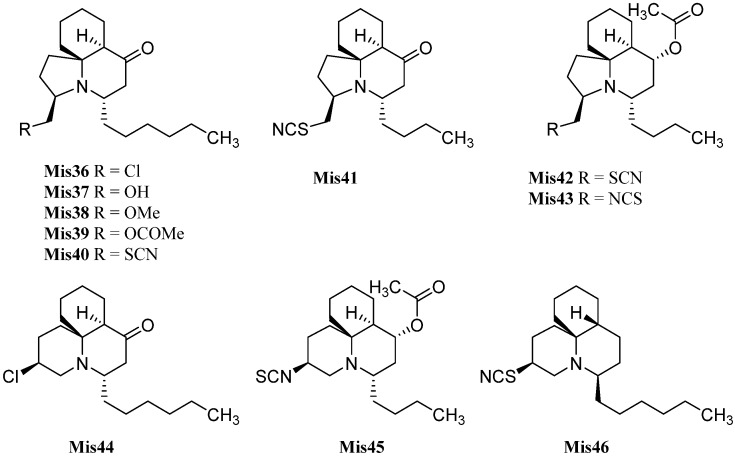
Cylindricins and Fasicularin.

Three other thiocyanates with a unique carbon skeleton were found in the ascidian *Clavelina cylindrica* collected from the Bay of Islands (South Bruny Island, Tasmania) by Li *et al.* in 1994 (Figure 36) [147]. Cylindricines F (**Mis40**) and G (**Mis41**) show the same carbonyl skeleton as cylindricin A (**Mis36**) which was the first natural pyrrolo[2,1-*j*]quinoline to be identified. The isolation of cylindricine H (**Mis30**), the C-4-acetoxy-derivative of cylindricin G, was reported together with its corresponding isothiocyanate cylindricin I (**Mis43**) and the pyrido[2,1-*j*]quinoline isothiocyanate cylindricin J (**Mis45**), which shows the cylindricin B ring system (**Mis44**) [148]. Li also reported the interconvertibility of both ring systems.

In 1997 Patil *et al.* reported the isolation of an thiocyanate derivative with a non-oxygenated pyrido[2,1-*j*]quinolin ring system from the Micronesian ascidian *Nephteis fasicularis*, which was named fasicularin (**Mis46**). It was demonstrated to have a selective activity against a DNA-repair deficient yeast strain and to be cytotoxic against Vero cells (IC_50_ = 14 µg/mL) [149].

**Figure 37:**
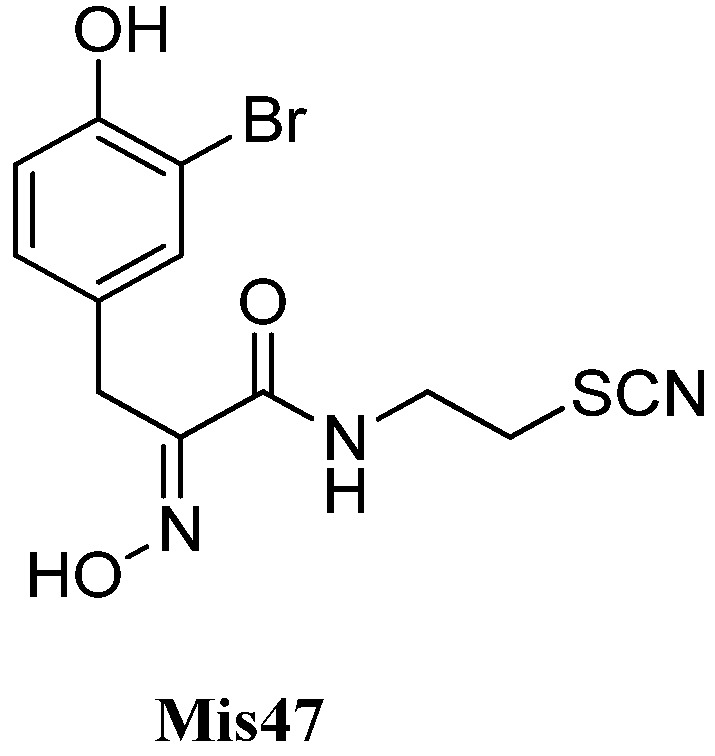
Psammaplin B.

Another thiocyanate derivative was revealed when the investigation of the sponge *Psammaplysilla purpurea* by Jiménez and Crews in 1991 resulted in the isolation of the highly modified bromotyrosine-cysteine derivative psammaplin B (**Mis47**) showing antimicrobial activity against Gram-positive *Staphylococcus* aureus and mild tyrosine kinase inhibition potency (IC_50_ = 2800 µM) (Figure 37) [150].

## 3. Biosynthesis

Two years after the first isolation of a marine isonitrile (**Ax2**) in 1973, Fattorusso *et al.* and Burreson *et al.* observed that the isonitriles are often occurred associated with the corresponding formamido and isothiocyanato analogues. Therefore, they speculated that the formamide function was the precursor for the isonitrile function which itself was the precursor for the isothiocyanate function [5,6].

Iengo *et al.* fed the sponge *Axinella cannabina* with [^14^C]-labeled axamide-1 (**Ax4**) over five days in well aerated sea water. No incorporation of the labeled formamide was observed and therefore the authors assumed that the formamide function is not the precursor for the isonitrile function. Nevertheless the authors questioned their own results because of several factors such as an insufficient incorporation rate [151].

Hagadone *et al.* performed experiments with the sponge *Hymeniacidon* sp. in its natural habitat. They synthesized [^13^C]-labeled 2-formamidopupukeanane (**Pu9**) and 2-isothiocyanatopupukeanane and fed the sponges with these compounds. However, no [^13^C]-labeled 2-isocyanopupukeanane (**Pu7**) was isolated after usual workup. Thereby neither the formamide function nor the isothiocyanato function appear to be the precursor for the isonitrile. Additionally, they observed, that [^13^C]-formate is not a component in the biosynthesis of marine isonitriles. Finally, the sponge *Hymeniacidon* sp. was fed with [^13^C]-labeled isocyanopupukeanane (**Pu7**) and appearance of labeled 2-formamidopupukeanane (**Pu9**) and 2-isothiocyanatopupukeanane, detected by GC-MS, demonstrated that the isonitrile function is the precursor for the formamido function as well as the isothiocyanato function [152].

Garson *et al.* demonstrated that cyanide is the source for the isonitrile carbons in diisocyanoadociane (**Ica1**). A marine sponge of the genus *Amphimedon* incorporated sodium [^14^C]-cyanide and after usual workup Garson *et al.* isolated [^14^C]-labeled diisocyanoadociane (**Ica1**). Zinc [^14^C]-cyanide and isobutyraldehyde [^14^C]-cyanohydrin are also effective precursors for this experiment due to their better solubility. Furthermore, the sponges were incubated with sodium [2-^14^C]-acetate but it does not appear to be part of the biogenesis of diisocyanoadociane (**Ica1**). Nevertheless they detected labeled carotenoids. Finally they performed tracer studies with [U-^14^C]-alanine, [2-^14^C]-glycine, [U-^14^C]-leucine and [guanidine-^14^C]-arginine but none of these amino acids are precursors for the isonitrile synthesis [131,153].

Experiments with doubly labeled [^13^C,^15^N]-cyanide were performed by Karuso and Scheuer with the sponge *Ciocalypta* sp. and showed that also the nitrogen in the isonitrile has its origin in cyanide. Moreover [^14^C]-cyanide was also incorporated in the sponge *Acanthella* sp. which produces the diterpene kalihinol F (**Kol21**). Some evidence exists that several natural products isolated from sponges may actually have been synthesized by microrganisms associated with these sponges [154]. Thereby the biosynthesis of marine isonitriles differs distinctly from that in terrestrial microorganisms [155].

### 3.1. Sesquiterpenoids

**Figure 38:**
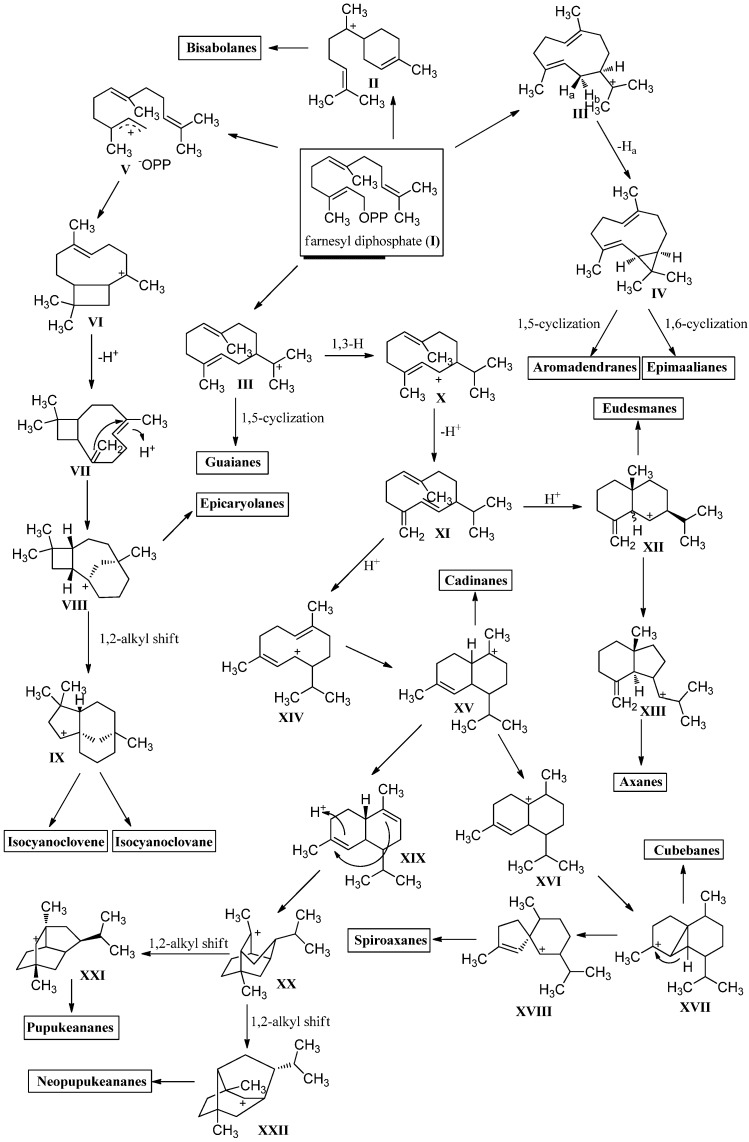
Proposed biosynthesis to sesquiterpenoids.

All sesquiterpenoids are produced from farnesyl pyrophosphate (**I**) and by different cyclizations and Wagner-Meerwein rearrangements the various skeletons will be formed (Figure 38).

First, a 1,6-cyclization of farnesyl pyrophosphate (**I**) leads to the carbenium ion **II** which is the precursor for the bisabolanes (**Bi1**–**Bi15**) [156]. Furthermore, farnesyl diphosphate (**I**) can cyclize to the cyclodecane cation **III**. After formation of the cyclopropane ring with proton abstraction the bicycle **IV** can either convert by 1,5-cyclization to the aromadendranes (**Ar2**–**Ar16**) or by 1,6-cyclization to the epimaalianes (**Ep1**–**Ep8**) [157].

Additionally, farnesyl diphosphate (**I**) can convert to the bicycle **VI** with a cyclobutane ring and after 1,6-cyclization the tricyclic carbocation **VIII** is available which is the precursor for the epicaryolanes (**So8**) [94]. Furthermore, the carbenium ion **VIII** can transform by a 1,2-alkyl shift to the cation **IX**, which is the precursor for isocyanoclovane (**So13**) and isocyanoclovene (**So12**) [94].

Farnesyl diphosphate (**I**) can also cyclize to the cyclodecadiene cation **III**. After 1,5-cyclization of **III**, the guaianes (**Gu1**–**Gu9**) are available [157]. Through a 1,3-H shift and a following deprotonation **XI** is produced and after 1,6-cyclization, the precursor **XII** for the eudesmanes (**Eu2**–**Eu33**) is available. Additionally, the cation **XII** can convert by a Wagner-Meerwein rearrangement into the cation **XIII** which is the precursor for the axanes (**Ax2**–**Ax10**) [158].

In addition to the 1,6-cyclization to **XII**, **XI** can also transform to cation **XV** via 1,6-cyclization. Firstly, this carbocation **XV** is the precursor for the cadinanes (**Ca2**–**Ca34**) [44]. Secondly, after 1,2-H shift to the cation **XVI** and formation of a cyclopropane ring, the precursor **XVII** for the cubebanes (**Fu9**–**Fu11**) is formed [44]. Moreover, after a Wagner-Meerwein rearrangement of **XVII** to **XVIII,** the precursor for the spiroaxanes is obtained [44]. Finally, the cation **XV** can cyclize to the tricyclic system **XX**, which has two options for a 1,2-alkyl shift. The first option leads to cation **XXI** which is the precursor for the pupukeananes (**Pu2**–**Pu10**). The second option is a 1,2-alkyl shift to cation **XXII** which is the precursor for the neopupukeananes (**Pu11**–**Pu13**) [79].

### 3.2. Diterpenoids

Rodriguez *et al.* proposed a possible biosynthetic pathway for diterpenoids in 1994 and postulated the ring closure by *trans*- and *cis*-cyclases [99]. Garson *et al.* extended the biosynthetic pathway to further structures in 2004 [14].

**Figure 39:**
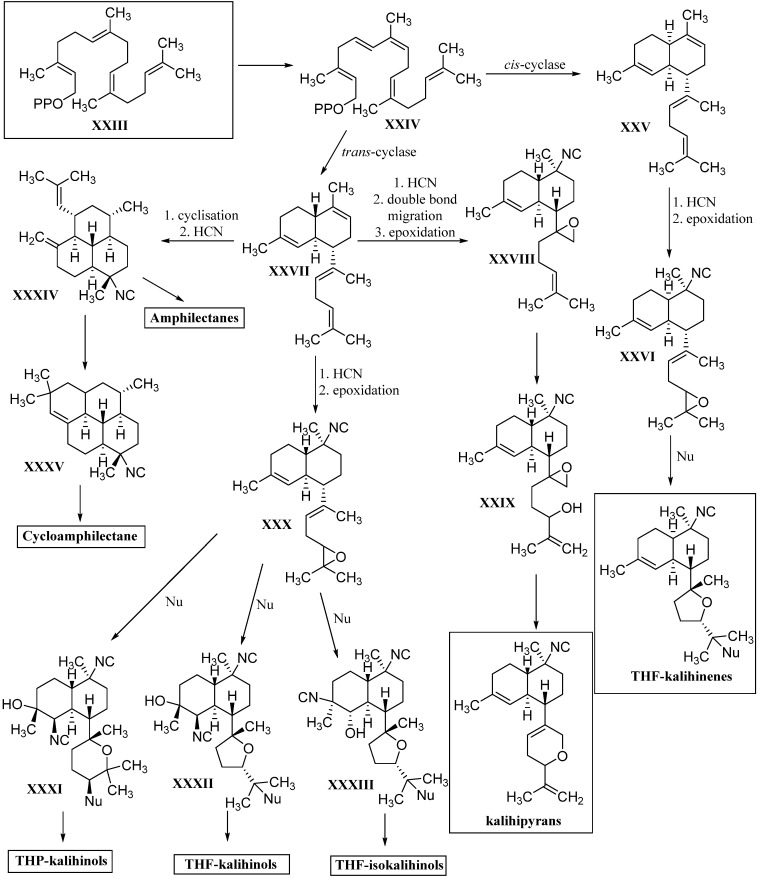
Proposed biosynthesis of diterpenoids [12,14,99].

All Diterpenes are formed from the pyrophosphate **XXIII** (Figure 39). After integration of an additional double bond, the substrate **XXIV** can cyclize with a *cis*-cyclase to the (4*S*,8*S*)-hexahydronaphthalene system **XXV** or with a *trans*-cyclase to the (4*R*,8*S*)-hexahydronaphthalene system **XXVII**.

After addition of hydrogen cyanide and epoxidation of **XXV** to **XXVI**, a nucleophile can attack the epoxide under ring opening and the resulting alcohol can attack at the double bond under formation of the tetrahydrofuran ring in the THF-kalihinenes (**Ken1–Ken12**) [14,99].

For the other marine diterpenes (4*R*,8*S*)-hexahydronaphthalene system **XXVII** is the origin. After addition of hydrogen cyanide, a double bond migration and an epoxidation form the epoxide **XXVIII**. In further steps, the hydroxylated epoxide **XXIX** is formed and after nucleophilic attack of the hydroxy group at the epoxide and subsequent elimination of water, the biogenesis of the kalihipyrans (**Kpy1**–**Kpy4**) is achieved [14,100].

After addition of hydrogen cyanide and epoxidation of **XXVII** without double bond migration, epoxide **XXX** is generated. Garson and Simpson postulated the attack of a nucleophile at both positions of the epoxide **XXX** and after attack of the oxyanion at the double bond under ring closure either the THP-kalihinols (**Kol1**–**Kol19**) or the THF-kalihinols (**Kol20**–**Kol34**) or isokalihinols (**Kol35**–**Kol41**), respectively, should be formed [14,99]. The hydroxy and isonitrile function at C-4 or C-5, respectively, are established by epoxidation of the double bond and following nucleophilic attack at C-4 or C-5.

A further option for the (4*R*,8*S*)-hexahydronaphthalene system **XXVII** is an additional cyclization and subsequent addition of hydrogen cyanide to the dodecahydrophenalene system **XXXIV** which represents the precursor for the amphilectanes (**Amp1**–**Amp26**). Moreover, a further cyclization of **XXXIV** to the tetradecahydropyrene system **XXXV** provides the precursor for the cycloamphilectanes (**Cam1**–**Cam10**) [14,99].

### 3.3. Carbonimidic Dichlorides

**Figure 40:**
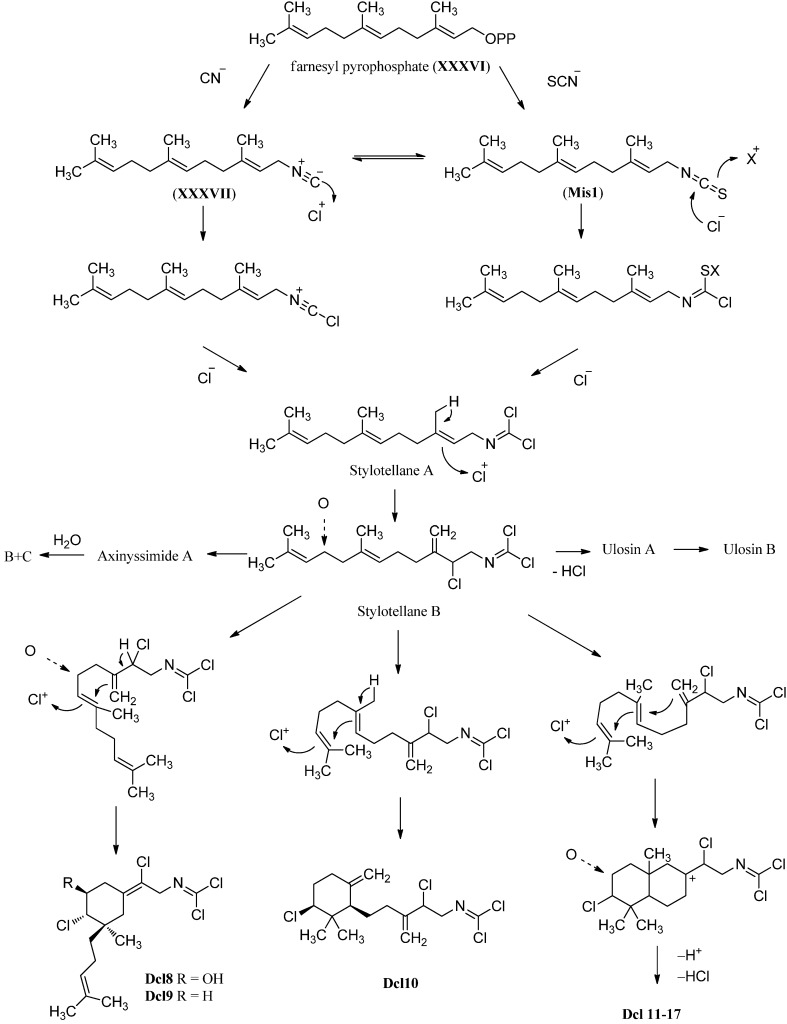
Proposed biosynthesis of carbonimidic dichlorides.

Along with the discovery of the carbonimidic dichlorides in 1977, Wratten already proposed the hypothesis that this uncommon functionality may result from enzymatic chlorination of the corresponding isonitriles or isothiocyanates (Figure 40) [134]. Feeding experiments using radioactive inorganic cyanide in 1997 by Simpson *et al.* proved this hypothesis to be correct [135].

Starting from farnesyl pyrophosphate (**XXXVI**), they were able to show the presence of radioactive stylotellane A in *Stylotella aurantium* fed with potassium [^14^C]-cyanide or isothiocyanate. It could be biosynthesized from farnesyl isocyanide (**XXXVII**) as well as from the corresponding farnesyl isothiocyanate (**Mis1**). Through enzymatic chlorination, stylotellane A can be converted into stylotellane B which is the precursor for both monocyclic carbon skeletons as well as for the bicyclic sesquiterpenoid derivatives, which all result from an enzyme catalyzed chlorinating cyclization. Hydroxylation could either occur from allylic oxidation of stylotellane B at C-9 or after the cyclization. Investigations of the biosynthetic pathway to the cyclic carbonimidic dichlorides were performed by Simpson *et al.* in 2004 using further feeding experiments [139].

## 4. Biological Activity

In general, marine isonitriles have a weak to moderate activity in mammalian cell cytotoxicity assays (10–100 µM) against a broad range of cell lines [15]. Toxicity studies of simple isonitriles revealed surprisingly low toxicity with oral and subcutaneous toxic doses up to LD_50_ = 5 g/kg [159].

Ireland and coworkers reported on the antibacterial activity of kalihinols A (**Kol1**), F (**Kol21**), G (**Kol24**), J (**Kol13**), X (**Kol4**), Y (**Kol9**), 10-formamido-kalihinol F (**Kol22**), 15-formamido-kalihinol F (**Kol23**) and kalihinene (**Ken1**) on the basis of inhibition of the bacterial folate biosynthesis as well as on the growth inhibition of *Bacillus subtilis* [109]. The most potent substances tested were the pyranyl-type kalihinols Y (**Kol9**) and X (**Kol4**), each showing an MIC of 1.56 µg/mL. Nevertheless, negative results of selectivity tests for the folate biosynthesis inhibition mechanism strongly suggest an additional mechanism of action for the pyranyl-bearing derivatives. The furanyl type kalihinols F (**Kol21**) and G (**Kol24**) and kalihinene (**Ken1**) on the other hand are more selective inhibitors than the pyranyl type kalihinols. The substitution pattern at C-10 seems to be important for the potency which becomes apparent in the loss of activity for kalihinol A (**Kol1**) which just differs in the orientation of the isonitrile group at C-10 from the highly active kalihinols Y (**Kol9**) and X (**Kol4**) which bear an *exo*-methylene and an isothiocyanate group, respectively. Finally, the presence of a formamido-substituent, irrespective of its position, is accompanied by a significiant decrease of activity which may be caused by a diminished cellular uptake [109].

Recently, Mayer *et al.* reported that several amphilectanes (**Amp1**, **2**, **10**, **23** and **25**) exhibit promising anti-inflammatory effects, measured in an assay on thromboxane B_2_ and superoxide anion generation from *Escherichia coli* LPS-activated rat brain microglia [160]. Furthermore, several amphilectanes with antimalarial [115,122,127], anti-microbial [102,119,121,126], anti-fungal [112], cytotoxic [50,110], anti-fouling [20], anti-algal [161], anti-tubercular [124,133,161] and anti-photosynthetic [161] activity were reported.

The most promising biological activity of marine isonitriles and their related compounds is their antimalarial activity. A large part of the humanity lives in malaria endangered regions. In 1996, Wright and König gave an overview of the antimalarial effects [115].

Isocycloamphilectenes **Ica** **1**, **2**, **3**, **4** and **9**, cycloamphilectenes **Cam** **5** and **7**, amphilectenes **Amp** **13**, **15**, **16**, **18**, **20** and **22**, isoneoamphilectene **Ina1**, eudesmanes **Eu17** and **Eu21** and axisonitrile-3 (**Sp2**) all demonstrated significiant *in vitro* activity against the malaria parasite *Plasmodium falciparum* [115,162]. Investigations by Wright and König revealed the isocycloamphilectenes diisocyanoadociane (**Ica1**) and (**Ica4**) to display the highest antimalarial activity and selectivity with IC_50_ values of 4 nM and 9 nM, respectively. Structure-activity relationship studies for the amphilectane derivatives using computer-based molecular modeling resulted in the conclusion that the orientation of the C-4 side chain and the negative electrostatic potential in the region of C-7 have a major impact on the activity of these compounds. An α-orientation of the C-4 side chain as for amphilectenes **Amp16**, **18** and **22** results in a decrease of activity due to unfavourable steric influences, which also occur with the OH-group of compound **Amp20**. An isonitrile group at C-7 shows best results for the antimalarial activity. Another functional group at this center or a migration of the Δ^11,20^-*exo*-double bond of **Amp13** to Δ^11,12^ (**Amp15**) causes a distinct loss of antiplasmodial activity, whereas the KB cytotoxicity stays almost unaffected. On the other hand, the nature of the C-20-functionality seems to have no influence on the activity [162]. In agreement with these findings are the results of Chanthathamrongsiri *et al.* who proved the C-8 isonitrile **Amp2** to have a tenfold higher antiplasmodial activity than the isocyanate (**Amp8**) and the isothiocyanate **Amp9**. The C-7-formamide **Amp17** was inactive [123].

The β-lactam bearing amphilectenes monoamphilectines A (**Amp10**), B (**Amp11**) and C (**Amp12**) demonstrated IC_50_ values of 0.60 µg/mL against the chloroquine-resistant W2 strain of *P. falciparum* for **Amp10** and 44.5 nM and 43.3 nM against wild-type *P. falciparum* 3D7 strain for **Amp11** and **Amp12,** respectively [124,125].

In a series of investigations Wright *et al.* showed that the high antiplasmodial activity of **Ica1** (IC_50_ = 4 nM), **Amp13** (IC_50_ = 47 nM) and **Cam5** (IC_50_ = 24 nM) may arise from the inhibition of detoxification processes in *P. falciparum*. **Ica1** and, to a lesser degree, **Amp13** and **Cam5**, inhibit the crystallization of free-heme (ferriprotoporphyrin IX) to hemozoin which may cause a colloidal osmotic instability in *P. falciparum*. Additionally, a concomitant impairment of H_2_O_2_ detoxification processes may arise [41,161,163].

Both effects are attributed to an interaction of the marine isonitriles with hemoglobin in a cavity close to heme [41]. Initial studies proposed a direct binding to the free heme iron [161].

In spite of the variety of marine isocyanoterpenoids showing promising antimalarial activity, only a few kalihinane-type isonitriles have been evaluated for their potency in this respect. In 1998, Miyaoka demonstrated kalihinol A (**Kol1**) to have potent antimalarial activity against the drug-resistant FCR-3 strain of *Plasmodium falciparum* (IC_50_ = 1.2 nM) while 10-*epi*-kalihinol I (**Kol15**), 5,10-bisisothiocyanatokalihinol G (**Kol34**), kalihinene (**Ken1**) and 6-hydroxy-kalihinene (**Ken5**) were somewhat less active [105].

In 2015, Daub *et al.* proved kalihinol B (**Kol**) to exhibit antiplasmodial activity (IC_50_ = 8.4 nM for the wild type of the 3D7 strain of *P. falciparum* and 4.6 nM for chloroquine-resistant Dd2 strain) in a similar range as kalihinol A (**Kol1**). The group developed a twelve-step synthesis towards kalihinol B (**Kol20**), thus establishing a synthetic route to other kalihinol derivatives for further antiplasmodial testing [164].

As is obvious from the promising activities against *P. falciparum*, the marine isonitriles may well be viewed as attractive lead structures for new drugs against malaria.

## 5. Conclusions

**Figure 41:**
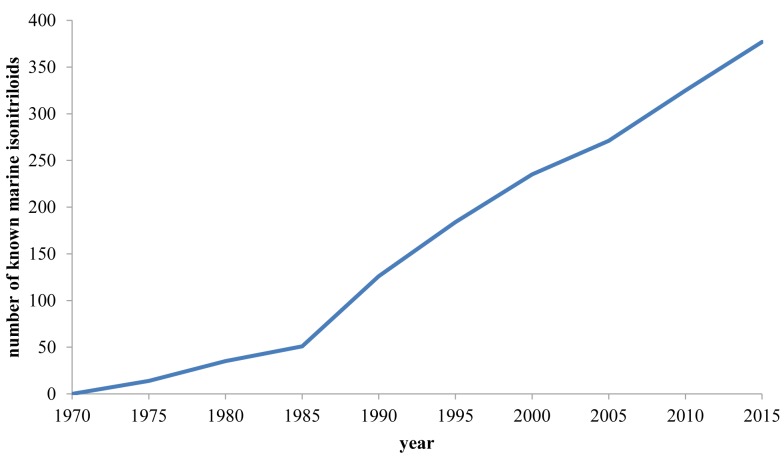
Number of new marine isonitriles and related compounds found until 2015.

Since the last review on marine isonitriles in 2000, the number of isolated marine isonitriles and their related compounds has continued to increase steadily (Figure 41). Today, the marine isonitriles and their derivatives are still very interesting molecules in terms of their structure, their biosynthesis, and their potential application in the biomedical field. Although it can safely be assumed that the malaria parasite *Plasmodium falciparum* and the isonitrile-bearing marine organisms have never met in recent evolution, the pronounced antimalarial activity of natural isonitriles is one of several reasons to continue research on these odd, yet fascinating molecular gemstones from the oceans.

## 6. Overview about All Marine Isonitriles and Their Related Compounds

All known marine isonitriloids are listed in the following table (Table 1).

**Table 1:** Overview of all known marine isonitriles and their related compounds.

Trivial Name (Structure)	Organism	Origin	Year	References
***Sesquiterpenoids***				
***Axanes***				
axisonitrile-1 (**Ax2**)	*Axinella cannabina*	Bay of Taranto, Italy	1973	[2]
axisothiocyanate-1 (**Ax3**)	*Axinella cannabina*	Bay of Taranto, Italy	1973	[2]
axamide-1 (**Ax4**)	*Axinella cannabina*	Bay of Taranto, Italy	1974	[4]
axisonitrile-4 (**Ax5**)	*Axinella cannabina*	Bay of Taranto, Italy	1977	[16]
axisothiocyanate-4 (**Ax6**)	*Axinella cannabina*	Bay of Taranto, Italy	1977	[16]
axamide-4 (**Ax7**)	*Axinella cannabina*	Bay of Taranto, Italy	1977	[16]
cavernoisonitrile (**Ax8**)	*Acanthella* cf. *cavernosa*	Hachijo-jima Island, Japan	1992	[19]
	*Acanthella cavernosa*	Hachijo-jima Island, Japan	1996	[20]
(−)-cavernothiocyanate (**Ax9**)	*Acanthella* cf. *cavernosa*	Hachijo-jima Island, Japan	1992	[19]
	*Acanthella cavernosa*	Hachijo-jima Island, Japan	1996	[20]
10-isothiocyanato-11-axene (**Ax10**)	*Acanthella* cf. *cavernosa*	Hachijo-jima Island, Japan	1992	[19]
	*Phyllidia ocellata*	Hachijo-jima Island, Japan	1992	[19]
	*Acanthella cavernosa*	Hachijo-jima Island, Japan	1996	[20]
***Eudesmanes***				
acanthellin-1 (**Eu2**)	*Acanthella acuta*	Bay of Naples, Italy	1974	[3]
	*Axinella cannabina*	Bay of Taranto, Porto Cesareo, Italy	1984	[21]
R=NCS (**Eu3**)	*Axinella cannabina*	Bay of Taranto, Porto Cesareo, Italy	1984	[21]
R=NHCHO (**Eu4**)	*Axinella cannabina*	Bay of Taranto, Porto Cesareo, Italy	1984	[21]
R=NC (**Eu5**)	*Axinella cannabina*	Bay of Taranto, Porto Cesareo, Italy	1984	[21]
R=NCS (**Eu6**)	*Axinella cannabina*	Bay of Taranto, Porto Cesareo, Italy	1984	[21]
R=NHCHO (**Eu7**)	*Axinella cannabina*	Bay of Taranto, Porto Cesareo, Italy	1984	[21]
6α-isocyano-5α-H,7α-H, 10α-eudesm-4(14)-ene (**Eu8**)	*Axinella cannabina* and *Acanthella acuta*	Bay of Taranto, Porto Cesareo, Italy	1987	[22]
6α-isothiocyano-5α-H,7α-H, 10α-eudesm-4(14)-ene (**Eu9**)	*Axinella cannabina* and *Acanthella acuta*	Bay of Taranto, Porto Cesareo, Italy	1987	[22]
6α-formamido-5α-H,7α-H, 10α-eudesm-4(14)-ene (**Eu10**)	*Axinella cannabina*	Bay of Taranto, Porto Cesareo, Italy	1987	[22]
halichonadin C (**Eu11**)	*Halichondria* sp.	Unten Port, Okinawa, Japan	2005	[24]
	*Phyllidia ocellata*	Mudjimba Island, Mooloolaba, Australia	2015	[94]
acanthene B (**Eu12**)	*Acanthella* sp.	Conehead Point, Rennell Sound, Graham Island, British Columbia	1993	[23]
	*Acanthella cavernosa*	Tani’s Reef, Gneerings Reef, Mooloolaba, Australia	2007	[32,58]
acanthene C (**Eu13**)	*Cadlina luteomarginata*	Conehead Point, Rennell Sound, Graham Island, British Columbia	1993	[23]
halichonadin B (**Eu14**)	*Halichondria* sp.	Unten Port, Okinawa, Japan	2005	[24]
halichonadin A (**Eu15**)	*Halichondria* sp.	Unten Port, Okinawa, Japan	2005	[24]
11-isocyano-7β-H-eudesm-5-ene (**Eu16**)	*Axinella cannabina*	Taranto, near Porto Cesareo, Italy	1987	[25]
	*Phyllidia pustulosa*	Negros Island, Cebu Island, San Sebastian, Cebu, Philippines	1991	[86]
	*Acanthella* sp. and *Cadlina luteomarginata*	Conehead Point, Rennell Sound, Graham Island, British Columbia	1993	[23]
	*Axinyssa ambrosia*	Santa Marta Bay, Caribbean Coast, Colombia	2002	[26]
	*Acanthella cavernosa*	Tani’s Reef, Gneerings Reef, Mooloolaba, Australia	2007	[32,58]
	*Phyllidiella pustulosa*	Vietnam	2010	[27]
11-isothiocyano-7β-H-eudesm-5-ene (**Eu17**)	*Axinella cannabina*	Taranto, near Porto Cesareo, Italy	1987	[25]
	*Acanthella pulcherrima*	Weed Reef, Darwin, Australia	1988	[28]
	*Phyllidia pustulosa*	Negros Island, Cebu Island, San Sebastian, Cebu, Philippines	1991	[86]
	*Acanthella klethra*	Pelorus Islands, Queensland, Australia	1992	[30]
	*Acanthella* sp. and *Cadlina luteomarginata*	Conehead Point, Rennell Sound, Graham Island, British Columbia	1993	[23]
	*Axinyssa ambrosia*	Santa Marta Bay, Caribbean Coast, Colombia	2002	[26]
	*Acanthella cavernosa*	Coral Gardens, Gneerings Reef, Mooloolaba, Australia	2007	[32,58]
11-formamido-7β-H-eudesm-5-ene (**Eu18**)	*Axinella cannabina*	Taranto, near Porto Cesareo, Italy	1987	[25]
	*Axinyssa ambrosia*	Santa Marta Bay, Caribbean Coast, Colombia	2002	[26]
4-Isothiocyanatoeudesm-11-ene (**Eu19**)	*Axinyssa ambrosia*	Santa Marta Bay, Caribbean Coast, Colombia	2002	[26]
4-isothiocyanatoeudesm-11-ene (**Eu20**)	*Acanthella klethra*	Pelorus Islands, Queensland, Australia	1992	[30]
	*Acanthella cavernosa*	Heron Island, Great Barrier Reef, Australia	2000	[31]
	*Acanthella cavernosa*	Mudjimba Island, Mooloolaba, Australia	2007	[32,58]
	*Axinyssa isabela*	Isabel Island, Nayarit, Mexico	2008	[33]
4-formamidoeudesm-11-ene (**Eu21**)	*Axinyssa ambrosia*	Santa Marta Bay, Caribbean Coast, Colombia	2002	[26]
stylostelline (**Eu22**)	*Stylotella* sp.	South East of New Caledonia	1987	[34]
*ent*-stylotelline (**Eu23**)	*Phyllidiella pustulosa*	Hainan Island, South China Sea	2004	[35]
4-isothiocyanatoeudesm-11-ene (**Eu24**)	*Acanthella klethra*	Pelorus Islands, Queensland, Australia	1992	[30]
	*Acanthella* sp. and *Cadlina luteomarginata*	Conehead Point, Rennell Sound, Graham Island, British Columbia	1993	[23]
axinisothiocyanate M (**Eu25**)	*Axinyssa isabela*	Isabel Island, Nayarit, Mexico	2008	[33]
axinisothiocyanate N (**Eu26**)	*Axinyssa isabela*	Isabel Island, Nayarit, Mexico	2008	[33]
4-formamidoeudesm-7-ene (**Eu27**)	*Axinyssa* sp.	South China Sea	2008	[36]
4-formamidoeudesman-11-ol (**Eu28**)	*Axinyssa* sp.	South China Sea	2008	[36]
halichonadin E (**Eu29**)	*Halichondria* sp.	Unten Port, Okinawa, Japan	2008	[37]
halichonadin G (**Eu30**)	*Halichondria* sp.	Unten Port, Okinawa, Japan	2011	[38]
halichonadin H (**Eu31**)	*Halichondria* sp.	Unten Port, Okinawa, Japan	2011	[38]
halichonadin I (**Eu32**)	*Halichondria* sp.	Unten Port, Okinawa, Japan	2011	[38]
halichonadin J (**Eu33**)	*Halichondria* sp.	Unten Port, Okinawa, Japan	2011	[38]
***Cadinanes***				
(–)-10-isocyano-4-amorphene (**Ca2**)	*Halichondria* sp.	North coast of O’ahu, Hawaii	1975	[6,39,40]
	*Axinyssa*	Gun Beach, Guam	1989	[43]
	*Phyllidia ocelata*	Kamikoshiki-jima Island, Japan	1996	[42]
(–)-10-isothiocyanato-4-amorphene (**Ca3**)	*Halichondria* sp.	North coast of O’ahu, Hawaii	1975	[6,39,40]
	*Phyllidia pustulosa*	Yakushima Island, Japan	1996	[42]
	*Phyllidiella pustulosa*	Vietnam	2010	[27]
(–)-10-isoformamido-4-amorphene (**Ca4**)	*Halichondria* sp.	North coast of O’ahu, Hawaii	1975	[6,39,40]
R=NC (**Ca5**)	*Axinella cannabina*	Bay of Taranto, Porto Cesareo, Italy	1986	[44]
R=NCS (**Ca6**)	*Axinella cannabina*	Bay of Taranto, Porto Cesareo, Italy	1986	[44]
R=NHCHO (**Ca7**)	*Axinella cannabina*	Bay of Taranto, Porto Cesareo, Italy	1986	[44]
(3S*,5R*,6R*,9R*)-3-isocyano-1(10)-cadinene (**Ca8**)	*Axinyssa aplysinoides*	West of Malakal Harbor, Palau	1995	[46]
halipanicine (**Ca9**)	*Halochondria panacea*	Okinawa, Japan	1991	[45]
(3S*,5R*,6R*,9R*)-3-formamido-1(10)-cadinene (**Ca10**)	*Axinyssa aplysinoides*	West of Malakal Harbor , Palau	1995	[46]
	*Halichondria* sp.	PP Island, Andaman Sea, Southern Thailand	2011	[59]
4α-isocyano-9-amorphene (**Ca11**)	*Phyllidia pustulosa*	Hachijo-jima Island, Japan	1991	[47]
10α-isocyano-4-amorphene (**Ca12**)	*Acanthella* cf. *cavernosa*	Hachijo-jima Island, Japan	1992	[19]
	*Phyllidia ocellata*	Hachijo-jima Island, Japan	1992	[19]
10-isocyano-4-cadinene (**Ca13**)	*Phyllidia pustulosa + varicosa*	Kamikoshiki-jima/Shimokoshiki Island, Japan	1996	[42]
10-isothiocyanate-4-cadinene (**Ca14**)	*Acanthella cavernosa*	Heron Island, Great Barrier Reef, Australia	2000	[31]
	*Stylissa* sp.	Iriomote Island, Okinawa, Japan	2004	[50]
	*Acanthella cavernosa*	Tani’s Reef, Gneerings Reef, Mooloolaba, Australia	2007	[32,58]
R=NCS (**Ca15**)	*Acanthella pulcherrima*	Weed Reef, Darwin, Australia	1988	[28]
	*Acanthella cavernosa*	Coral Gardens, Gneerings Reef, Mooloolaba, Australia	2007	[32,58]
(1*R*,6*S*,7*S*,10*S*)-10-isothiocyanato-4-amorphene (**Ca16**)	*Axinella fenestratus*	Fiji	1991	[49]
	*Topsentia* sp., *Acanthella cavernosa*	Thailand	1991	[49]
	*Acanthella cavernosa*	Tani’s Reef, Gneerings Reef, Mooloolaba, Australia	2007	[32,58]
(1R*,6R*,7S*,10S*)-10-isothiocyanatocadin-4-ene (**Ca17**)	*Stylissa* sp.	Coral reef, Iriomote Island, Okinawa, Japan	2004	[50]
axinisothiocyanate K (**Ca18**)	*Axinyssa*	Gulf of California	2008	[51]
(1R*,4S*,6R*,7S*)-4-isothiocyanato-9-amorphene (**Ca19**)	*Axinella fenestratus*	Fiji	1991	[49]
	*Topsentia* sp., *Acanthella cavernosa*	Thailand	1991	[49]
(1S*,4S*,6S*,7R*)-4-thiocyanato-9-cadinene (**Ca20**)	*Trachyopsis aplysinoides*	Palau	1989	[52]
	*Phyllidia pustulosa*	Katsuura, Kii Penisula, Japan	1998	[53]
(1S*,4S*,7R*,10S*)-10-isocyano-5-cadinen-4-ol (**Ca21**)	*Phyllidia pustulosa*	Katsuura, Kii Penisula, Japan	1998	[53]
10-isothiocyanatoamorph-5-en-4-ol (**Ca22**)	*Axinella fenestratus*	Fiji	1991	[49]
	*Topsentia* sp., *Acanthella cavernosa*	Thailand	1991	[49]
axinisothiocyanate J (**Ca23**)	*Axinyssa*	Gulf of California	2008	[51]
axinisothiocyanate A (**Ca24**)	*Axinyssa*	Gulf of California	2008	[51]
axinisothiocyanate B (**Ca25**)	*Axinyssa*	Gulf of California	2008	[51]
axinisothiocyanate C (**Ca26**)	*Axinyssa*	Gulf of California	2008	[51]
axinisothiocyanate D (**Ca27**)	*Axinyssa*	Gulf of California	2008	[51]
axinisothiocyanate E (**Ca28**)	*Axinyssa*	Gulf of California	2008	[51]
axinisothiocyanate F (**Ca29**)	*Axinyssa*	Gulf of California	2008	[51]
axinisothiocyanate G (**Ca30**)	*Axinyssa*	Gulf of California	2008	[51]
axinisothiocyanate H (**Ca31**)	*Axinyssa*	Gulf of California	2008	[51]
axinisothiocyanate I (**Ca32**)	*Axinyssa*	Gulf of California	2008	[51]
axinisothiocyanate L (**Ca33**)	*Axinyssa*	Gulf of California	2008	[51]
Axiplyn C (**Ca34**)	*Axinyssa aplysinoides*	Misali Island, Tanzania	2008	[54]
***Spiroaxanes***				
(+)-axisonitril-3 (**Sp2**)	*Axinella cannabina*	Bay of Taranto, Italy	1976	[55]
	*Acanthella acuta*	Mediterranean sea	1987	[57]
	*Topsentia* sp.	Thailand	1991	[49]
	*Acanthella klethra*	Pelorus Islands, Queensland, Australia	1992	[30]
	*Axinyssa aplysinoides*	Mutok Harbor, Pohnpei	1992	[69]
	*Acanthella* cf. *cavernosa*	Hachijo-jima Island, Japan	1992	[19]
	*Phyllidia ocellata*	Hachijo-jima Island, Japan	1992	[19]
	*Phyllidia pustulosa*	Yakushima/Kuchinoerabu-jima/Tenegashima Islands, Japan	1996	[42]
	*Acanthella cavernosa*	Tani’s Reef, Gneerings Reef, Mooloolaba, Australia	2007	[32,58]
	*Acanthella* sp.	Yalong Bay, Hainan Province, China	2009	[103]
	*Phyllidia ocellata*	Mudjimba Island, Mooloolaba, Australia	2015	[94]
(+)-axisothiocyanate-3 (**Sp3**)	*Axinella cannabina*	Bay of Taranto, Italy	1976	[55]
	*Acanthella klethra*	Pelorus Islands, Queensland, Australia	1992	[30]
	*Acanthella* cf. *cavernosa*	Hachijo-jima Island, Japan	1992	[19]
	*Phyllidia ocellata*	Hachijo-jima Island, Japan	1992	[19]
	*Acanthella cavernosa*	Hachijo-jima Island, Japan	1996	[20]
	*Acanthella cavernosa*	Tani’s Reef, Gneerings Reef, Mooloolaba, Australia	2007	[32,58]
(−)-axamide-3 (**Sp4**)	*Axinella cannabina*	Bay of Taranto, Italy	1976	[55]
	*Acanthella* cf. *cavernosa*	Hachijo-jima Island, Japan	1992	[19]
	*Phyllidia ocellata*	Hachijo-jima Island, Japan	1992	[19]
axisocyanate-3 (**Sp5**)	*Acanthella cavernosa*	Mudjimba Island, Mooloolaba, Australia	2007	[32,58]
(−)-axisonitrile-3 (**Sp6**)	*Halichondria* sp.	PP Island, Andaman Sea, Southern Thailand	2011	[59]
(+)-axamide (**Sp7**)	*Axinella cannabina*	Bay of Taranto, Italy	1976	[55]
	*Acanthella cavernosa*	Hachijo-jima Island, Japan	1996	[20]
	*Halichondria* sp.	PP Island, Andaman Sea, Southern Thailand	2011	[59]
10-epi-axisonitrile-3 (**Sp8**)	*Phyllidia pustulosa*	Yakushima/Kuchinoerabu-jima Islands, Japan	1996	[42]
	*Geodia exigua*	Oshima, Kagoshima Prefecture, Japan	2003	[60]
exiguamide (**Sp9**)	*Geodia exigua*	Oshima, Kagoshima Prefecture, Japan	2003	[60]
exicarbamate (**Sp10**)	*Geodia exigua*	Oshima, Kagoshima Prefecture, Japan	2003	[60]
exigurin (**Sp11**)	*Geodia exigua*	Oshima, Kagoshima Prefecture, Japan	2003	[60]
3-Oxoaxisonitrile-3 (**Sp12**)	*Acanthella* sp.	South China Sea	2006	[61]
R=NC (**Sp13**)	*Acanthella acuta*	Bay of Naples, Italy	1987	[62]
R=NCS (**Sp14**)	*Acanthella acuta*	Bay of Naples, Italy	1987	[62]
(2*R*,5*R*,10*S*)-2-isothiocyanato-6-axene (**Sp15**)	*Trachyopsis aplysinoides*	Palau	1989	[52]
	*Axinyssa aplysinoides*	Palau	1992	[69]
	*Amorphinopsis foetida*	Madang region, Papua New Guinea	2006	[63]
(2*R*,5*R*,10*S*)-2-formamido-6-axene (**Sp16**)	*Trachyopsis aplysinoides*	Palau	1989	[52]
	*Axinyssa aplysinoides*	Palau	1992	[69]
	*Axinyssa aplysinoides*	Mele Bay, Vanuatu	2006	[63]
R=NHCHO (**Sp17**)	*Amorphinopsis foetida*	Madang region, Papua New Guinea	2006	[63]
*N*-Phenethyl-2-formamido-6-axene (**Sp18**)	*Amorphinopsis foetida* and *Axinyssa aplysinoides*	Madang region, Papua New guinea and Mele Bay, Vanuatu	2006	[63]
*N*-Phenethyl-2-formamido-6-axene (**Sp19**)	*Amorphinopsis foetida* and *Axinyssa aplysinoides*	Madang region, Papua New guinea and Mele Bay, Vanuatu	2006	[63]
***Aromadendranes***				
axisonitrile-2 (**Ar2**)	*Axinella cannabina*	Bay of Taranto, Italy	1974	[4]
	*Acanthella cannabina*	Taranto, near Porto Cesareo, Italy	1986	[44]
	*Phyllidia pustulosa*	Hachijo-jima Island, Japan	1991	[47]
	*Acanthella* cf. *cavernosa*	Hachijo-jima Island, Japan	1992	[19]
	*Acanthella cavernosa*	Hachiji-jima Island, Japan	1996	[20]
	*Phyllidia ocellata*	Mudjimba Island, Mooloolaba, Australia	2015	[94]
axamide-2 (**Ar3**)	*Axinella cannabina*	Bay of Taranto, Italy	1974	[4]
	*Hexabrandies sanguinens*	South China Sea	2007	[68]
	*Halichondria* sp.	PP Island, Andaman Sea, Southern Thailand	2011	[59]
axisothiocyanate-2/epipolasin B (**Ar4**)	*Axinella cannabina*	Bay of Taranto, Italy	1974	[4]
	*Epipolasis kushimotoensis*		1985	[64]
	*Axinyssa aplysinoides*	Ant Atoll, Pohnpei	1992	[69]
	*Acanthella cavernosa*	Hachijo-jima Island, Japan	1996	[20]
	*Axinyssa* sp.	Tsutsumi Island, Fukuoka prefecture, Japan	2002	[165]
	*Acanthella cavernosa*	Tani’s Reef, Gneerings Reef, Mooloolaba, Australia	2007	[32,58]
epipolasinthiourea-B (**Ar5**)	*Epipolasis kushimotoensis*		1985	[64]
10α-isocyanoalloaromadendrane (**Ar6**)	*Acanthella cannabina*	Taranto, near Porto Cesareo, Italy	1987	[25]
10α-formamidoalloaromadendrane (**Ar7**)	*Acanthella cannabina*	Taranto, near Porto Cesareo, Italy	1987	[25]
10α-isothiocyanatoalloaromadendrane (**Ar8**)	*Acanthella cannabina*	Taranto, near Porto Cesareo, Italy	1987	[25]
	*Acanthella cavernosa*	Tani’s Reef, Gneerings Reef, Mooloolaba, Australia	2007	[32,58]
(1*R*,4*S*,5*S*,6*R*,7*S*,10*R*)-(+)-isothiocyanatoalloaromadendrane (**Ar9**)	*Acanthella cavernosa*	Hachijo-jima Island, Japan	1996	[20]
	*Acanthella* sp.	Yalong Bay, Hainan Province, China	2009	[103]
	*Phyllidiella pustulosa*	Vietnam	2010	[27]
Halochonadin F (**Ar11**)	*Halichondria* sp.	Unten Port, Okinawa, Japan	2008	[66]
R=NCS (**Ar12**)	*Axinyssa aplysinoides*	Ant Atoll, Pohnpei	1992	[69]
R=NC (**Ar13**)	*Acanthella acuta*	Bay of Naples, Italy	1987	[62]
	*Acanthella acuta*	Banyuls, France	1988	[67]
	*Acanthella cavernosa*	Tani’s Reef, Gneerings Reef, Mooloolaba, Australia	2007	[32,58]
	*Phyllidiella pustulosa*	Vietnam	2010	[27]
	*Phyllidia ocellata*	Mudjimba Island, Mooloolaba, Australia	2015	[94]
R=NCS (**Ar14**)	*Acanthella acuta*	Bay of Naples, Italy	1987	[62]
	*Acanthella acuta*	Banyuls, France	1988	[67]
	*Acanthella cavernosa*	Tani’s Reef, Gneerings Reef, Mooloolaba, Australia	2007	[32,58]
	*Phyllidiella pustulosa*	Vietnam	2010	[27]
R=NCO (**Ar15**)	*Acanthella cavernosa*	Coral gardens, Gneerings reef, Mooloolba, Australia	2007	[32,58]
	*Acanthella acuta*	Banyuls, France	1988	[67]
R=NHCHO (**Ar16**)	*Hexabrandies sanguinens*	South China Sea	2007	[68]
***Epimaalianes***				
R=NC (**Ep2**)	*Cadlina luteomarginata*	San Diego, California	1982	[70]
	*Acanthella* sp. and *Cadlina luteomarginata*	Conehead Point, Rennell Sound, Graham Island, British Columbia	1993	[23]
(−)-epipolasin A (**Ep3)**	*Cadlina luteomarginata*	San Diego, California	1982	[70]
	*Acanthella pulcherrima*	Weed Reef, Darwin, Australia	1988	[28]
	*Acanthella* sp.	Conehead Point, Rennell Sound, Graham Island, British Columbia	1993	[23]
	*Axinyssa* sp. nov.	Great Barrier Reef	1997	[71]
	*Axinyssa* sp.	Tsutsumi Island, Fukuoka prefecture, Japan	2003	[165]
R=NHCHO (**Ep4**)	*Acanthella* sp. and *Cadlina luteomarginata*	Conehead Point, Rennell Sound, Graham Island, British Columbia	1993	[23]
(+)-epipolasin A (**Ep5**)	*Epipolasis kushimotoensis*		1985	[64]
	*Axinyssa aplysinoides*	Ant Atoll, Pohnpei	1992	[69]
R=NHCSNHCH_2_CH_2_Ph (**Ep6**)	*Epipolasis kushimotoensis*		1985	[64]
R=NC (**Ep7**)	*Axinella cannabina*	Bay of Taranto, Italy	1985	[72]
R=NCS (**Ep8**)	*Axinella cannabina*	Bay of Taranto, Italy	1985	[72]
R=NHCHO (**Ep9**)	*Axinella cannabina*	Bay of Taranto, Italy	1985	[72]
***Pupukeananes***				
9-isocyanopupukeanane (**Pu2**)	*Phyllidia varicosa* and *Cyocalypta* sp.	Pupukea, north shore of O’ahu, Hawaii	1975	[73]
	*Phyllidia varicosa* Lamarck 1801	Pupukea, north shore of O’ahu, Hawaii	1979	[77]
	*Phyllidia bourguini*	Hachijo-jima Island, Japan	1990	[75]
	*Phyllidia pustulosa*	Hachijo-jima Island, Japan	1991	[47]
9-epi-isocyanopupukeanane (**Pu3**)	*Phyllidia bourguini*	Hachijo-jima Island, Japan	1990	[75]
	*Phyllidia pustulosa*	Hachijo-jima Island, Japan	1991	[47]
9-isothiocyanatopupukeanane (**Pu4**)	*Axinyssa* sp. nov.	Great Barrier Reef	1997	[71]
9-thiocyanatopupukeanane (**Pu5**)	*Phyllidia varicosa Axinyssa aculeata*	Pramuka Island, Indonesia	2003	[76]
	*Phyllidiella pustulosa*	Vietnam	2010	[27]
9-epi-thiocyanatopupukeanane (**Pu6**)	*Phyllidia varicosa Axinyssa aculeata*	Pramuka Island, Indonesia	2003	[76]
	*Phyllidiella pustulosa*	Vietnam	2010	[27]
2-isocyanopupukeanane (**Pu7**)	*Phyllidia varicosa* Lamarck 1801	Pupukea, north shore of O’ahu, Hawaii	1979	[77]
2-thiocyanatopupukeanane (**Pu8**)	*Axinyssa aplysinoides*	Palau	1992	[69]
2-formamidopupukeanane (**Pu9**)	*Phyllidia coelestis* Bergh	Koh-Ha Islets, Thailand	2013	[78]
5-isothiocyanatopupukeanane (**Pu10**)	*Axinyssa*	Gun Beach, Guam	1989	[43]
9-isocyanoneopupukeanane (**Pu11**)	*Ciocalypta* sp.	O’ahu, Hawaii	1989	[155]
2-thiocyanatoneopupukeanane (**Pu12**)	*Phycopsis terpnis*	Okinawa, Japan and Pohnpei	1991	[80]
	*Axinyssa aplysinoides*	Mutok Harbor, Pohnpei	1992	[69]
	*Phyllidia pustulosa*	Kuchinoerabu/Tanegoshima Island, Japan	1996	[42]
4-thiocyanatoneopupukeanane (**Pu13**)	*Phycopsis terpnis*	Okinawa, Japan and Pohnpei	1991	[80]
	*Phyllidia pustulosa*	Tanegoshima Island, Japan	1996	[42]
2-isocyanoallopupukeanane (**Pu14**)	*Phyllidia pustulosa*	Hachijo-jima Island, Japan	1991	[47]
1-formamido-10(1 → 2)-abeopupukeanane (**Pu15**)	*Phyllidia coelestis* Bergh	Koh-Ha Islets, Thailand	2013	[78]
***Bisabolanes***				
3-isocyanotheonellin (**Bi2**)	*Phyllidia* sp.	Colombo, Sri Lanka	1986	[74]
	*Phyllidia pustulosa*	Hachijo-jima Island, Japan	1991	[47]
	*Phyllidiella pustulosa*	Hainan Island, South China Sea	2004	[35]
	*Lipastrotethya ana*	Lingshui Bay, Hainan	2007	[166]
	*Raphoxya* sp.	Blue Hole, Guam	2012	[82]
3-isothiocyanatotheonellin (**Bi3**)	*Theonella* cf. *swinhoei*	Okinawa	1984	[81]
	*Phyllidia pustulosa*	Tanegoshima Island, Japan	1996	[42]
	*Axinyssa*	Micronesia	1999	[90]
	*Lipastrotethya ana*	Lingshui Bay, Hainan	2007	[91]
	*Raphoxya* sp.	Blue Hole, Guam	2012	[82]
3-formamidotheonellin (**Bi4**)	*Theonella* cf. *swinhoei*	Okinawa	1984	[81]
	*Axinyssa* sp.	Inner coral reef, Andaman Sea, Thailand	2014	[92]
3-isocyanatotheonellin (**Bi5**)	*Raphoxya* sp.	Blue Hole, Guam	2012	[82]
7-isocyano-7,8-dihydro-α-bisabolene (**Bi6**)	*Ciocalypta* sp.	Pupukea, O’ahu, Hawaii	1986	[74]
	*Phyllidia pustulosa*	Hachijo-jima Island, Japan	1991	[47]
	*Acanthella cavernosa*	Tani’s Reef, Gneerings Reef, Mooloolaba, Australia	2007	[32,58]
7-isothiocyanato-7,8-dihydro-α-bisabolene (**Bi7**)	*Halichondria* sp.	Ponape, Marshall Islands	1986	[83]
	*Phyllidia pustulosa*	Negros Island, Cebu Island, San Sebastian, Cebu, Philippines	1991	[86]
	*Acanthella* cf. *cavernosa*	Hachijo-jima Island, Japan	1992	[19]
	*Phyllidia pustulosa*	Hachijo-jima Island, Japan	1992	[19]
	*Acanthella cavernosa*	Tani’s Reef, Gneerings Reef, Mooloolaba, Australia	2007	[32,58]
*N*,*N*′*-*Bis(6*R*,7*S*)-7,8-dihydro-α-bisabolane (**Bi8**)	*Halichondria* sp.	Ponape, Marshall Islands	1986	[83]
7-formamido-7,8-dihydro-α-bisabolene (**Bi9**)	*Axinyssa* sp.	Sanya, Hainan Province, China	2008	[84]
7-isocyano-7,8-dihydro-α-bisabolene (**Bi10**)	*Ciocalypta* sp.	Pupukea, O’ahu, Hawaii	1986	[74]
7-isocyanato-7,8-dihydro-α-bisabolene (**Bi11**)	*Ciocalypta* sp.	Pupukea, O’ahu, Hawaii	1986	[74]
R=NC (**Bi12**)	*Phyllidiella pustulosa*	Hainan Island, South China Sea	2004	[35]
(*E*)-4-isocyanobisabolane-7,10-diene (**Bi13**)	*Axinyssa*	Okinawa	2002	[85]
3-isocyanobisabolane-8,10-diene (**Bi14**)	*Phyllidia pustulosa*	Negros Island, Cebu Island, San Sebastian, Cebu, Philippines	1991	[86]
3-formamidobisabolene-8,10-diene (**Bi15**)	*Halichondria* cf. *lengenfeldi*	Palau	1991	[86]
axinythiocyanate A (**Bi16**)	*Axinyssa isabela*	Isabel Island, Nayarit, Mexico	2008	[33]
3-isocyano-7,8-epoxy-α-bisabolane (**Bi17**)	*Axinyssa* sp.	Hainan	2010	[87]
3-formamido-7,8-epoxy-α-bisabolane (**Bi18**)	*Axinyssa* sp.	Hainan	2010	[87]
	*Axinyssa* sp.	Inner coral reef, Andaman Sea, Thailand	2014	[92]
axinysaline B (**Bi19**)	*Axinyssa* sp.	Formosa	2014	[88]
axinysaline A (**Bi20**)	*Axinyssa* sp.	Formosa	2014	[88]
7α,8α-epoxy theonellin isothiocyanate (**Bi21**)	*Phycopsis* sp.	Mandapam Coast, Gulf of Mannar, Tamilnadu, India	2009	[89]
3-formamidobisabolane-14(7),9-dien-8-ol (**Bi22**)	*Axinyssa*	Micronesia	1999	[90]
3-formamidobisabolane-14(7),9-dien-8-one (**Bi23**)	*Axinyssa*	Micronesia	1999	[90]
11-ethoxy-3-formamidotheonellin (**Bi24**)	*Axinyssa* aff. *variabilis*	Lingshui Bay, Hainan	2007	[91]
7-ethoxy-3-formamidobisabolane-8,10-diene (**Bi25**)	*Axinyssa* aff. *variabilis*	Lingshui Bay, Hainan	2007	[91]
axinyssine C (**Bi26)**	*Axinyssa* sp.	Inner coral reef, Andaman Sea, Thailand	2014	[92]
axinyssine D (**Bi27**)	*Axinyssa* sp.	Inner coral reef, Andaman Sea, Thailand	2014	[92]
axinyssine E (**Bi28**)	*Axinyssa* sp.	Inner coral reef, Andaman Sea, Thailand	2014	[92]
axinyssine F (**Bi29**)	*Axinyssa* sp.	Inner coral reef, Andaman Sea, Thailand	2014	[92]
axinyssine G (**Bi30**)	*Axinyssa* sp.	Inner coral reef, Andaman Sea, Thailand	2014	[92]
axinyssine H (**Bi31**)	*Axinyssa* sp.	Inner coral reef, Andaman Sea, Thailand	2014	[92]
axinyssine I (**Bi32**)	*Axinyssa* sp.	Inner coral reef, Andaman Sea, Thailand	2014	[92]
axinyssine J (**Bi33**)	*Axinyssa* sp.	Inner coral reef, Andaman Sea, Thailand	2014	[92]
axinyssine K (**Bi34**)	*Axinyssa* sp.	Inner coral reef, Andaman Sea, Thailand	2014	[92]
axinyssine L (**Bi35**)	*Axinyssa* sp.	Inner coral reef, Andaman Sea, Thailand	2014	[92]
3-formamido-8-methoxybisabolan-9-en-10-ol (**Bi36)**	*Axinyssa*	Micronesia	1999	[90]
	*Axinyssa* sp.	Inner coral reef, Andaman Sea, Thailand	2014	[92]
***Guaianes***				
Guai-6-ene isocyanide (**Gu2**)	Not identified	Wakayama Prefecture, Japan	1988	[93]
Guai-6-ene isothiocyanide (**Gu3**)	Not identified	Wakayama Prefecture, Japan	1988	[93]
	*Acanthella cavernosa*	Tani’s Reef, Gneerings Reef, Mooloolaba, Australia	2007	[32,58]
Guai-6-ene formamide (**Gu4**)	Not identified	Wakayama Prefecture, Japan	1988	[93]
(1S*,4S*,5R*,10S*)-10-Isothiocyanatoguaia-6-ene (**Gu5**)	*Trachyopsis aplysinoides*	Palau	1989	[52]
	*Axinyssa aplysinoides*	Palau	1992	[69]
(1S*,4S*,5R*,10S*)-10-Isocyanoguaia-6-ene (**Gu6**)	*Phyllidiella pustulosa*	Hainan Island, South China Sea	2004	[35]
R=NC (**Gu7**)	*Phyllidia ocellata*	Mudjimba Island, Mooloolaba, Australia	2015	[94]
0R=NCS (**Gu8**)	*Axinyssa* sp.	Sanya Island, Hainan, China	2008	[84]
R=NC (**Gu9**)	*Acanthella acuta*	Bay of Naples, Italy	1987	[62]
R=NCS (**Gu10**)	*Acanthella acuta*	Bay of Naples, Italy	1987	[62]
***Further sesquiterpenoids***				
2-isocyanotrachyopsane (**Fu1**)	*Phyllidia varicosa*	Shimokoshiki Island, Japan	1996	[42]
2-isothiocyanatotrachyopsane (**Fu2**)	*Trachyopsis aplysinoides*	Palau	1989	[52]
	*Axinyssa aplysinoides*	Palau	1992	[69]
2-(formylamino)trachyopsane (**Fu3**)	*Axinyssa aplysinoides*	Malakal Harbor, Palau	1997	[95]
*N*-phenethyl-*N*′-2-trachyopsane (**Fu4**)	*Axinyssa aplysinoides*	Malakal Harbor, Palau	1997	[95]
4α*-*isocyanogorgon-11-ene (**Fu5**)	*Phyllidia varicosa* and *Phyllidia pustulosa*	Negros Island, Cebu Island, San Sebastian, Cebu, Philippines	1991	[86]
4α*-*isothiocyanatogorgon-11-ene (**Fu6**)	*Phyllidia pustulosa*	Negros Island, Cebu Island, San Sebastian, Cebu, Philippines	1991	[86]
4α*-*formamidogorgon-11-ene (**Fu7**)	*Phyllidia varicosa* and *Phyllidia pustulosa*	Negros Island, Cebu Island, San Sebastian, Cebu, Philippines	1991	[86]
(−)-(1*S*,2*R*,5*R*,8*R*)-1-isothiocyanatoepicaryolane (**Fu8**)	*Phyllidia ocellata*	Mudjimba Island, Mooloolaba, Australia	2015	[94]
(1S*,2R*,5S*,6S*,7R*,8S*)-13-isothiocyanatocubebane (**Fu9**)	*Axinyssa aplysinoides*	Ant Atoll, Pohnpei	1992	[69]
(1S*,2S*,5S*,6S*,7R*,8S*)-13-isocyanocubebane (**Fu10**)	*Phyllidia ocellata*	Mudjimba Island, Mooloolaba, Australia	2015	[94]
(1S*,2S*,5S*,6S*,7R*,8S*)-13-isothiocyanatocubebane (**Fu11**)	*Stylissa* sp.	Iriomote Island, Okinawa, Japan	2004	[50]
(−)-(1*S*,5*S*,8*R*)-2-isocyanoclovene (**Fu12**)	*Phyllidia ocellata*	Mudjimba Island, Mooloolaba, Australia	2015	[94]
(−)-(1*S*,2*R*,5*S*,8*R*)-2-isocyanoclovane (**Fu13**)	*Phyllidia ocellata*	Mudjimba Island, Mooloolaba, Australia	2015	[94]
axiplyn A (**Fu14**)	*Axinyssa aplysinoides*	Misali Island, Tanzania	2008	[54]
axiplyn B (**Fu15**)	*Axinyssa aplysinoides*	Misali Island, Tanzania	2008	[54]
axiplyn D (**Fu16**)	*Axinyssa aplysinoides*	Misali Island, Tanzania	2008	[54]
axiplyn E (**Fu17**)	*Axinyssa aplysinoides*	Misali Island, Tanzania	2008	[54]
***Diterpenoids***				
***Acyclic***				
R=NC (**Acy1**)	*Halichondria* sp.	North coast of O’ahu, Hawaii	1974	[39,40]
R=NCS (**Acy2**)	*Halichondria* sp.	North coast of O’ahu, Hawaii	1974	[39,40]
R=NHCHO (**Acy3**)	*Halichondria* sp.	North coast of O’ahu, Hawaii	1974	[39,40]
malonganenone C (**Acy4**)	*Leptogorgia gilchristi*	Ponto Malongane, Mozambique	2006	[96]
	*Euplexaura nuttingi*	Uvinage, Pemba Island, Tanzania	2007	[97]
Δ^11,12^-(*E*) (**Acy5**)	*Euplexaura nuttingi*	Uvinage, Pemba Island, Tanzania	2007	[97]
malonganenone K (**Acy6**)	*Euplexaura robusta*	Wei Zhou Island (Guanxi, China)	2012	[98]
***Kalihinoles***				
kalihinol A (**Kol1**)	*Acanthella* sp.	Apra Harbor, Guam	1984	[100,101,102]
	*Acanthella cavernosa*	Fiji	1988	[106]
	*Acanthella cavernosa*	Pacific Harbor/Benga (=Beqa) Lagoon, Fiji	1994	[99]
	*Acanthella cavernosa*	Yakushima Island, Japan	1995	[111]
	*Acanthella cavernosa*	Yakushima Island, Japan	1996	[114]
	*Phakellia pulcherrima*	Davao, Philippines	1998	[104]
	*Acanthella cavernosa*	Coral reef, Ishigaki Island, Okinawa, Japan	1998	[105]
	*Phyllidiella pustulosa*	Hainan Island, South China Sea	2004	[35]
	*Acanthella* sp.	Yalong Bay, Hainan Province, China	2009	[103]
	*Acanthella cavernosa*	Xisha Islets, South China Sea	2012	[107]
10β-formamido kalihinol A (**Kol2**)	*Acanthella cavernosa*	Hachiji-jima Island, Japan	1996	[20]
	*Acanthella cavernosa*	Xisha Islets, South China Sea	2012	[107]
kalihinol Z (**Kol3**)	*Acanthella* sp.	Fish Patch, Vita Levu, Fiji	1987	[102]
	*Acanthella cavernosa*	Fiji	1988	[106]
	*Phakellia pulcherrima*	Davao, Philippines	1998	[104]
kalihinol X (**Kol4**)	*Acanthella* sp.	Fish Patch, Vita Levu, Fiji	1987	[102]
	*Acanthella cavernosa*	Fiji	1988	[106]
	*Acanthella cavernosa*	Thailand	1991	[49]
	*Phakellia pulcherrima*	Philippines	1998	[104]
	*Acanthella cavernosa*	Dibud, Philippines	2004	[109]
10-*epi*-kalihinol X (**Kol5**)	*Acanthella* sp.	Yalong Bay, Hainan Province, China	2009	[103]
	*Acanthella cavernosa*	Xisha Islets, South China Sea	2012	[107]
kalihinol E (**Kol6**)	*Acanthella* sp.	Apra Harbor, Guam	1987	[102]
	*Acanthella cavernosa*	Hachiji-jima Island, Japan	1996	[20]
	*Phyllidiella pustulosa*	Hainan Island, South China Sea	2004	[35]
	*Acanthella cavernosa*	Xisha Islets, South China Sea	2012	[107]
10β-formamido kalihinol E (**Kol7**)	*Acanthella cavernosa*	Hachiji-jima Island, Japan	1996	[20]
kalihinol O (**Kol8**)	*Acanthella cavernosa*	Xisha Islets, South China Sea	2012	[107]
kalihinol Y(**Kol9**)	*Acanthella* sp.	Fish Patch, Vita Levu, Fiji	1987	[102]
	*Acanthella cavernosa*	Fiji	1988	[106]
	*Acanthella cavernosa*	Thailand	1991	[49]
	*Phakellia pulcherrima*	Philippines	1998	[104]
	*Acanthella cavernosa*	Dibud, Philippines	2004	[109]
Δ^9^-kalihinol Y(**Kol10**)	*Phakellia pulcherrima*	Davao, Philippines	1998	[104]
	*Acanthella cavernosa*	Coral reef, Ishigaki Island, Okinawa, Japan	1998	[105]
kalihinol P (**Kol11**)	*Acanthella cavernosa*	Xisha Islets, South China Sea	2012	[107]
kalihinol S (**Kol12**)	*Acanthella cavernosa*	Xisha Islets, South China Sea	2012	[107]
kalihinol J (**Kol13**)	*Acanthella cavernosa*	Thailand	1991	[49]
	*Acanthella cavernosa*	Dibud, Philippines	2004	[109]
kalihinol I (**Kol14**)	*Acanthella cavernosa*	Thailand	1991	[49]
10-*epi*-kalihinol I (**Kol15**)	*Acanthella cavernosa*	Coral reef, Ishigaki Island, Okinawa, Japan	1998	[105]
	*Acanthella cavernosa*	Xisha Islets, South China Sea	2012	[107]
10β-formamido-5β-isothiocyanato kalihinol A (**Kol16**)	*Acanthella cavernosa*	Hachiji-jima Island, Japan	1996	[20]
	*Acanthella cavernosa*	Xisha Islets, South China Sea	2012	[107]
10β-formamido-5β-isocyanato kalihinol A (**Kol17**)	*Acanthella cavernosa*	Hachiji-jima Island, Japan	1996	[20]
kalihinol Q (**Kol18**)	*Acanthella cavernosa*	Xisha Islets, South China Sea	2012	[107]
kalihinol R (**Kol19**)	*Acanthella cavernosa*	Xisha Islets, South China Sea	2012	[107]
kalihinol B (**Kol20**)	*Acanthella* sp.	Apra Harbor, Guam	1987	[102]
	*Phakellia pulcherrima*	Davao, Philippines	1998	[104]
kalhinol F (**Kol21**)	*Acanthella* sp.	Apra Harbor, Guam	1987	[100,101,102]
	*Acanthella cavernosa*	Fiji	1988	[106]
	*Acanthella* sp.	Cape Sada, Ehime Prefecture, Japan	2003	[108]
	*Acanthella cavernosa*	Davao Gulf, Mindanao, Philippines	2004	[109]
10-formamido kalihinol F (**Kol22**)	*Acanthella cavernosa*	Davao Gulf, Mindanao, Philippines	2004	[109]
15-formamido kalihinol F(**Kol23**)	*Acanthella cavernosa*	Davao Gulf, Mindanao, Philippines	2004	[109]
kalihinol G (**Kol24**)	*Acanthella* sp.	Apra Harbor, Guam	1987	[102]
	*Acanthella cavernosa*	Davao Gulf, Mindanao, Philippines	2004	[109]
10-isothiocyanato kalihinol G (**Kol25**)	*Phakellia pulcherrima*	Philippines	1998	[104]
	*Acanthella cavernosa*	Xisha Islets, South China Sea	2012	[107]
kalihinol H (**Kol26**)	*Acanthella* sp.	Apra Harbor, Guam	1987	[102]
10-*epi*-kalihinol H (**Kol27**)	*Phakellia pulcherrima*	Davao, Philippines	1998	[104]
kalihinol C (**Kol28**)	*Acanthella* sp.	Apra Harbor, Guam	1987	[102]
	*Phakellia pulcherrima*	Philippines	1998	[104]
10-isothiocyanato kalihinol C (**Kol29**)	*Phakellia pulcherrima*	Philippines	1998	[104]
kalihinol D (**Kol30**)	*Acanthella* sp.	Apra Harbor, Guam	1987	[102]
	*Acanthella* sp.	Yalong Bay, Hainan Province, China	2009	[103]
kalihinol T (**Kol31**)	*Acanthella cavernosa*	Xisha Islets, South China Sea	2012	[107]
kalihinol K (**Kol32**)	*Phakellia pulcherrima*	Davao, Philippines	1998	[104]
kalihinol L (**Kol33**)	*Phakellia pulcherrima*	Davao, Philippines	1998	[104]
5,10-bisisothiocyanato kalihinol G (**Kol34**)	*Acanthella cavernosa*	Coral reef, Ishigaki Island, Okinawa, Japan	1998	[105]
isokalihinol B (**Kol35**)	*Acanthella cavernosa*	Kuchihoerabu Island, Satsunan Archipel, Japan	1990	[110]
	*Acanthella cavernosa*	Beau Vallon Beach, Mahé , Seychelles	1994	[112]
isokalihinol F (**Kol36**)	*Acanthella cavernosa*	Fiji	1988	[106]
	*Acanthella cavernosa*	Pacific Harbor/Benga (=Beqa) Lagoon, Fiji	1994	[99]
10-*epi*-isokalihinol F (**Kol37**)	*Acanthella cavernosa*	Beau Vallon Beach, Mahé , Seychelles	1994	[112]
10-*epi*-isokalihinol H (**Kol38**)	*Acanthella cavernosa*	Beau Vallon Beach, Mahé , Seychelles	1994	[112]
kalihinol M (**Kol39**)	*Acanthella cavernosa*	Xisha Islets, South China Sea	2012	[107]
kalihinol N (**Kol40**)	*Acanthella cavernosa*	Xisha Islets, South China Sea	2012	[107]
8-OH-isokalihinol F (**Kol41**)	*Acanthella cavernosa*	Heron Island, Great Barrier Reef, Australia	2000	[31]
***kalihinenes***				
kalihinene (**Ken1**)	*Acanthella cavernosa*	Kuchihoerabu Island, Satsunan Archipel, Japan	1990	[110]
	*Acanthella cavernosa*	Pacific Harbor/Benga (=Beqa) Lagoon, Fiji	1994	[99]
	*Phakellia pulcherrima*	Davao, Philippines	1998	[104]
	*Acanthella cavernosa*	Coral reef, Ishigaki Island, Okinawa, Japan	1998	[105]
	*Phyllidiella pustulosa*	Hainan Island, South China Sea	2004	[35]
	*Acanthella cavernosa*	Davao Gulf, Mindanao, Philippines	2004	[109]
15-formamido kalihinene (**Ken2**)	*Acanthella cavernosa*	Pacific Harbor/Benga (=Beqa) Lagoon, Fiji	1994	[99]
	*Acanthella cavernosa*	Yakushima Island, Japan	1996	[114]
	*Acanthella cavernosa*	Xisha Islets, South China Sea	2012	[113]
10-formamido-kalihinene (**Ken3**)	*Acanthella cavernosa*	Pacific Harbor/Benga (=Beqa) Lagoon, Fiji	1994	[99]
	*Acanthella cavernosa*	Yakushima Island, Japan	1995	[111]
	*Acanthella cavernosa*	Yakushima Island, Japan	1996	[114]
	*Acanthella cavernosa*	Xisha Islets, South China Sea	2012	[113]
10,15-bisformamido kalihinene (**Ken4**)	*Acanthella cavernosa*	Pacific Harbor/Benga (=Beqa) Lagoon, Fiji	1994	[99]
6-hydroxy-kalihinene (**Ken5**)	*Acanthella cavernosa*	Pacific Harbor/Benga (=Beqa) Lagoon, Fiji	1994	[99]
	*Acanthella cavernosa*	Coral reef, Ishigaki Island, Okinawa, Japan	1998	[105]
6-hydroxy-15-formamido-kalihinene (**Ken6**)	*Acanthella cavernosa*	Pacific Harbor/Benga (=Beqa) Lagoon, Fiji	1994	[99]
6-hydroxy-10-formamido-kalihinene (**Ken7**)	*Acanthella cavernosa*	Pacific Harbor/Benga (=Beqa) Lagoon, Fiji	1994	[99]
6-hydroxy-10-formamido-15-isothiocyanato-kalihinene (**Ken8**)	*Acanthella cavernosa*	Pacific Harbor/Benga (=Beqa) Lagoon, Fiji	1994	[99]
Kalihinene A/1,10-di*epi-*kalihinene (**Ken9**)	*Acanthella cavernosa*	Beau Vallon Beach, Mahé , Seychelles	1994	[112]
Kalihinene B/1-*epi*-kalihinene (**Ken10**)	*Acanthella cavernosa*	Beau Vallon Beach, Mahé , Seychelles	1994	[112]
	*Phakellia pulcherrima*	Davao, Philippines	1998	[104]
	*Acanthella cavernosa*	Heron Island, Great Barrier Reef, Australia	2000	[31]
15-isothiocyanato-1-*epi*-kalihinene (**Ken11**)	*Acanthella cavernosa*	Beau Vallon Beach, Mahé , Seychelles	1994	[112]
Kalihinene F (**Ken12**)	*Acanthella cavernosa*	Xisha Islets, South China Sea	2012	[113]
Kalihinene E (**Ken13**)	*Acanthella cavernosa*	Xisha Islets, South China Sea	2012	[113]
Kalihinene X (**Ken14**)	*Acanthella cavernosa*	Yakushima Island, Japan	1995	[111]
	*Acanthella cavernosa*	Yakushima Island, Japan	1996	[114]
	*Acanthella cavernosa*	Xisha Islets, South China Sea	2012	[113]
Kalihinene Y (**Ken15**)	*Acanthella cavernosa*	Yakushima Island, Japan	1995	[111]
	*Acanthella cavernosa*	Yakushima Island, Japan	1996	[114]
	*Acanthella cavernosa*	Xisha Islets, South China Sea	2012	[113]
Kalihinene Z (**Ken16**)	*Acanthella cavernosa*	Yakushima Island, Japan	1995	[111]
	*Acanthella cavernosa*	Yakushima Island, Japan	1996	[114]
***Kalihipyranes***				
Kalihipyran (**Kpy1**)	*Acanthella cavernosa*	Beau Vallon Beach, Mahé , Seychelles	1994	[112]
	*Acanthella cavernosa*	Heron Island, Great Barrier Reef, Australia	2000	[31]
Kalihipyran A (**Kpy2**)	*Acanthella cavernosa*	Yakushima Island, Japan	1996	[114]
	*Acanthella cavernosa*	Xisha Islets, South China Sea	2012	[113]
Kalihipyran B (**Kpy3**)	*Acanthella cavernosa*	Yakushima Island, Japan	1996	[114]
Kalihipyran C (**Kpy4**)	*Acanthella cavernosa*	Xisha Islets, South China Sea	2012	[113]
***Intermediates***				
Cavernene A (**Int1**)	*Acanthella cavernosa*	Xisha Islets, South China Sea	2012	[113]
Cavernene B (**Int2**)	*Acanthella cavernosa*	Xisha Islets, South China Sea	2012	[113]
Cavernene C (**Int3**)	*Acanthella cavernosa*	Xisha Islets, South China Sea	2012	[113]
Cavernene D (**Int4**)	*Acanthella cavernosa*	Xisha Islets, South China Sea	2012	[113]
(**Int5**) planar	*Adocia*	Miyako Island, Japan	1992	[116]
(1S*,6R*,7R*,10S*,11R*)-10-isothiocyanatobiflora-1,14-diene (**Int5**)	*Cymbastela hooperi*	Kelso Reef, Queensland, Australia	1996	[115]
Pulcherrimol (**Int6**)	*Phyllidiella pulcherrima*	Davao, Philippines	1998	[104]
11,12-epoxy-10-isocyano-4,14-bifloradiene (**Int7**)	*Acanthella cavernosa*	Heron Island, Great Barrier Reef, Australia	2000	[31]
11,18-epoxy-10-isocyano-4,14-bifloradiene (**Int8**)	*Acanthella cavernosa*	Heron Island, Great Barrier Reef, Australia	2000	[31]
***Amphilectenes***				
8,15-diisocyano-11(20)-amphilectene/(−)-DINCA (**Amp1**)	*Pseudoaxinella amphilecta*	Glover Reef, Belize	1978	[119]
	*Phyllidiella pustulosa*	Hainan Island, South China Sea	2004	[35]
	*Cribochalina* sp.	Caribbean coast of Mexico	2005	[120]
	*Ciocalapata* sp. (*Halichondriidae*)	Koh-Tao, Surat-Thani province, Thailand	2009	[122]
	*Pseudoaxinella flava*	Sweeting Cay, Grand Bahamas	2011	[128]
	*Svenzea flava*	Great Inagua Island, Bahamas	2013	[133]
	*Svenzea flava*	Cabo Norte, Mona Island, Puerto Rico	2012/2015	[125,160]
8-isocyano-15-formamido-11(20)-amphilectene (**Amp2**)	*Pseudoaxinella amphilecta*	Glover Reef, Belize	1978	[119]
	*Svenzea flava*	Great Inagua Island, Bahamas	2013	[133]
	*Svenzea flava*	Cabo Norte, Mona Island, Puerto Rico	2012/2015	[125,160]
8-isocyano-15-isothiocyanato-11(20)-amphilectene (**Amp3**)	*Cribochalina* sp.	Caribbean coast of Mexico	2005	[120]
(1S*,3S*,4R*,7S*,8S*,12S*,13S*)-8-isocyanato-amphilecta-11(20),14-diene (**Amp4**)	*Stylissa* sp.	Coral reef, Iriomote Island, Okinawa, Japan	2004	[50]
	*Ciocalapata* sp. (*Halichondriidae*)	Koh-Tao, Surat-Thani province, Thailand	2009	[122]
8-isocyano-10,14-amphilectadiene (**Amp5**)	*Halichondria* sp.	Palau	1987	[121]
(3S*,4R*,7S*,8S*,11S*,13S*)-8-isocyanoamphilecta-1(12),14-diene (**Amp6**)	*Stylissa* sp.	Coral reef, Iriomote Island, Okinawa, Japan	2004	[50]
8-isocyanoamphilecta-11(20),15-diene (**Amp7**)	*Ciocalapata* sp. (*Halichondriidae*)	Koh-Tao, Surat-Thani province, Thailand	2009	[123]
8-isocyanato-15-formamidoamphilect-11(20)-ene (**Amp8**)	*Stylissa* cf. massa	Koh-Tao, Surat-Thani province, Thailand	2012	[123]
8-isothiocyanato-15-formamidoamphilect-11(20)-ene (**Amp9**)	*Stylissa* cf. massa	Koh-Tao, Surat-Thani province, Thailand	2012	[123]
Monoamphilectine A (**Amp10**)	*Svenzea flava*	Cabo Norte, Mona Island, Puerto Rico	15 December 2010	[124,125,160]
Monoamphilectine B (**Amp11**)	*Svenzea flava*	Cabo Norte, Mona Island, Puerto Rico	2015	[125]
Monoamphilectine C (**Amp12**)	*Svenzea flava*	Cabo Norte, Mona Island, Puerto Rico	2015	[125]
7-isocyano-11(20),14-epiamphilectadien/(1R*,3S*,4R*,7S*,8S*,12S*,13S*)-7-isocyanoamphilecta-11(20),14-diene (**Amp13**)	*Adocia* sp.		1980	[126]
	*Cymbastela hooperi*	Kelso Reef, Queensland, Australia	1996	[115]
	*Cribochalina* sp.	Caribbean coast of Mexico	2005	[120]
(1R*,3S*,4R*,7S*,8S*,12S*,13S*)-7-formamidoamphilecta-11(20),14-diene (**Amp14**)	*Cymbastela hooperi*	Kelso Reef, Queensland, Australia	2009	[127]
(1R*,3S*,4R*,7S*,8S*,13R*)-7-isocyanoamphilecta-11,14-diene (**Amp15**)	*Cymbastela hooperi*	Kelso Reef, Queensland, Australia	1996	[115]
(1S*,3S*,4R*,7S*,8S*,12S*,13S*)-7-isocyanoamphilecta-11(20),15-diene (**Amp16**)	*Cymbastela hooperi*	Kelso Reef, Queensland, Australia	1996	[115]
	*Adocia* sp.		1980	[126]
	*Ciocalapata* sp. (*Halichondriidae*)	Koh-Tao, Surat-Thani province, Thailand	2009	[122]
	*Svenzea flava*	Cabo Norte, Mona Island, Puerto Rico	2015	[125]
(1S*,3S*,4R*,7S*,8S*,12S*,13S*)-7-Formamidoamphilecta-11(20),15-diene (**Amp17**)	*Cymbastela hooperi*	Kelso Reef, Queensland, Australia	2009	[127]
(1S*,3S*,4R*,7S*,8S*,12S*,13S*-7-isocyano-amphilecta-10,14-diene (**Amp18**)	*Cymbastela hooperi*	Kelso Reef, Queensland, Australia	1996	[115]
(1R*,3S*,4R*,7S*,8S*,12S*,13S*)-7-isocyanoamphilecta-10,14-diene (**Amp19**)	*Stylissa* sp.	Iriomote Island, Okinawa, Japan	2004	[50]
(1R*,3S*,4R*,7S*,8S*,12R*,13R*)-12-hydroxy-7-isothiocyanato-amphilecta-11(20),14-diene (**Amp20**)	*Cymbastela hooperi*	Kelso Reef, Queensland, Australia	1996	[115]
7,15-diisocyano-11(20)-epiamphilectene (**Amp21**)	*Adocia* sp.		1980	[126]
	*Svenzea flava*	Great Inagua Island, Bahamas	2013	[133]
	*Svenzea flava*	Cabo Norte, Mona Island, Puerto Rico	2015	[125]
(1S*,3S*,4R*,7S*,8S*,12S*,13S*)-7-isocyano-15-isothiocyanatoamphilecta-11(20)-ene (**Amp22**)	*Cymbastela hooperi*	Kelso Reef, Queensland, Australia	1996	[115]
7,15-diisocyano-11(20)-amphilectene (**Amp23**)	*Cribochalina* sp.	Caribbean coast of Mexico	2005	[120]
	*Pseudoaxinella flava*	Sweeting Cay, Grand Bahamas	2011	[128]
	*Svenzea flava*	Cabo Norte, Mona Island, Puerto Rico	2012	[160]
7-isocyano-15-isothiocyanato-11(20)-amphilectene (**Amp24**)	*Cribochalina* sp.	Caribbean coast of Mexico	2005	[120]
(1*S*,3*S*,4*R*,7*S*,8*R*,12*S*,13*S*)-7-isocyanoamphilecta-11(20),15-diene (**Amp25**)	*Cribochalina* sp.	Caribbean coast of Mexico	2005	[120]
	*Pseudoaxinella flava*	Sweeting Cay, Grand Bahamas	2011	[128]
	*Svenzea flava*	Cabo Norte, Mona Island, Puerto Rico	2012	[160]
(**Amp26**)	*Pseudoaxinella flava*	Sweeting Cay, Grand Bahamas	2011	[128]
Biflora-4,9,15-triene (**Amp27**)	*Acanthella cavernosa*	Hachiji-jima Island, Japan	1996	[20]
	*Acanthella cavernosa*	Yakusha Island, Japan	1996	[114]
	*Cribochalina* sp.	Caribbean coast of Mexico	2005	[120]
***Cycloamphilectenes***				
8-isocyano-10-cycloamphilectene (**Cam1**)	*Adocia* sp.		1980	[126]
	*Cribochalina* sp.	Caribbean coast of Mexico	2005	[120]
8-isothiocyanato-amphilect-10-ene (**Cam2**)	*Stylissa* sp.	Coral reef, Iriomote Island, Okinawa, Japan	2004	[50]
8-isocyanatocycloamphilect-10-ene (**Cam3**)	*Stylissa* sp.	Coral reef, Iriomote Island, Okinawa, Japan	2004	[50]
(3S*,4R*,7S*,8S*,11S*,13R*)-8-isocyano-1(12)-cycloamphilectene (**Cam4**)	*Halichondria* sp.	Palau	1987	[121]
	*Adocia* sp.		1980	[126]
(1S*,3S*,4R*,7S*,8S*,12S*,13S*)-7-isocyanocycloamphilect-11(20)-ene (**Cam5**)	*Cymbastela hooperi*	Kelso Reef, Queensland, Australia	1996	[115]
(1S*,3S*,4R*,7S*,8S*,12S*,13S*)-7-Formamidocycloamphilect-11(20)-ene (**Cam6**)	*Cymbastela hooperi*	Kelso Reef, Queensland, Australia	2009	[127]
(1S*,3S*,4R*,7S*,8S*,12S*,13S*)-7-isocyanocylcoamphilect-10-ene (**Cam7**)	*Cymbastela hooperi*	Kelso Reef, Queensland, Australia	1996	[115]
(1S*,3S*,4R*,7S*,8R*,13R*)-7-isocyano-11-cycloamphilectene (**Cam8**)	*Halichondria* sp.	Palau	1987	[121]
(1S*,3S*,4R*,7S*,8R*,13R*)-*N*-formyl-7-amino-11-cycloamphilectene (**Cam9**)	*Axinella* sp.	Vanuatu	2002	[129]
(3S*,4R*,7S*,8R*,11S*,12R*,13S*)-7-isocyano-1-cycloamphilectene (**Cam10**)	*Halichondria* sp.	Palau	1987	[121]
***Isocycloamphilectanes***				
diisocyanoadociane/(1*S*,3*S*,4*R*,7*S*,8*S*,11*S*,12*S*,13*S*,15*R*,20*R*)-7,20-diisocyanoisocycloamphilectane (**Ica1**)	*Adocia*	Great Barrier Reef, Townsville, Australia	1976	[130]
	*Cymbastela hooperi*	Kelso Reef, Queensland, Australia	1996	[115]
	*Cribochalina* sp.	Caribbean coast of Mexico	2005	[120]
(1*S*,3*S*,4*R*,7*S*,8*S*,11*S*,12*S*,13*S*,15*R*,20*R*)-20-isocyano-7-isocyanatoisocycloamphilectane (**Ica2**)	*Cymbastela hooperi*	Kelso Reef, Queensland, Australia	1996	[115]
(1*S*,3*S*,4*R*,7*S*,8*S*,11*S*,12*S*,13*S*,15*R*,20*R*)-20-isocyano-7-isothiocyanatoisocycloamphilectane (**Ica3**)	*Cymbastela hooperi*	Kelso Reef, Queensland, Australia	1996	[115]
(1*S*,3*S*,4*R*,7*S*,8*S*,11*S*,12*S*,13*S*,15*R*,20*R*)-20-isocyanato-7-isocyanoisocycloamphilectane (**Ica4**)	*Cymbastela hooperi*	Kelso Reef, Queensland, Australia	1996	[115]
(1*S*,3*S*,4*R*,7*S*,8*S*,11*S*,12*S*,13*S*,15*R*,20*R*)-7-Formamido-10-isocyanoisocycloamphilectane (**Ica5**)	*Cymbastela hooperi*	Kelso Reef, Queensland, Australia	2009	[127]
(1*S*,3*S*,4*R*,7*S*,8*S*,11*S*,12*S*,13*S*,15*R*,20*R*)-7,20-Diformamidoisocycloamphilectane (**Ica6**)	*Cymbastela hooperi*	Kelso Reef, Queensland, Australia	2009	[127]
7,15-diisocyanoadociane (**Ica7**)	*Adocia* sp.		1980	[126]
(1S*,3S*,4R*,7S*,8S*,11R*,12R*,13S*,20S*)-7-isocyanoisocycloamphilect-14-ene (**Ica8**)	*Cymbastela hooperi*	Kelso Reef, Queensland, Australia	1996	[115]
***Neoamphilectenes***				
7-isocyanoneoamphilecta-11,15-diene (**Neo1**)	*Adocia*	Miyako Island, Japan	1992	[116]
***Isoneoamphilectenes***				
7-isocyanoneoamphilecta-1(14),15-diene (**Ina1**)	*Cymbastela hooperi*	Kelso Reef, Queensland, Australia	1996	[115]
	*Svenzea flava*	Great Inagua Island, Bahamas	2013	[133]
7-formamidoisoneoamphilecta-1(14),15-diene (**Ina2**)	*Svenzea flava*	Great Inagua Island, Bahamas	2013	[133]
7-methylaminoisoneoamphilecta-1(14),15-diene (**Ina3**)	*Svenzea flava*	Great Inagua Island, Bahamas	2013	[133]
***Carbonimidic dichlorides***				
Stylotellane A (**Dcl1**)	*Stylotella aurantium*	Coral Garden, Heron Island, Great Barrier Reef, Australia	1997	[71]
Stylotellane B (**Dcl2**)	*Pseudoaxinella pitys*	Indo-pacific	1977	[134]
	*Stylotella aurantium*	Coral Gardens, Heron Island, Great Barrier Reef, Australia	1997	[71]
	*Ulosa spongia*	Wistari Reef, Great Barrier Reef, Australia	2001	[136]
	*Stylotella aurantium*	Iriomate Island, Okinawa, Japan	2001	[137]
Ulosin A (**Dcl3**)	*Ulosa spongia*	Wistari Reef, Great Barrier Reef, Australia	2001	[136]
	*Stylotella aurantium*	Iriomate Island, Okinawa, Japan	2001	[137]
Ulosin B (**Dcl4**)	*Ulosa spongia*	Wistari Reef, Great Barrier Reef, Australia	2001	[136]
Axinyssimide A (**Dcl5**)	*Axinyssa*	Hachijo-jima Island, Japan	1998	[53]
Axinyssimide B (**Dcl6**)	*Axinyssa*	Hachijo-jima Island, Japan	1998	[53]
Axinyssimide C (**Dcl7**)	*Axinyssa*	Hachijo-jima Island, Japan	1998	[53]
(1R*,5S*,6S*)-6,14-dichloro-5-hydroxy-9,3(14)-(*Z)*-axinyssadien-15-yl-carbonimidic dichloride (**Dcl8**)	*Pseudoaxinella pitys*	Indo-pacific	1977	[134]
	*Reticulidia fungia*	Irabu Island, Okinawa, Japan	1999	[141]
	*Stylotella aurantium*	Iriomate Island, Okinawa, Japan	2001	[137]
R=H (**Dcl9**)	*Stylotella aurantium*		2004	[138]
(**Dcl10**)	*Stylotella aurantium*	Iriomate Island, Okinawa, Japan	2001	[137]
(**Dcl11**)	*Pseudoaxinella pitys*	Indo-pacific	1978	[140]
	*Stylotella aurantium*	Iriomate Island, Okinawa, Japan	2001	[137]
(**Dcl12**)	*Pseudoaxinella pitys*	Indo-pacific	1978	[140]
	*Stylotella aurantium*	Iriomate Island, Okinawa, Japan	2001	[137]
(**Dcl13**)	*Pseudoaxinella pitys*	Indo-pacific	1978	[140]
	*Stylotella aurantium*	Iriomate Island, Okinawa, Japan	2001	[137]
(**Dcl14**)	*Stylotella aurantium*	Iriomate Island, Okinawa, Japan	2001	[137]
Reticulidin A (**Dcl15**)	*Reticulidia fungia*	Irabu Island, Okinawa, Japan	1999	[141]
	*Stylotella aurantium*	Iriomate Island, Okinawa, Japan	2001	[137]
Reticulidin B (**Dcl16**)	*Reticulidia fungia*	Irabu Island, Okinawa, Japan	1999	[141]
	*Ulosa spongia*	Wistari Reef, Great Barrier Reef, Australia	2001	[136]
	*Stylotella aurantium*	Iriomate Island, Okinawa, Japan	2001	[137]
Isoreticulidin B (**Dcl17**)	*Pseudoaxinella pitys*	Indo-pacific	1977	[134]
	*Reticulidia fungia*	Irabu Island, Okinawa, Japan	1999	[141]
	*Ulosa spongia*	Wistari Reef, Great Barrier Reef, Australia	2001	[136]
	*Stylotella aurantium*	Iriomate Island, Okinawa, Japan	2001	[137]
***Other marine isonitriles***				
Farnesyl isothiocyanate (**Mis1**)	*Stylotella aurantium*	Heron Island, Great Barrier Reef, Australia	1997	[71]
Farnesyl formamide (**Mis2**)	*Axinyssa* sp.	Sanya, Hainan Province, China	2008	[84]
Isofarnesyl formamide (**Mis4**)	*Axinyssa* sp.	Sanya, Hainan Province, China	2008	[84]
(*R*)-(−)-actisonitrile (**Mis5**)	*Actinocyclis papillatus*	Wei Zhou Island, South China Sea, China	2011	[142]
(2*R*,3*S*)-2-formamide-1,3dihydroxy-octadecane (**Mis6**)	*Gracilaria verrucosa*	Jeju Island, South Korea	2008	[143]
Clavaminol L (**Mis7**)	*Clavelina plegraea*	Bay of Naples, Itlay	2009	[144]
(**Mis8**–**Mis28**)	*Pseudoaxinyssa* sp.	Fiji	1987	[145]
Axinyssina A (**Mis29**)	*Axinyssa* sp.	Inner coral reef, Andaman Sea, Thailand	2014	[92]
Axinyssine B (**Mis30**)	*Axinyssa* sp.	Inner coral reef, Andaman Sea, Thailand	2014	[92]
1-acetyl-4-formamido-4-methylcyclohexane (**Mis31**)	*Axinyssa* sp.	Inner coral reef, Andaman Sea, Thailand	2014	[92]
1-acetyl-4-isocyano-4-methylcyclohexane (**Mis32**)	*Phyllidia* sp.	Colombo, Sri Lanka	1986	[74]
Thiocyanatin A (**Mis33**)	*Oceanapia* sp.	Northern Rottnest shelf, Australia	2001	[146]
Thiocyanatin B (**Mis34**)	*Oceanapia* sp.	Northern Rottnest shelf, Australia	2001	[146]
Thiocyanatin C (**Mis35**)	*Oceanapia* sp.	Northern Rottnest shelf, Australia	2001	[146]
Cylindricine A (**Mis36**)	*Clavelina cylindrica*	Deep Glen Bay, East Coast, Tasmania	1994	[147]
Cylindricine C (**Mis37)**	*Clavelina cylindrica*	Deep Glen Bay, East Coast, Tasmania	1994	[147]
Cylindricine D (**Mis38**)	*Clavelina cylindrica*	Deep Glen Bay, East Coast, Tasmania	1994	[147]
Cylindricine E (**Mis39**)	*Clavelina cylindrica*	Deep Glen Bay, East Coast, Tasmania	1994	[147]
Cylindricine F (**Mis40**)	*Clavelina cylindrica*	Bay of Islands, South Bruny Island, Tasmania	1994	[147]
Cylindricine G (**Mis41**)	*Clavelina cylindrica*	Bay of Islands, South Bruny Island, Tasmania	1994	[147]
Cylindricine H (**Mis42**)	*Clavelina cylindrica*	Tasmania	1995	[148]
Cylindricine I (**Mis43**)	*Clavelina cylindrica*	Tasmania	1995	[148]
Cylindricine B (**Mis44**)	*Clavelina cylindrica*	Deep Glen Bay, East Coast, Tasmania	1994	[147]
Cylindricine J (**Mis45**)	*Clavelina cylindrica*	Tasmania	1995	[148]
Fasicularin (**Mis46**)	*Nephtheis fasicularis*	Micronesia	1997	[149]
Psammaplin B (**Mis47**)	*Psammaplysilla purpurea*		1991	[150]
